# Pak2 Regulation of Nrf2 Serves as a Novel Signaling Nexus Linking ER Stress Response and Oxidative Stress in the Heart

**DOI:** 10.3389/fcvm.2022.851419

**Published:** 2022-03-08

**Authors:** Pablo Binder, Binh Nguyen, Lucy Collins, Min Zi, Wei Liu, Foteini Christou, Xiaojing Luo, Susanne S. Hille, Norbert Frey, Elizabeth J. Cartwright, Jonathan Chernoff, Oliver J. Müller, Kaomei Guan, Xin Wang

**Affiliations:** ^1^Faculty of Biology, Medicine and Health, The University of Manchester, Manchester, United Kingdom; ^2^Institute of Pharmacology and Toxicology, Faculty of Medicine Carl Gustav Carus, Technische Universitaet Dresden, Dresden, Germany; ^3^Department of Internal Medicine III, University of Kiel, Kiel, Germany; ^4^German Center for Cardiovascular Research (DZHK), Partner Site Hamburg/Kiel/Luebeck, Hamburg, Germany; ^5^Department of Cardiology, Angiology and Pneumology, University of Heidelberg, Heidelberg, Germany; ^6^Cancer Biology Program, Fox Chase Cancer Center, Philadelphia, PA, United States

**Keywords:** heart failure, oxidative stress, ER stress, RAAS and oxidative stress, proteostasis, gene therapy

## Abstract

Endoplasmic Reticulum (ER) stress and oxidative stress have been highly implicated in the pathogenesis of cardiac hypertrophy and heart failure (HF). However, the mechanisms involved in the interplay between these processes in the heart are not fully understood. The present study sought to determine a causative link between Pak2-dependent UPR activation and oxidative stress *via* Nrf2 regulation under pathological ER stress. We report that sustained ER stress and Pak2 deletion in cardiomyocytes enhance Nrf2 expression. Conversely, AAV9 mediated Pak2 delivery in the heart leads to a significant decrease in Nrf2 levels. Pak2 overexpression enhances the XBP1-Hrd1 UPR axis and ameliorates tunicamycin induced cardiac apoptosis and dysfunction in mice. We found that Pak2 deletion and altered proteostasis render Nrf2 detrimental by switching from its antioxidant role to renin-angiotensin aldosterone system (RAAS) gene regulator. Mechanistically, Pak2 mediated Hrd1 expression targets Nrf2 for ubiquitination and degradation thus preventing its aberrant activation. Moreover, we find a significant increase in Nrf2 with a decrease in Pak2 in human myocardium of dilated heart disease. Using human-induced pluripotent stem cell-derived cardiomyocytes (iPSC-CMs), we find that Pak2 is able to ameliorate Nrf2 induced RAAS activation under ER stress. These findings demonstrate that Pak2 is a novel Nrf2 regulator in the stressed heart. Activation of XBP1-Hrd1 is attributed to prevent ER stress-induced Nrf2 RAAS component upregulation. This mechanism explains the functional dichotomy of Nrf2 in the stressed heart. Thus, Pak2 regulation of Nrf2 homeostasis may present as a potential therapeutic route to alleviate detrimental ER stress and heart failure.

## Introduction

Heart failure (HF) is a chronic condition where the heart is unable to pump sufficient blood and is commonly the final stage of cardiovascular disease (1). The progression into HF is associated with increased cardiac mass as an adaptive response to maintain cardiac function during increased workload of the ventricle after pressure overload and injury of the cardiac tissue (2). When sustained, cardiac hypertrophy results in maladaptive remodeling which occurs with increased fibrosis, cell death, and chamber dilation leading to reduction in cardiac output and contractility (2). During cardiac hypertrophy protein synthesis is enhanced in order to sustain cell growth (3). Excessive protein synthesis leading to accumulation of misfolded or damaged proteins results in proteotoxic stress and reactive oxygen species (ROS) generation ultimately altering cardiomyocyte function (4). The endoplasmic reticulum (ER) function is key for the maintenance of proteostasis playing a crucial role in the synthesis, folding and translocation of nascent proteins (5). ER-resident transmembrane proteins inositol-requiring kinase 1 (IRE1), PERK (dsRNA-activated protein kinase-like ER kinase), and ATF6 are sensors of ER homeostasis. Accumulation of misfolded proteins in the ER, triggers GRP78 dissociation from these sensors resulting in their activation, termed the unfolded protein response (UPR) (6). UPR activation increases protein folding efficiency and enhances the clearance of defective proteins by induction of the Endoplasmic-reticulum-associated protein degradation (ERAD) *via* the cytosolic ubiquitin-proteasome system (UPS) (7). Additionally, ERAD *via* the UPS can degrade proteins no longer needed, providing temporal regulation of protein activity (8). Failure to restore homeostasis, switches the UPR form a pro-survival into a pro-death response (9). Proteostasis is particularly critical in cardiomyocytes, due to their poor replicative ability (10). Cardiomyocytes depend on highly specialized proteins for electrical conduction and contraction, and their high metabolic demand leads to increased production of proteotoxic agents, particularly ROS (11). Importantly, redox and proteostasis defects have been described as drivers in the pathogenesis of cardiovascular disease, and their genetic and pharmacological regulation present promising therapeutic alternatives (12, 13). However, the mechanisms involved in precise interplay between the UPR and oxidative stress in the heart are yet to be fully understood (14). A critical component of the antioxidant defense is the expression of ROS scavenging and detoxification enzymes, by the conserved Nuclear factor erythroid 2–related factor 2 (Nrf2). Under basal conditions Nrf2 localizes in the cytosol and its activity is regulated *via* ubiquitination by the Cul3/Rbx1-based E3-ubiquitin ligase complex for its proteasomal degradation, facilitated by Kelch-like ECH-associated protein 1 (Keap1) (15). Under stress, Nrf2 dissociates from Keap1, and translocates into the nucleus, activating antioxidant response element (ARE)-driven gene transcription (16). Interestingly, Nrf2 has been described as substrate for the ERAD related E3 ubiquitin ligase, Hrd1 (3-hydroxy-3-methylglutaryl reductase degradation 1), for Keap/Cul3-independet ubiquitination and degradation (17). Thus, the UPR *via* ERAD activation can potentially regulate Nrf2 levels and activity, contributing to the interplay between these major stress pathways. Despite the overall protective role associated with Nrf2 activity there is evidence suggesting that Nrf2 might contribute to the progression of cardiovascular disease (18). However, the cellular context under which Nrf2 acts to either attenuate or aggravate cardiovascular disease is unclear. Recent studies have shown that Nrf2 regulates the expression of renin-angiotensin aldosterone system (RAAS) components in response to stress (19). Nrf2 activation upregulates Angiotensin II signaling in the heart and kidney, which exacerbates cardiac maladaptation and dysfunction and diabetic nephropathy, respectively (19, 20). However, little information is available on the regulatory mechanisms of Nrf2 directing RAAS regulation in the heart.

The p21 activated kinase 2 (Pak2) has been described to have a crucial protective role in the heart (21). Pak2 deletion in zebrafish, leads to hemorrhage due to endothelial defects (22) and endothelial-restricted deletion of Pak2 leads alterations in blood vessel formation (23) highlighting its role in cardiovascular physiology. Our recent studies have reported antihypertrophic properties of cardiac Pak2 through the alleviation of ER stress by regulating the IRE1-XBP1 pathway and ERAD (21). Interestingly, gene array data from Pak2 knock out hearts under tunicamycin-induced ER stress condition revealed dysregulation in genes involved in glutathione metabolism and response to oxidative stress suggesting its participation in the redox response. In this study we aimed to determine a causative link between Pak2-dependent UPR activation and oxidative stress *via* Nrf2 regulation under pathological ER stress. We have identified that Pak2 activation induces Keap1-independent Nrf2 ubiquitination and degradation *via* Hrd1 upregulation, in order to prevent Nrf2 aberrant activation. We have shown that Hrd1 regulation of Nrf2 lays downstream of Pak2 control of XBP1 activity. Furthermore, inhibition of Nrf2 is able to alleviate RAAS dysregulation and cell death in Pak2 deleted cardiomyocytes under sustained ER stress.

## Experimental Procedures

### Animal Models

All laboratory mice and rats in this study were maintained in a pathogen-free facility at the University of Manchester. Animal studies were performed in accordance with the United Kingdom Animals (Scientific Procedures) Act 1986 and were approved by the University of Manchester Ethics Committee and all procedures were conducted under the Project License P3A97F3D1. C57BL/6J mice and Sprague Dawley rats were used. For biochemical, histological, hemodynamics, and echocardiographic studies 5 to 8 animals per group were used. All *in vivo* studies were blinded for both genotype and surgical procedure/treatment during the measurement and analysis stages. Genetically modified and wild-type litter-mates are analyzed in groups of the same age and sex. Only male mice aged between 9 and 10 weeks old were included in all *in vivo* experiments in this study. Cervical dislocation followed by removal of the heart prior to permanent cessation of the circulation was used for euthanasia in mice. Rats were sacrificed by intraperitoneal injection of Pentobarbitone Sodium 20% w/v and 250 μl Heparin Sodium (1,000 U/ml).

### Transverse Aortic Constriction

Mice were anesthetized with 2% isoflurane and ventilated for TAC surgery as previously described (24). Briefly, the transverse aorta between right innominate and left common carotid arteries was subjected to a 27-gauge constriction by a 7-0 Prolene suture to produce an approximate pressure difference of 30–40 mmHg between the two common carotid arteries. Buprenorphine (0.1 mg/kg) was administered for post-operative analgesia. Cardiac function was assessed by echocardiography and the hearts were collected for further analyses.

### Tunicamycin Administration

To examine the effect of ER stress in the heart, tunicamycin (2 mg/kg) (Sigma, T7765) or saline was applied to mice by intraperitoneal injection. Either 2 or 7 days after the injection, cardiac function was examined and the hearts were collected for further analyses.

### Echocardiography

Mice were anesthetized with 1.5% isoflurane and echocardiographic assessment of cardiac function was carried out. Transthoracic M-mode echocardiographic recordings were performed using an Acuson Sequoia C256 system (Siemens) ultrasound machine (24).

### Histology and TUNEL Assay

Five micrometer-thick paraffin embedded heart sections were stained with hematoxylin & eosin to measure cross-sectional areas by randomly selecting 200 cardiomyocytes. TUNEL assay was performed to detect apoptotic cardiomyocytes using the *in situ* Cell Death Detection kit (Roche). A total of 10,000 cardiomyocytes from random fields per heart were analyzed. The images were obtained by Zeiss Axioplan2 microscope and then analyzed by Image J software. *In situ* superoxide production was measured with the oxidative fluorescent dye dihydroethidium (DHE). Cryosections (10 μm) were incubated with DHE for 30 min at 37°C. Images were captured using an Olympus fluorescence microscope. Fluorescence intensities were quantified with ImageJ software.

### Determination of Reduced and Oxidized Glutathione Levels

Intracellular glutathione levels was determined using the Glutathione Assay Kit (#CS0260, Sigma-Aldrich Inc.), following the manufacturer's instructions. For oxidized glutathione (GSSG) determination, reduced glutathione (GSH) was inactivated by the addition of 2-vinylpyridine in the presence of triethanolamine. GSH content was calculated subtracting GSSG from total GSH content. Quantifications were performed by parallel measurements of GSH or GSSG standards, and results were expressed as nmol/mg protein.

### Human Induced Pluripotent Stem Cell-Derived Cardiomyocytes

iWT.D2 and WT1.Bld2 human iPSCs were derived from peripheral blood cells, and maintained with E8 medium (Thermo Fisher Scientific) on Geltrex-coated plates. Cardiomyocyte differentiation of iPSCs was performed as previously described (21). iPSC-derived cardiomyocytes (iPSC-CMs) were cultured in cardiac culture medium (RPMI1640 HEPES Glutamax, B27) up to 120 days for maturation.

### Immunofluorescent Staining

H9C2 cells were seeded onto sterile coverslips. After treatment with tunicamycin (5 μg/ml), cells were fixed in 4% PFA. Cells were permeabilized in 0.2% triton X-solution and blocked with 10% normal donkey serum (Stratech). Fixed cells were incubated with primary antibody (1:200 dilution) against Nrf2 (Abcam, ab62352). Secondary anti-rabbit antibody (Stratech, 1:1,000) conjugated to Alexa Fluor^®^488 was used to detect Nrf2. DAPI was used for nuclear visualization. Images were collected on an Olympus fluorescence microscope. To exclude the possibility of false positive by the primary antibodies, IgG (Cell Signaling, 2729) was used as the control antibody. IgG did not detect any positive staining, which was not represented.

### Luciferase Reporter Assay

To determine Hrd1 promoter activity, H9C2 cells were transfected with human Hrd1 promoter luciferase reporter (−3,324 to −1 relative to transcription start site, containing XBP1s specific UPRE sites, kindly provided by Professor Kazutoshi Mori, Kyoto University, Japan) for 48 h. The luciferase activity was analyzed using Dual-Luciferase Reporter Assay System (Promega, E1910) according to the manufacturer's instructions.

### Cycloheximide Chases

ARCMs were transfected with plasmids expressing Flag-Pak2(T402E), Flag-XBP1s or Myc-Hrd1 in combination with control or Hrd1 siRNA. Forty-eight hours after transfection, 100 μg/mL of cycloheximide was added, and chase experiments were performed in a time window of 1 h after addition of cycloheximide to examine the effect of active Pak2, Hrd1, and XBP1s in the degradation rate of Nrf2. Changes of Nrf2 levels were assessed by immunoblot.

### Immunoprecipitation

Immunoprecipitations were performed with Protein G sepharose (Sigma) following the manufacturer's instruction. Briefly, H9C2 cells or heart tissue were lysed with immunoprecipitation buffer (Tris 50 mM, NaCl 250 mM, 0.25% v/v TritonX- 100, and 10% Glycerol; pH 7.4). Two milligram of the protein extract was immunoprecipitated with antibodies against Nrf2 (Ab137550, Abcam), HA (2367S, Cell Signaling), Hrd1 (Nb100-2526, Novus), or IgG (Cell Signaling, 2729), respectively. Immune complexes were eluted in Laemmli sample buffer (65.8 mM Tris-HCl, pH 6.8, 2.1% SDS, 26.3% glycerol, 0.01% bromophenol blue). Precipitated and input proteins were subjected to SDS-PAGE and immunoblotted using the respective antibodies.

### Cell Viability

Cardiomyocytes cell viability was determined by MTT [3-(4,5-dimethylthiazol-2-yl)-2,5-diphenyltetrazolium bromide] reduction assay. After treatment, cells were incubated in culture media containing MTT solution (0.5 mg/mL) for 3 h at 37°C. The purple formazan product in the cells was solubilized by addition of acidic isopropanol (0.04 M HCl) and the absorbance was measured at 570 nm with background subtraction at 630 nm. Cell viability was expressed as the percentage of untreated controls.

### Lysate Preparation and Immunoblotting

Total protein from tissues or cells was obtained with Triton lysis buffer (Tris 20 mM, NaCl 137 mM, EDTA 2 mM, 1% Triton X-100, β-glycerophosphate 25 mM, Na_3_VO_4_ 1 mM, phenylmethanesulfonylfluoride 1 mM, aprotinin 1.54 μM, leupeptin 21.6 μM, 10% glycerol; pH 7.4). Protein concentration was determined by Bio-Rad protein assay. Protein extracts (20 μg) were subject to immunoblot analyses with antibodies listed in Supplementary Table 1. Immune-complexes were detected by enhanced chemiluminescence with anti-mouse or anti-rabbit immunoglobulin-G coupled with horseradish peroxidase.

### Quantitative Real-Time Polymerase Chain Reaction

Total RNA was extracted from ventricular tissues or cells using Trizol (Invitrogen) followed by conversion to cDNA. All primers were purchased from Qiagen. Real-time quantitative PCRs were performed using SYBR Select PCR Master Mix according to the manufacturer's instruction in the Step One Plus PCR System (Applied Biosystems). The fold change was analyzed using the 2-^ΔΔ^CT method (25). The level of expression was normalized to *Gapdh*.

### Human Heart Tissues

The study complies with the Declaration of Helsinki and was reviewed and approved by the ethics committee of the Technische Universität Dresden (Az: EK 422092019 and EK EK 446122011. LV tissue was obtained from dilated heart failure patients (*n* = 5), and tissue from non-failing donor hearts (*n* = 5) served as reference.

### Data Analysis

Sample sizes were calculated based on available comparable data to achieve 90% power at a 5% significance level. For all *in vivo* and *in vitro* analyses, normal distribution (Gaussian distribution) was first determined by Shapiro–Wilk test. Data were then analyzed using Student's *t*-test for comparisons between two experimental groups or one- or two-way ANOVA with Bonferroni *post-hoc* tests for comparisons among multiple experimental groups. Data are expressed as scatter plots indicating mean ± S.E.M. *P*-values *p* < 0.05 were considered statistically significant.

## Results

### Enhanced Oxidative Stress and Cell Death Are Promoted in Pak2-CKO Hearts Under Stress

**Figure 1:**
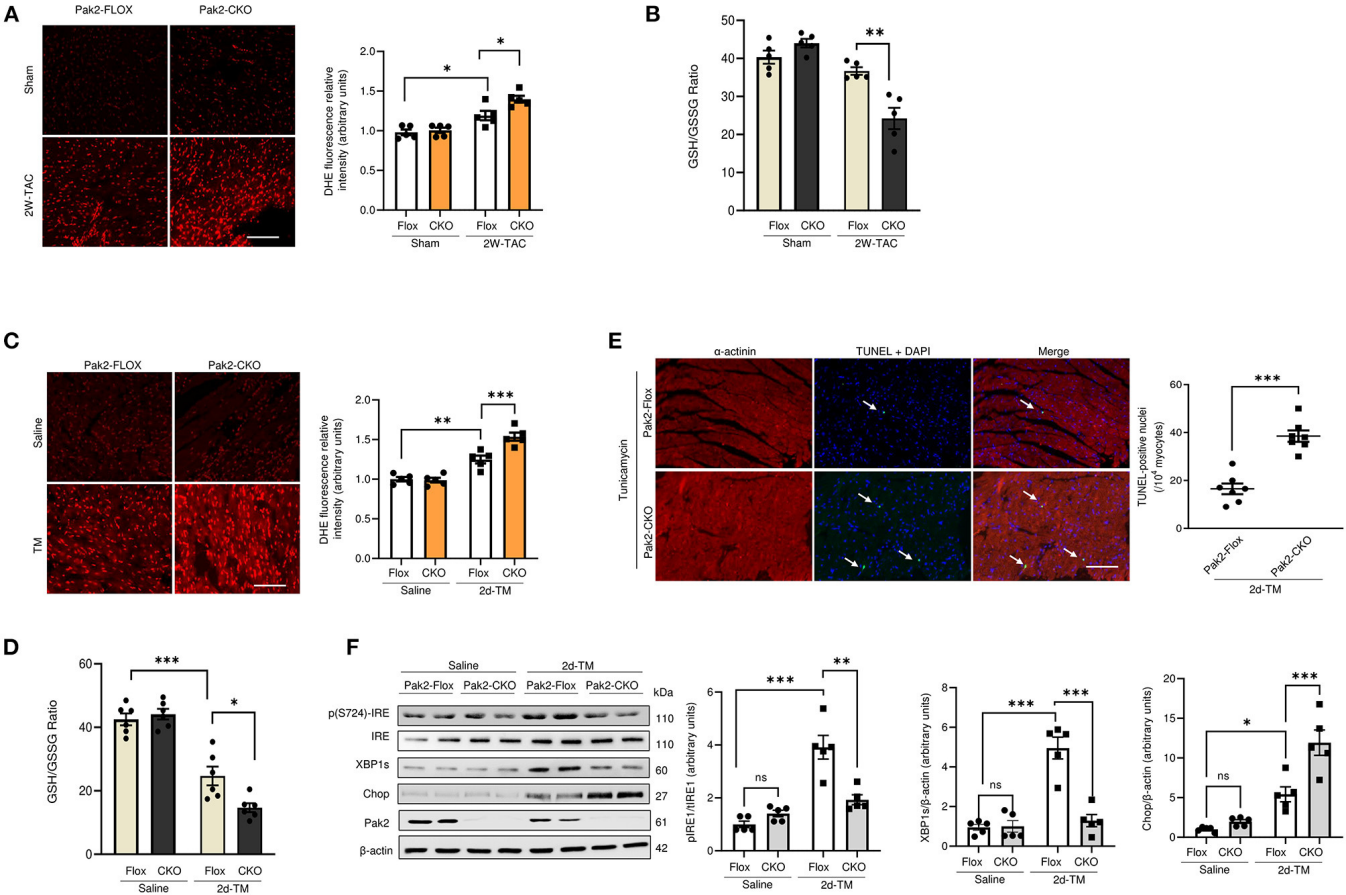
Pak2 cardiac deletion enhances ER stress induced cardiac dysfunction, defective UPR and oxidative stress. Oxidative stress analysis of heart tissue was performed using dihydroethidium (DHE) **(A)** (scale bar = 20 μm, *n* = 5), and reduced glutathione to oxidized glutathione (GSH/GSSG) ratio **(B)** in TAC-stressed hearts (*n* = 5–6). Oxidative stress analysis of heart tissue using DHE **(C)** (scale bar = 20 μm, *n* = 5) and GSH/GSSG ratio (*n* = 6) **(D)** in TM-injected mouse hearts. **(E)** TUNEL assay of hearts after 2 days of TM injection (scale bar = 20 μm), arrows indicate TUNEL positive nuclei (*n* = 7). **(F)** Immunoblots and quantification of UPR components in TAC-stressed hearts (*n* = 5). Student's *t*-test or 2-way ANOVA with Bonferroni correction for *post-hoc* comparisons were used for analyses. Data presented as mean ± SEM. *P < 0.05, **P < 0.01, ***P < 0.001.

In our recent study, we showed exacerbated hypertrophy in cardiac-specific Pak2 knockout mice (Pak2-CKO) under pressure overload by transverse aortic constriction (TAC) by an increase of deleterious ER stress response in the heart (21). Pak2-CKO mice show increased myocardial cell death (Supplementary Figure 1A), decreased cardiac function (Supplementary Figure 1B) and dysregulation of the IRE-XBP1 branch of the UPR (Supplementary Figure 1C) leading to substantial ER stress and aggregation of ubiquitinated proteins in TAC stressed hearts (Supplementary Figures 1C,D). Intriguingly, compared to their Pak2-Flox littermates, Pak2 deleted mice under TAC show a significant increase in reactive oxygen species (ROS) (Figure 1A) and imbalance in reduced glutathione content by determination of the GSH/GSSG ratio in hearts (Figure 1B; Supplementary Figures 2A,B). To directly investigate the effects of ER stress on cardiac function, acute stress was induced in mice with tunicamycin *via* intraperitoneal injection (2 mg/kg), which inhibits N-linked glycosylation of proteins in the ER (26). The use of tunicamycin, in combination to anti-cancer drugs, has been proposed to enhance the efficacy of chemotherapy (27). However, the systemic ER stress produced leads to increased cardiomyocyte apoptosis and development of cardiomyopathy (28). Similar to what we observed with TAC, Pak2 cardiac depletion in mice subjected to ER stress show increased ROS (Figure 1C), imbalance of GSH/GSSG ratio (Figure 1D; Supplementary Figures 2C,D) and increased cell death (Figure 1E). Moreover, cardiac deletion of Pak2 exacerbates ER stress, protein aggregation (Figure 1F; Supplementary Figure 2E) and increased cardiac dysfunction (Supplementary Figure 2F) in tunicamycin treated mice. Overall, this data suggests that Pak2 depletion, in addition to induce UPR imbalance, might impair the redox response in hearts under pathological stress.

### Cardiac Pak2 Depletion Induces Nrf2 Upregulation Under Sustained ER Stress

**Figure 2:**
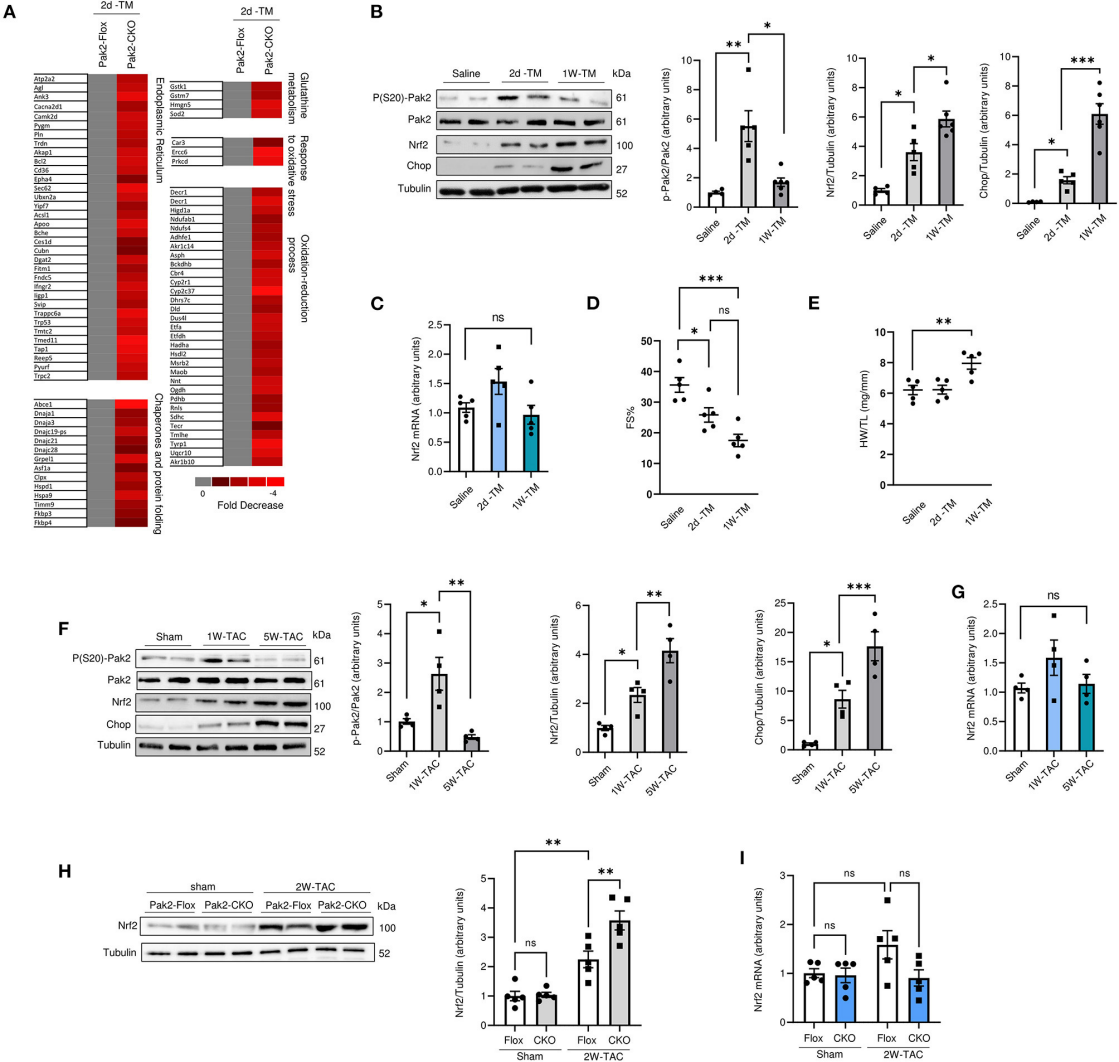
Nrf2 expression is upregulated in Pak2 depleted hearts under sustained ER stress. **(A)** Differentially expressed genes by Affymetrix gene array from Tunicamucin treated mice. Gene expression profiling in Pak2-Flox vs. Pak2-CKO hearts was normalized to gray. Red represents decreased expression (*n* = 2). **(B)** Immunoblots and quantification from hearts of C57BL/6N mice subjected to 2 or 7 days TM intraperitoneal injection (*n* = 5). (**C)** Quantitative polymerase chain reaction (qPCR) analysiss of Nrf2 transcript in TM-stressed hearts (*n* = 5). **(D)** Echocardiographic analysis of mice after TM injection for 2 days and 1 week (*n* = 5). **(E)** Heart weight/tibia length ratio (HW/TL) determined an increase in cardiac hypertrophy following TM injection (*n* = 5). **(F)** Immunoblots and quantification of hearts from mice subject to TAC (*n* = 4). **(G)** qPCR analysis of Nrf2 transcript in hearts of mice subject to 1 or 5 week TAC (*n* = 4). Immunoblots **(H)** and qPCR **(I)** analysis of Nrf2 levels in TAC-stressed Pak2-CKO hearts (*n* = 5). 1-way or 2-way ANOVA with Bonferroni correction for *post-hoc* comparisons were used for analyses. Data presented as mean ± SEM. *P < 0.05, **P < 0.01, ***P < 0.001.

Pak2 promotes a protective response to ER stress *via* activation of IRE1-XBP1 branch of the UPR in cardiomyocytes (21). Moreover, Pak2 can be activated in response to hydrogen peroxide in isolated cardiomyocytes (Supplementary Figure 3A). Pak2 depletion hinders IRE1-XBP1 activation in response to oxidative stress and leads to an increase in the apoptotic marker CHOP (Supplementary Figure 3B), suggesting that Pak2 could be protective against oxidative stress in cardiomyocytes *via* IRE-XBP1 activation in response to ROS-induced ER stress. The observation that oxidative stress is exacerbated by Pak2 depletion in the heart is supported by gene array analysis preformed in Pak2-CKO hearts under ER stress. Among those transcripts significantly altered compared to Pak2-FLOX control (*P* < 0.05), in addition to ER stress response, there is a significant downregulation in transcripts involved in oxido-reduction and glutathione metabolism according to functional annotation by DAVID version 6.2 (Figure 2A). Prompted by this data, we decided to investigate the interrelationship between Pak2 activation and key redox pathways. Under acute ER stress (2 days tunicamycin injection), both Pak2 phosphorylation and the expression of the redox regulator Nrf2 show significant increase in mouse hearts (Figure 2B). Prolonged ER stress (1 week tunicamycin injection) leading to cardiac dysfunction (Figure 2D; Supplementary Figure 4A) and increased hypertrophy (Figure 2E; Supplementary Figure 4B) results in decrease in cardiac Pak2 phosphorylation (Figure 2B). Intriguingly, these hearts, showed further increase in Nrf2 protein levels with no change in Nrf2 mRNA (Figures 2B,C). Similarly, using TAC to provoke cardiac disease-mimetic ER stress, we observe a significant reduction in Pak2 phosphorylation and further upregulation of Nrf2 in the hearts of mice after 5 weeks of pressure overload (Figures 2F,G), showing severe cardiac dysfunction (Supplementary Figure 4C) and CHOP upregulation (Figure 2F). Interestingly, Pak2 deleted (Pak2-CKO) hearts show enhanced Nrf2 upregulation as early as 2 weeks after TAC (Figure 2H), suggesting a potential role for Pak2 in Nrf2 expression. Importantly, the increase in Nrf2 protein levels under ER stress in wild type, Pak2-Flox and Pak2-CKO hearts occurred with no significant change in Nrf2 mRNA levels (Figures 2C,G,I). These results indicate that whereas under initial ER stress there is a simultaneous increased Pak2 phosphorylation and Nrf2 expression, prolonged ER stress or pressure overload show an inverse correlation between Pak2 activation and Nrf2 protein levels in the dysfunctional heart.

### Pak2 Activation Promotes Nrf2 Downregulation and Cardiac Protection Under Sustained ER Stress

**Figure 3:**
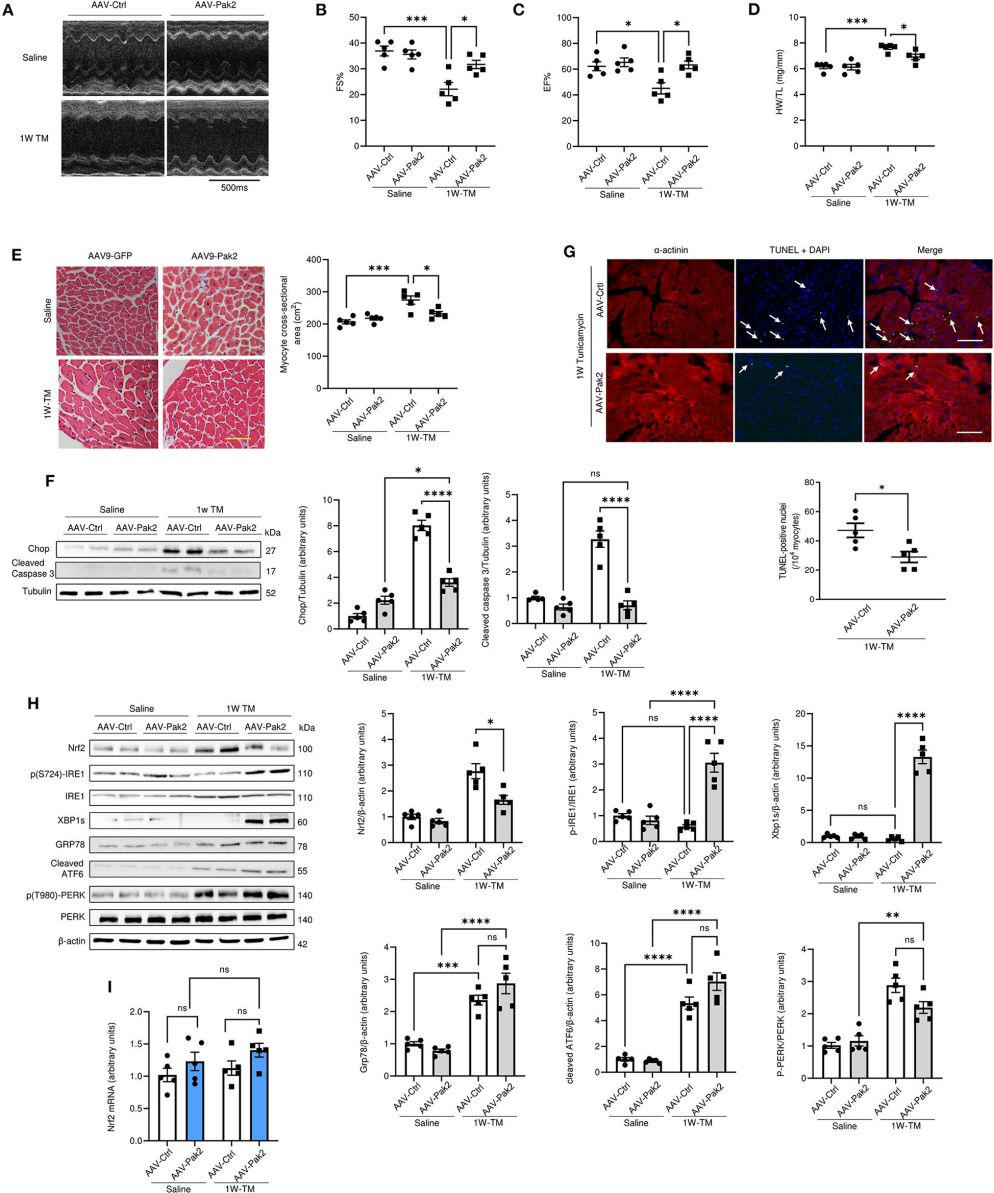
Activated Pak2 is protective against sustained ER stress and decreases Nrf2 accumulation in the heart. **(A)** M-mode images captured by echocardiography were used to determine cardiac function in AAV-9-Pak2-T402E-injected C57BL/6N mice. **(B,C)** Echocardiographic analysis after 1 week Tunicamycin (TM, 2 mg/kg) injection (*n* = 5). Detailed Echocardiographic data available in Supplementary Table 3. Cardiac hypertrophy was determined by assessing mice heart weight/tibia length ratio (HW/TL) **(D)** and myocyte cross-sectional areas **(E)** (scale bar = 20 μm, *n* = 5). Cell death in was evaluated by immunoblots and quantification of Chop and cleaved caspase 3 **(F)** and by TdT-mediated dUTP nick end labeling (TUNEL) assay **(G)** (scale bar = 20 μm) arrows indicate TUNEL positive nuclei (*n* = 5). **(H)** Immunoblots and quantification of Nrf2 and UPR components in Tunicamycin-stressed hearts. **(I)** qPCR analysis of Nrf2 transcript in AAV9-Pak2 -injected hearts (*n* = 5). 2-way ANOVA with Bonferroni correction for *post hoc* comparisons were used for analyses. Data presented as mean ± SEM. *P < 0.05, **P < 0.01, ***P < 0.001, ****P < 0.0001.

To examine the effects of Pak2 activation in the heart, cardiac-specific expression of constitutively active Pak2 was achieved *via* AAV9-mediated gene delivery in mice. C57BL/6 mice were injected *via* tail vein with TnT promoter-driven AAV9-Pak2-T402E (AAV9-Pak2) or AAV9-GFP prior to tunicamycin treatment for 1 week. Consistent with our previous findings, overexpression of active Pak2 prevents the ER stress induced progression into cardiac hypertrophy and dysfunction (Figures 3A–E; Supplementary Figures 5A,B). Pak2 overexpression also significantly alleviates cardiac cell death and fibrosis induced by tunicamycin (Figures 3F,G; Supplementary Figure 5C). Furthermore, in response to tunicamycin-induced ER stress, active Pak2 is able to activate the IRE1-XBP1s branch of the UPR (Figure 3H) with no significant effect over ATF6 or PERK. Interestingly, cardiac expression of active Pak2 prevents the increase in Nrf2 protein levels observed with no significant change in Nrf2 transcript level (Figure 3I). Overall, we further confirmed the protective role of cardiac specific activation of Pak2 under ER stress. Additionally, the inverse relation observed on the expression of Nrf2 and Pak2 *in vivo* might suggest a negative role for Pak2 in Nrf2 expression under cardiac ER stress.

### Pak2 Depletion Promotes Aberrant Activation of Nrf2 Under ER Stress in Cardiomyocytes

**Figure 4:**
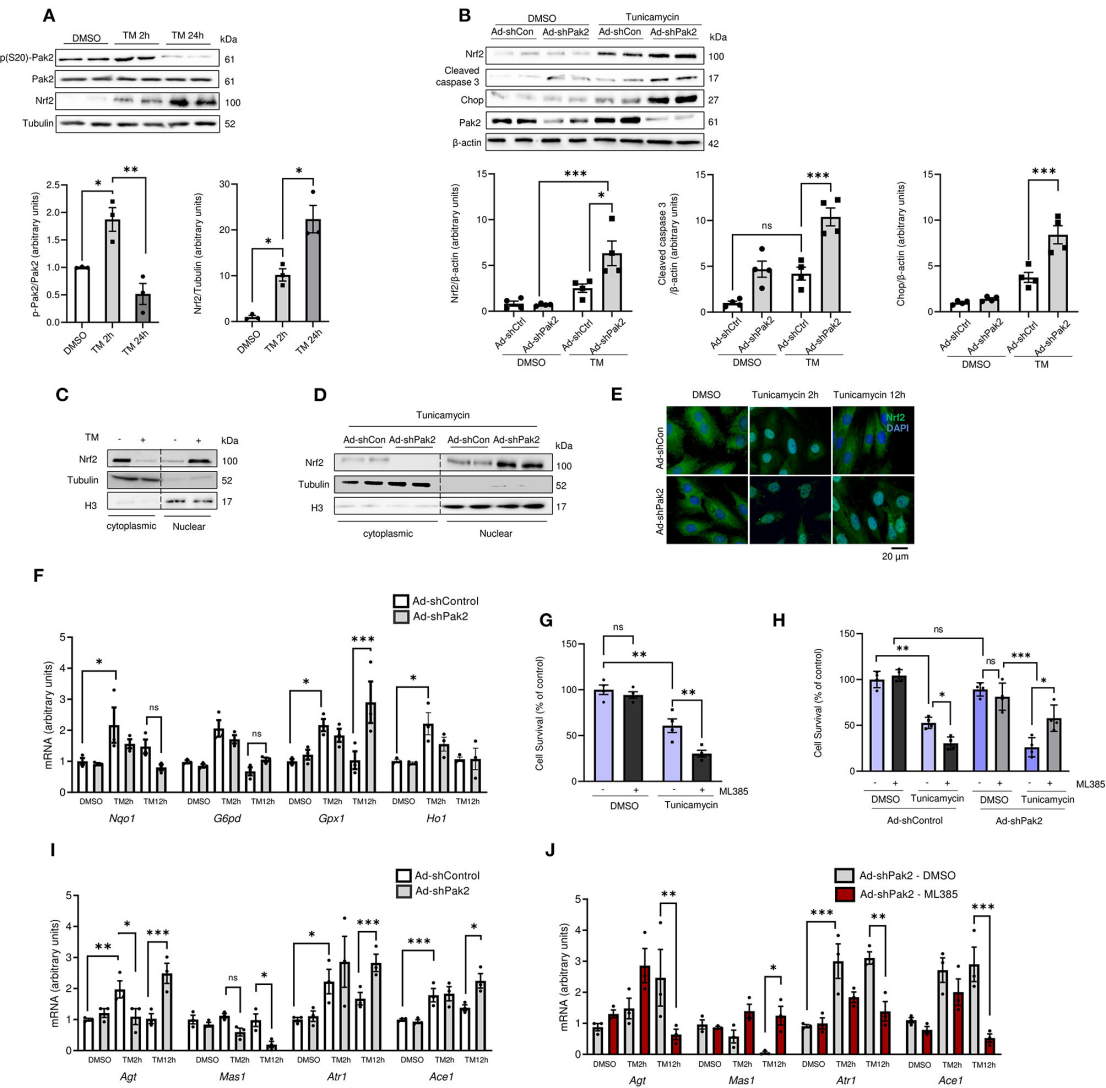
Pak2 depletion promotes aberrant Nrf2-dependent RAS gene upregulation under ER stress. **(A)** Immunoblots and quantification of phosphorylated Pak2 and Nrf2 in ARCMs subjected to tunicamycin (TM)-induced ER stress (*n* = 3). **(B)** Immunoblots of Pak2-knockdown (Ad-shPak2) ARCMs under TM treatment (*n* = 4). Nrf2 subcellular localization was assessed by immunoblot analysis using fractional protein preparation **(C)** and immunofluorescence analysis in Ad-shPak2 H9C2 cells under TM stress **(D,E)** (*n* = 3, Scale bar: 20μm). **(F)** Quantitative polymerase chain reaction (qPCR) was used to assess Nrf2 conventional transcriptional targets (*n* = 3). **(G)** Cell viability was determined by MTT [3-(4,5-dimethylthiazol-2-yl)-2,5-diphenyltetrazolium bromide] reduction assay in WT **(G)** or Ad-shPak2 **(H)** cardiomyocytes under TM stress in combination with Nrf2 inhibition with ML385 (*n* = 4). **(I, J)** qPCR analyses was used to assess RAAS components *Agt, Atr1, Mas1*, and *Ace1* in Ad-shPak2 cardiomyocytes under ER stress (*n* = 3). 2-way ANOVA with Bonferroni correction for *post-hoc* comparisons were used for analyses. Data presented as mean ± SEM. *P < 0.05, **P < 0.01, ***P < 0.001.

To elucidate the link between the Pak2 activation and Nrf2 downregulation, a cellular cardiomyocyte model was used. Similar to our *in vivo* observations, acute tunicamycin treatment (5 μg/mL for 2 h) induced an increase in Pak2 phosphorylation and Nrf2 levels in isolated adult rat cardiomyocytes (ARCMs). Sustained ER stress for 24 h, leading to exacerbated protein aggregation (Supplementary Figure 6A) disrupts Pak2 activation and results in further increase in Nrf2 accumulation (Figure 4A; Supplementary Figure 6B). Adenovirus mediated shRNA-ablation of Pak2 in cardiomyocytes under ER stress greatly exacerbates ubiquitinated protein accumulation (Supplementary Figure 6C) and induces an increase in Nrf2 levels accompanied by an increase in the apoptotic markers CHOP and cleaved caspase 3 (Figure 4B). As Nrf2 is crucial in the maintenance of redox homeostasis, we tested whether the increase in Nrf2 expression observed is associated to an increase in Nrf2 nuclear translocation and antioxidant transcriptional activity. Tunicamycin induced an increase in Nrf2 nuclear localization after 2 h of treatment as detected by immunoblot after cell fractionation in rat cardiomyocytes (Figure 4C). Moreover, Pak2 deletion promotes an increase in nuclear Nrf2 following tunicamycin treatment (Figure 4D). Rat cardiomyocytes Immunofluorescence showed a retention and accumulation of Nrf2 in the nucleus following tunicamycin treatment in Pak2 depleted cells (Figure 4E). Functionally, qPCR analyses on the Nrf2 transcriptional targets *Nqo1, Ho1 G6pd* and *Gpx1*, showed initial increase at 2 h tunicamycin treatment whilst a significant decrease in their expression is observed at 12 h, consistent with the observed Nrf2 localization (Figures 4E,F). Whereas, Pak2 ablation did not significantly affect the mRNA expression of *Nqo1, Ho1*, and *G6pd*, induction of *Gpx1* by sustained ER stress is exacerbated by Pak2 knockdown (Figure 4F). This suggests that the accumulated Nrf2 observed under Pak2 knockdown in cardiomyocytes could be active toward specific transcriptional targets, thus Nrf2 under Pak2 depletion and the subsequent ER stress environment could be triggering transcriptional responses different from its protective antioxidant role. Of note, early Nrf2 inhibition by ML385 (2 μM) in cardiomyocytes prior to treatment with tunicamycin show a significant reduction in cell survival (Figure 4G). Interestingly, when cardiomyocytes were subjected to ER stress induced by tunicamycin following Pak2 depletion, Nrf2 inhibition after the onset of ER stress appears to partially enhance cardiomyocytes survival (Figure 4H), indicating that in Pak2 depleted cardiomyocytes, excessive Nrf2 might play a detrimental role. Recent reports have linked aberrant Nrf2 activity with a pathological increase in RAAS gene expression (20). Promoter sequence analyses show conserved predicted Nrf2 binding sites for key components of the RAAS system (Supplementary Figure 7A). Thus, we wanted to test whether the activation of Nrf2 in the context of maladaptive ER stress is associated with dysregulation of angiotensin signaling. qPCR analyses showed upregulation of *Agt, Atr1*, and *Ace1* in response to ER stress. Interestingly, whereas Pak2 depletion has little effect on the mRNA expression at 2 h of tunicamycin, following 12 h an exacerbated induction of *Agt, Atr1*, and *Ace1* mRNA expression and downregulation in *Mas1* expression was observed (Figure 4I; Supplementary Figure 7B). Furthermore, this aberrant overexpression of RAAS transcripts observed in Pak2 depleted cells, can be reverted by inhibition of Nrf2 (Figure 4J). Importantly, both the Angiotensin II receptor blocker Losartan and the angiotensin-converting enzyme (ACE) inhibitor Enalapril were able to significantly reduce apoptotic markers and increase cell survival after 24 h tunicamycin in Pak2 depleted cardiomyocytes (Supplementary Figures 8A–D). This suggests that the detrimental effects of Pak2 deletion under sustained ER stress is at least in part due to local dysregulation of angiotensin signaling. Overall, this data suggests that in conditions of Pak2 depletion and sustained ER stress, Nrf2 is retained in the nucleus and has a detrimental effect in cardiomyocytes by inducing an aberrant expression of RAAS genes.

### Pak2 Regulates Nrf2 Accumulation and Activity *via* XBP1-Hrd1 Activation

Increased Nrf2 levels in Pak2 depleted hearts after pressure overload or ER stress occurred without significant changes in Nrf2 mRNA levels (Figures 2C,G,I). Similarly, cardiac overexpression of active Pak2 resulted in a decrease in Nrf2 protein (Figure 3I). This suggested that Pak2 might promote Nrf2 degradation. We had previously reported Pak2 as an ERAD regulator *via* XBP1-Hrd1 activation (21). Interestingly, Hrd1 expression follows a similar pattern to Pak2 activation following both direct ER stress and pressure overload (Supplementary Figures 9A,B). Moreover, Pak2 *in vivo* and *in vitro* depletion ablates Hrd1 expression in response to stress (Supplementary Figures 9C–E) and cardiac-specific overexpression of constitutively active Pak2 promotes cardiac Hrd1 upregulation in mice under systemic ER stress (Supplementary Figure 9F). Hrd1 targets misfolded proteins of the endoplasmic reticulum for ubiquitination and subsequent proteasomal degradation and it has been described as a novel Nrf2 ubiquitin E3 ligase (17). Led by the above results we decided to test whether the XBP1-Hrd1 axis of the UPR is responsible for the Pak2 mediated downregulation of Nrf2 in the heart. Consistent with this idea, the increased Nrf2 accumulation and Hrd1 downregulation observed in stressed cardiomyocytes under Pak2 knockdown can be reverted by overexpression of XBP1s *in vitro* in tunicamycin treated cardiomyocytes (Supplementary Figure 10A). Furthermore, restoring XBP1s activity *via* AAV9 injection, increases in Hrd1 levels in Pak2 knockout mice 4 weeks after TAC, and reverts the Nrf2 accumulation in Pak2-CKO hearts (Supplementary Figure 10B). Additionally, XBP1s overexpression can revert Pak2 knockdown mediated Nrf2 nuclear accumulation (Supplementary Figure 10C) and the aberrant transcriptional activity observed in RAAS components mRNA (Supplementary Figures 10D,E) resulting in enhanced cardiomyocyte survival (Supplementary Figure 10). Overall, these data indicate that Pak2 *via* XBP1 activation is able to relieve the aberrant activation of Nrf2 in cardiomyocytes. This further supports the role of Pak2 as a negative regulator of Nrf2 *via* activation of the IRE1-XBP1-Hrd1 axis.

### Pak2 Activation Enhances Hrd1 Mediated Nrf2 Ubiquitination and Degradation

**Figure 5:**
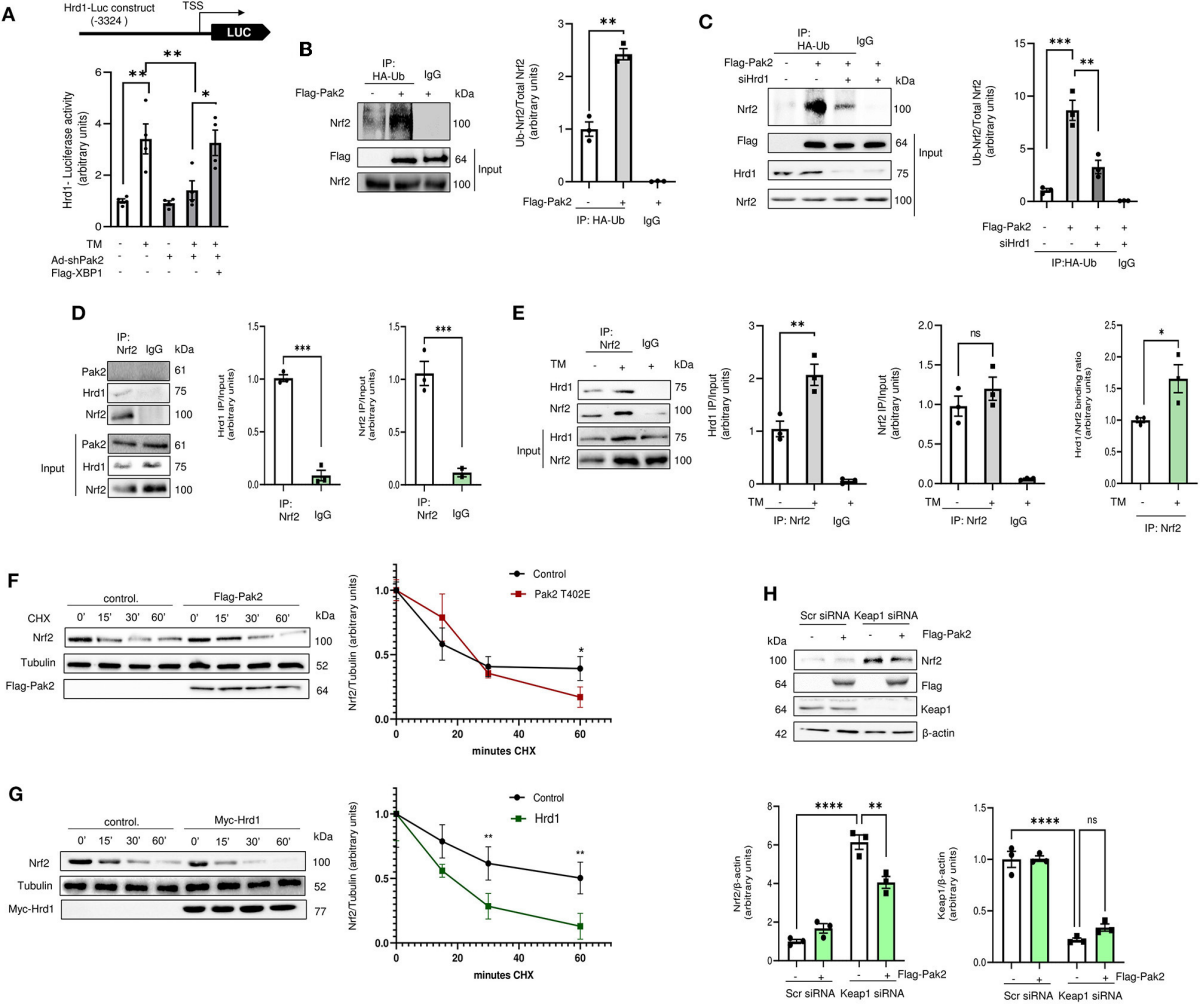
Pak2-dependent IRE-XBP1 activation leads to enhanced Hrd1-mediated Nrf2 ubiquitination and degradation. **(A)** Hrd1 reporter luciferase activity was detected in Pak2 knockdown (Ad-shPak2) H9C2 cells in response to Tunicamycin in XBP1s expressing (Flag-XBP1) cardiomyocytes (*n* = 4). **(B)** The effect of active Pak2 overexpression and Hrd1 knockdown (siHrd1) **(C)** on Nrf2 ubiquitination was determined by HA-ubiquitin (HA-ub) immunoprecipitation and Nrf2 immunoblot (*n* = 3). Association of endogenous Nrf2 with Hrd1 was observed by immunoprecipitation in H9C2 cells under basal conditions **(D)** and in Tunicamycin stressed cells **(E)**. The effect of tunicamycin in Hrd1 and Nrf2 binding is expressed as the change in IP/input ratio for Hrd1 and Nrf2 (*n* = 3). Nrf2 degradation in cardiomyocytes was assessed by cyclohexamide (CHX, 100 μg/mL) chases. Immunoblots showed that Flag-Pak2-T402E **(F)** and Myc-Hrd1 **(G)** enhance the clearance of Nrf2 in ARCMs (*n* = 3). **(H)** Immunoblots showing the effect of Keap1 knockdown (Keap1 siRNA) on Pak2-mediated Nrf2 downregulation (*n* = 3). Student's *t*-test or 2-way ANOVA with Bonferroni correction for *post-hoc* comparisons were used for analyses. Data presented as mean ± SEM. *P < 0.05, **P < 0.01, ***P < 0.001, ****P < 0.0001.

Pak2 knockdown significantly decreases tunicamycin-induced Hrd1 transcription as measured by a luciferase reporter gene carrying human Hrd1 promoter region (−1 to −3,324), which can be restored by overexpression of XBP1s (Figure 5A; Supplementary Figure 11A). To address the role of Hrd1 on Nrf2 degradation, Nrf2 ubiquitination was assessed by immunoprecipitation of HA-tagged ubiquitin and detection of Nrf2 by immunoblot in cardiomyocytes. We observed that overexpression of active Pak2 enhances Nrf2 ubiquitination (Figure 5B; Supplementary Figure 11B). Importantly, Pak2 mediated Nrf2 ubiquitination is abolished by both, the Hrd1 inhibitor LS-102 (10 μM) (Supplementary Figure 11C) and by Hrd1 knockdown *via* siRNA (Figure 5C). Immunoprecipitation assays showed that Nrf2 and Hrd1, but not Pak2 directly interact in cardiomyocytes (Figure 5D; Supplementary Figure 11D), and this interaction is enhanced by ER stress (Figure 5E). To confirm that overexpression of Pak2 mediated Nrf2 degradation, we performed cycloheximide (CHX) chase experiments. We found that overexpression of active Pak2 enhances the clearance of Nrf2 within a window of an hour (Figure 5F). Importantly, Pak2 triggered Nrf2 downregulation can be prevented by inhibition of Hrd1 (Supplementary Figure 11E), and Hrd1 knockdown abolishes Pak2-induced Nrf2 clearance (Supplementary Figure 11F). Consistently, direct overexpression of Hrd1 significantly accelerates Nrf2 degradation in cardiomyocytes (Figure 5G). Nrf2 expression is mainly regulated by Keap1, which facilitates Cullin 3 mediated Nrf2 ubiquitination for proteasomal degradation. Importantly, neither Keap1 nor Cullin 3 expression are affected by Pak2 (Supplementary Figures 12A,B) and silencing Keap1 does not seem to impede Pak2 mediated Nrf2 downregulation (Figure 5H). Overall, we found that Pak2 prevents Nrf2 aberrant activation by enhancing its ubiquitination and degradation *via* the IRE1-XBP1-Hrd1 axis in response to ER stress.

### Nrf2 Downregulation by Pak2 Alleviates Cell Death in Human Cardiomyocytes Under ER Stress

**Figure 6:**
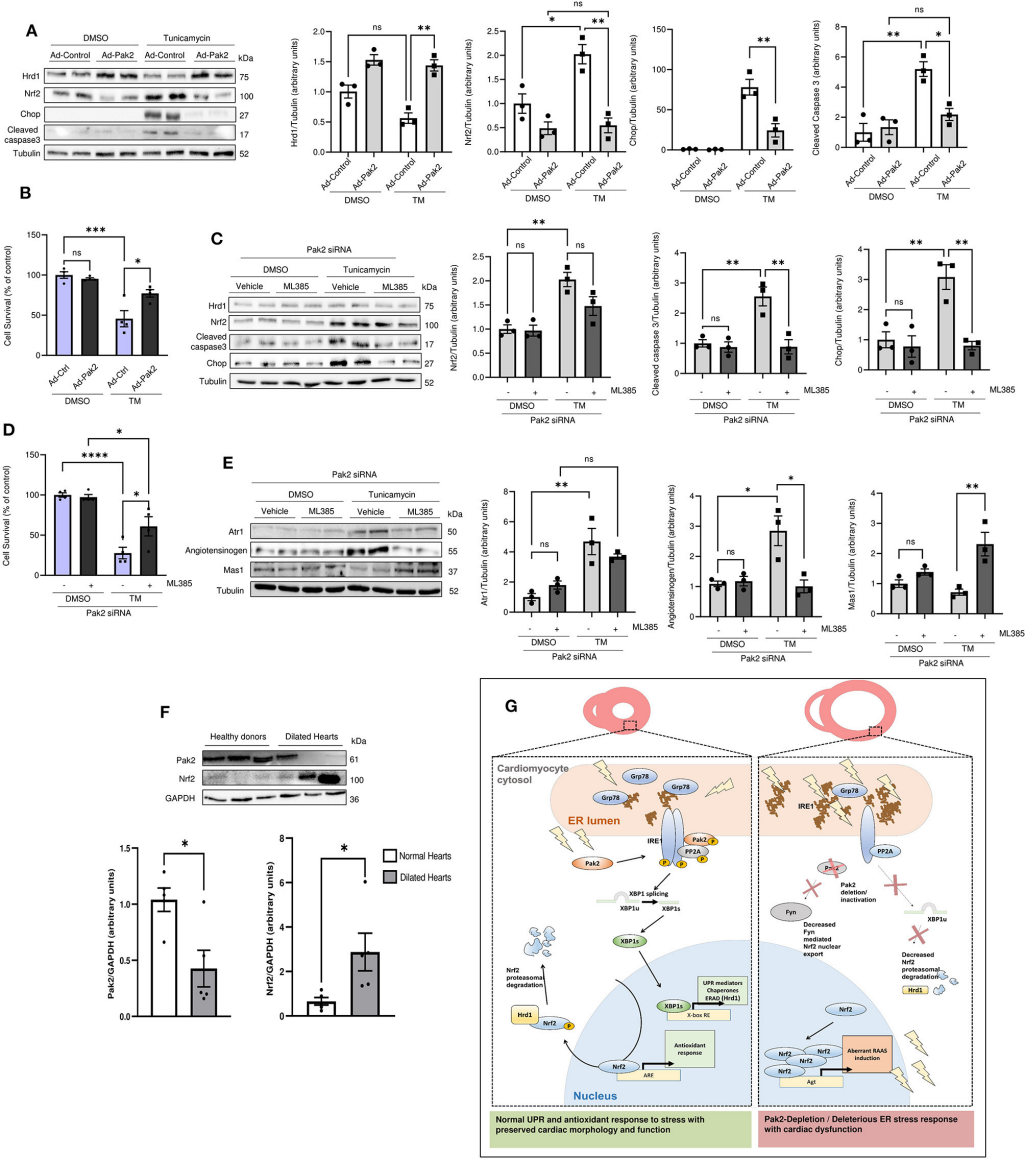
Pak2 activation alleviates ER stress-induced cell death in human cardiomyocites *via* Nrf2 downregulation. **(A)** Immunoblots and quantification of Nrf2, Hrd1 and cell death markers in tunicamycin stressed human-induced pluripotent stem cell-derived cardiomyocytes (iPSC-CMs) infected with adenovirus expressing active Pak2 (Ad-Pak2) (*n* = 3). **(B)** Cell viability of iPSC-CMs was determined by MTT assay (*n* = 4). Immunoblots and quantification of Nrf2, cell death markers **(C)**, ATR1, Angiotensinogen, and Mas1 **(E)** in tunicamycin stressed Pak2 knockdown iPSC-CMs under Nrf2 inhibition with ML385 (*n* = 3). **(D)** The effect on cell viability was determined by MTT assay (*n* = 4). **(F)** Immunoblots and quantification of Pak2 and Nrf2 in heart samples from healthy donors and transplantation patients with dilated heart disease (*n* = 5). Student's *t*-test or 2-way ANOVA with Bonferroni correction for *post-hoc* comparisons were used for analyses. Data presented as mean ± SEM. **(G)** Proposed model for Pak2-mediated regulation of Nrf2 in response to ER stress. *P < 0.05, **P < 0.01, ***P < 0.001, ****P < 0.0001.

Finally, to seek human-relevant data, we tested whether downregulation of Nrf2 *via* Pak2 activation is protective in human cardiomyocytes. Human-induced pluripotent stem cell-derived cardiomyocytes (iPSC-CMs) were subjected to tunicamycin-induced ER stress. Tunicamycin treatment for 24 h resulted in Nrf2 accumulation accompanied by enhanced apoptosis markets (Figure 6A) and a significant reduction in cell survival (Figure 6B). Adenoviral overexpression of active Pak2 was able to restore Hrd1 expression with the subsequent Nrf2 downregulation (Figure 6A), alleviate cell death and enhance cell survival (Figure 6B). Conversely, Pak2 depletion in human cardiomyocytes dramatically increased apoptosis in response to ER stress (Figures 6C,D). Interestingly, these effects observed for Pak2 knockdown on ER stress related cell death can be partially restored by inhibition of Nrf2 (Figures 6C,D) highlighting the detrimental role of Nrf2 in Pak2 defective cardiomyocytes in response to sustained ER stress. Moreover, Pak2 depletion results in altered expression of Atr1 and Mas1 in human cardiomyocytes (Figure 6E) which can be prevented by inhibition of Nrf2. The above data suggest that Pak2 plays a protective role in human cardiomyocytes in response to stress by enhancing the protective UPR and preventing the accumulation of Nrf2 and the consequent aberrant expression of RAAS components leading to cardiomyocytes death. Importantly, these results are consistent with our findings in myocardium from transplantation patients with dilated heart failure showing a significant decrease in Pak2 expression at both protein and transcript level with an increase in Nrf2 protein expression compared to samples obtained from healthy donors (Figure 6F; Supplementary Figure 13). Loss of Pak2 could then contribute to the pathogenesis of heart failure by promoting myocardium damage from defective ER stress response and enhancing Nrf2 maladaptive regulation of RAAS activation.

## Discussion

As a result of cardiac hypertrophy, there is an increased demand for protein synthesis and cellular metabolism, which heavily rely on a prompt UPR and redox response (3, 29). Continued ER stress critically impacts cardiomyocyte function and triggers cardiomyocyte cell death, resulting in tissue damage leading to heart failure (13). Overall, targeting protein quality control to maintain cardiac proteostasis and restore redox balance offers therapeutic benefits to promote cardiac health and alleviate cardiac disease (30).

We had recently described Pak2 as a cardioprotective ER responsive kinase by enhancing the IRE1 branch of the UPR preventing pathological hypertrophy (21). Here we provide evidence for a novel mechanism in which Pak2 contributes to relieve cardiac pathological remodeling. The effects of Pak2 go beyond its role in ER homeostasis, acting as an Nrf2 modulator, functioning as a signaling nexus between the UPR, redox response and RAAS activation. Pak2 depleted hearts under pathological stress, show increased ROS production and altered UPR response. ER stress lead to transcriptional downregulation of genes related to redox metabolism in Pak2 deleted mice. Surprisingly, mice deficient of cardiac Pak2 under sustained ER stress and cardiac dysfunction showed increased expression of Nrf2. Nrf2 is known as a master transcription factor driving antioxidant and detoxifying defense to suppress oxidative stress–mediated cardiac injury (31). Nrf2 deletion leads to cardiac dysfunction at 2 weeks after pressure overload (20). Similarly, Nrf2 deletion exacerbates oxidative stress, fibrosis, and apoptosis in cardiomyocytes from mice following streptozotocin (STZ) induced Type I diabetes (32). Conversely, pharmacological activation and cardiomyocyte specific Nrf2 overexpression alleviates myocardial stress, suppresses necrosis and enhances the clearance of accumulated ubiquitinated proteins, after pressure overload in mice (33). Thus, Nrf2 has been proposed an attractive therapeutic target for cardiovascular disease. However, restoration or pharmacological activation of Nrf2 has not been a successful therapeutic intervention (34). Moreover, there is evidence demonstrating a detrimental role of Nrf2 in the heart. In a mouse model of myocardial ischemia-reperfusion, Nrf2 knockout prevents cardiac injury *via* increasing nitric oxide (NO) production (35). In transgenic mice expressing a human missense mutant of alpha B-crystallin (hCryAB) in cardiomyocytes, persistent Nrf2 activation leads to excessive antioxidant gene expression resulting in reductive stress, accumulation of ubiquitinated proteins and pathological hypertrophy (36). Moreover, in mice with compromised autophagy, Nrf2 knockout has been shown to alleviate cardiac dysfunction by preventing Nrf2 mediated *Agt* upregulation in response to pressure overload (20). Thus, understanding the mediators for Nrf2 functional dichotomy in the heart is key before considering the use of Nrf2 as a therapeutic target. Although the precise mechanisms entailing Nrf2-mediated cardiac damage are unclear, proteostasis disruption may be critical for Nrf2 switching into a maladaptive role.

In the current study, Nrf2 expression was increased in hearts subjected to sustained ER stress and this was exacerbated by Pak2 deletion. Interestingly, Pak2 depleted cardiomyocytes, excessive Nrf2 nuclear accumulation under tunicamycin stress did not seem to intensify antioxidant gene expression. Therefore, the induction of reductive stress is an unlikely mechanism for the detrimental effects of Nrf2 in Pak2 deleted mice. However, in Pak2 deleted cardiomyocytes under ER stress, there was a significant induction of RAAS genes, highly implicated in the pathological process of cardiovascular diseases (37). Angiotensin II is the major effector *via* activation of angiotensin II receptors which initiate downstream pathways leading to apoptosis, cardiac hypertrophy and fibrotic remodeling (38), whereas Angiotensin-(1–7), and Mas1 activation contribute to cardioprotection (39). Importantly, dysregulation of RAAS genes has been shown to induce ROS production *via* NADPH oxidase activation, and increased protein aggregation (40), and RAAS inhibition has been shown to diminish oxidative stress in several pathological conditions (41). Importantly, Nrf2 has been previously shown to regulate angiotensinogen expression by binding directly to *Agt* and *Ace1* gene promoter (42), suggesting a role for Nrf2 on their transcriptional regulation. Nrf2 mediated RAAS gene expression has also been shown to contribute to diabetic nephropathy. Nrf2 deletion or inhibition is able to upregulate *Ace2/MasR* while downregulating *Agt/Ace* expression alleviating hypertension and kidney injury (19). Consistent with this, our data shows that Pak2 deletion under ER stress leads to a shift from the antioxidant program with early activation of *Nqo1, Ho1*, and *G6pd* to an increase in *Agt, Ace1*, and *Atr1* expression with partial *Mas1* downregulation in a Nrf2 dependent fashion. Furthermore, we observed that the increased cell death in Pak2 depleted cardiomyocytes under ER stress can be alleviated by administration of the anti-hypertensive drugs Losartan and Enalapril. Thus, local Angiotensin II induced ROS could thus explain why increased Nrf2 expression was not able to attenuate oxidative stress in hearts from mice under tunicamycin stress or pressure overload. We propose that Pak2 and UPR dysregulation leading to defective cardiomyocyte proteostasis is a contributing factor for Nrf2 switching to aberrant activation leading to pathological remodeling in response to ER stress. The observation that Pak2 deletion-induced cardiomyocyte death is alleviated by Nrf2 inhibition under ER stress suggests a notion that Pak2 cardioprotective effect is not solely due to its role in maintaining ER homeostasis. Overall, we show that Pak2 mediated cardiac protection in response to ER stress and oxidative stress can be attributed to enhancing UPR and alleviation of aberrant Nrf2 activity. Further investigation of molecular mechanisms responsible for Nrf2-mediated myocardial damage, such as precise events promoting Nrf2 maladaptive transcriptional activity will lead to a better understanding of Nrf2 dichotomy in the pathogenesis of cardiomyopathies toward heart failure.

We have previously demonstrated that Pak2 stimulates the activation of transcriptional factor XBP1s, which upregulates ERAD components, including the E3 ubiquitin ligase Hrd1 to enhance misfolded proteins clearance (21). ERAD is an essential component of the adaptive ER stress response for preserving heart structure and function (43). We have found that Pak2 mediates Nrf2 downregulation by increasing its degradation *via* Hrd1 dependent ubiquitination. Of note, negative regulation of Nrf2 by the XBP1–Hrd1 arm of UPR has been observed in cirrhotic liver models resulting in increased ROS (17). Similarly, XBP1 *via* Hrd1 has been shown to modulate the antioxidant function of Nrf2 in ischemia-reperfusion induced acute kidney injury (44). In these models, Hrd1 mediated Nrf2 inhibition leads to impaired antioxidant response and detrimental increase in ROS. However, expression of XBP1 or Hrd1 is able to prevent cardiac remodeling in hearts of mice subjected to ischemia-reperfusion injury and pressure overload (43, 45). In line with this, we demonstrated that XBP1s cardiac overexpression by AAV9 injection could effectively recover Hrd1 expression and downregulate Nrf2 accumulation in Pak2-CKO mice halting pathological progression in tunicamycin stressed hearts. Furthermore, Hrd1 inhibition was able to revert the Pak2 mediated Nrf2 downregulation in cardiomyocytes, indicating that Pak2/IRE-1/XBP1s/Hrd1 signaling cascade is responsible for maintenance of optimal expression of Nrf2. The crosslink between the antioxidant response and the UPR has been previously observed. In response to mitochondrial stress, Nrf2 interacts with ATF4 to upregulate antioxidants and antiapoptotic genes (46). Moreover, PERK has been shown to directly phosphorylate Nrf2 leading to its nuclear translocation and activation (47). Interestingly, XBP1s has been shown to be a Nrf2 transcriptional activator (48). Decreased XBP1 activity in Pak2 depleted mice might then account for the initial loss of Nrf2 under acute ER stress. However, the exact mechanism by which Pak2 knockout initially deceases Nrf2 expression that remains unknown.

Therapeutically, this study provides evidence showing that active AAV9-mediated Pak2 delivery in the heart inhibits hypertrophy and cardiac dysfunction induced by tunicamycin. Importantly, we have shown Pak2 mediated downregulation of Nrf2 attenuates apoptosis during sustained ER stress *in vitro* and *in vivo*. Our findings highlight that Pak2-dependent proteostasis plays a critical role in controlling aberrant Nrf2 induction of RAAS activation, widening our understanding of the Nrf2 controversial actions in the heart. These results support previous reports demonstrating that Nrf2 inhibition prevents apoptosis in cells presenting impaired autophagy, further highlighting the importance of maintenance of proteostasis in Nrf2 dependent maladaptive responses. Furthermore, these findings might explain why sole antioxidant therapy or Nrf2 activation have not been effective for reverting the outcome of cardiac disease. Importantly, above described Pak2-regulated cardioprotective effects were verified in human cardiomyocytes. In human iPSC-CMs, active Pak2 expression enhances Hrd1 and downregulate Nrf2 expression preventing cell death. Moreover, Nrf2 inhibition could alleviate Pak2 deletion induced cell death in tunicamycin treated iPSC-CMs. Consistent with this, a significant reduction of Pak2 and increased Nrf2 were observed in myocardium from patients with dilated heart failure. To our knowledge, the current study is the first report indicating Pak2 as an upstream regulator inhibiting Nrf2 aberrant activation. The human relevance of this regulatory mechanism forms a translational grounding to propose a therapeutic role for Pak2 in relieving stress triggered cardiac dysfunction.

The current study has several limitations. Although binding sites for Nrf2 in RAAS genes promoter regions are conserved among rats, mice and humans, direct evaluation of Nrf2 binding site occupation in response to ER stress and the effect on their respective transcript expression in a human setting such as iPSC derived cardiomyocytes or human tissue remains to be tested. Moreover, the precise events involved in Nrf2 shift from its redox activity toward maladaptive RAAS expression and the effect of Pak2 in Nrf2 mediated transcription need to be considered. Furthermore, the spatial regulation of Nrf2 needs to be assessed, whether Nrf2 nuclear export is facilitated by Pak2 activity leading to its availability for ubiquitination by Hrd1 and subsequent degradation remains to be elucidated. Additionally, further investigations focusing on the pharmacological modulation of Nrf2 in an Pak2 deficient or ER stress context *in vivo* would highly contribute to the translational potential of our findings. Finally, whether the maladaptive role of Nrf2 in hearts under sustained ER stress or Pak2 deficient mice is restricted to pressure overload and tunicamycin induced stress mice is unclear. The importance of Pak2 in Nrf2 modulation on cardiac remodeling and dysfunction in other pathological settings, such as myocardial infarction and hypertensive cardiac hypertrophy would further contribute to our understanding of Nrf2 mediated cardiac maladaptive remodeling in ER stressed hearts and its role in the pathogenesis of cardiomyopathies toward heart failure.

## Conclusions

We identified Pak2 as a novel signaling nexus between the UPR and the redox response with a protective role in the heart. We have shown that even though Nrf2 activation is cardioprotective during initial stress, sustained ER stress and defective proteostasis in Pak2 deleted hearts renders Nrf2 detrimental. Excessive Nrf2 nuclear accumulation promotes abnormal RAAS gene expression in the heart leading to increased cardiomyocyte death and cardiac dysfunction. Under sustained ER stress, Pak2 acts as a negative regulator of Nrf2 *via* mediated XBP1-Hrd1 mediated Nrf2 ubiquitination and degradation (Figure 6G). Modulating Pak2 activation is thus proposed as a therapeutic strategy to ameliorate ER dysfunction and aberrant RAAS activation, major pathological determinants underlying many forms of heart disease in humans.

## Data Availability Statement

The original contributions presented in the study are included in the article/Supplementary Materials, further inquiries can be directed to the corresponding author/s.

## Ethics Statement

The study complies with the Declaration of Helsinki and was reviewed and approved by the Ethics Committee of the Technische Universität Dresden (Az.: EK 422092019 and EK EK 446122011). The patients/participants provided their written informed consent to participate in this study. Animal studies were performed in accordance with the United Kingdom Animals (Scientific Procedures) Act 1986 and were approved by the University of Manchester Ethics Committee.

## Author Contributions

PB designed and carried out experiments, analyzed and interpreted data, and wrote the manuscript. BN and LC carried out experiments and acquired data. MZ performed surgical procedures. WL performed and analyzed gene array data. FC performed bioinformatic analyses. XL and KG generated and cultured hiPSC-CMs. SH, NF, and OM produced AAV9 viruses. EC provided experimental advice and technical support. JC generated and provided Pak2fl/fl mouse line. XW conceptualized the study and experiments and interpreted results. All authors contributed to the article and approved the submitted version.

## Funding

This work was supported by the British Heart Foundation (PG/17/31/32988, and PG/14/70/31039 to XW).

## Conflict of Interest

The authors declare that the research was conducted in the absence of any commercial or financial relationships that could be construed as a potential conflict of interest.

## Publisher's Note

All claims expressed in this article are solely those of the authors and do not necessarily represent those of their affiliated organizations, or those of the publisher, the editors and the reviewers. Any product that may be evaluated in this article, or claim that may be made by its manufacturer, is not guaranteed or endorsed by the publisher.
