# Supplementary material for: Pak2 Regulation of Nrf2 Serves as a Novel Signaling Nexus Linking ER Stress Response and Oxidative Stress in the Heart
Source: Front Cardiovasc Med. 2022 Mar 8;9:851419. doi: 10.3389/fcvm.2022.851419 (PMC8957820; doi:10.3389/fcvm.2022.851419)
Supplement: Supplementary file 1 [file Data_Sheet_1.PDF]

Supplementary Methods

Adult rat cardiomyocytes (ARCMs) isolation and culture

Six weeks old, male, Wistar rats (150g) were sacrificed by intraperitoneal injection of 750µl Pentobarbitone Sodium 20% w/v (Pentoject) and 250µl Heparin Sodium (1000 U/ml, Wockhardt UK). The heart were excised and placed in an ice-cold perfusion buffer containing heparin (Hank's balanced salt solution, HBSS, Sigma), 10mM Butandione monoxime, 11mM glucose, 1mM $MgSO_4$; 10% heparin). Next, the heart was cannulated and mounted on a Langendorff perfusion apparatus and perfused with calcium-free digestion buffer containing Collagenase type II (65 U/ml, Worthington Biochemical) and protease from Streptomyces Griseus (0.28 U/ml, Sigma) for 20 minutes. After perfusion, the heart was carefully transferred into trypsin solution (4.4 U/ml Trypsin, Worthington; 70 U/ml DNase I, Worthington) and sliced into small pieces. Digestion buffer (115U/ml Collagenase type II, Worthington; 0.14 U/ml Protease from Streptomyces Griseus (Sigma), 4.4 U/ml Trypsin, Worthington and 70 U/ml DNase I, Worthington in perfusion buffer) was added and incubated in a 37°C water bath for 20 minutes. Cell suspensions were passed through a 100µm filter (Corning). The filtered cell suspensions were centrifuged at 1200rpm for 3 minutes and the cell pellet was washed twice with wash buffer (1:1 perfusion buffer and ACCT media consisting in low glucose, GlutaMAX™, pyruvate, no HEPES DMEM, 1g BSA, 5mM L-carnitine, 2mM Creatine, 5mM Taurine and 100U/ml penicillin/1mg/ml streptomycin). Afterwards, the cell suspension was slowly pipetted on top of a BSA solution (1mM BSA in ACCT media) and left to pass through the gradient for 40 minutes to remove dead cells and non-cardiomyocytes. The resultant cells were resuspended in ACCT media containing blebbistatin (20µM, Abcam) and plated on plates coated with Geltrex (Gibco, LDEV-Free, hESC-qualified Reduced Growth Factor Basement Membrane).

Transfection of H9C2, ARCMs and iPSC-derived cardiomyocytes

2 µg cDNA plasmids were transfected in H9C2 or ARCMs using Lipofectamine 2000 reagent (Invitrogen) to investigate Pak2, XBP1 and Hrd1 overexpresison, for HA-ubiquitin immunoprecipitation and luciferase reporter activity, while siRNAs (100 nM) were transfected in iPS-CMs using Lipofectamine Plus reagent (Invitrogen) according to the manufacturers' instructions. Pak2 siRNA (gene ID #5062, si genome SMART pool, 100 nM, Dharmacon),

Hrd1 siRNA (siRNA ID:s167864, 4390771 ThermoFisher). Negative control siRNA (siScr, AGGUAGUGUAAUCGCCUUG) was obtained from Sigma.

Construction and administration of adeno-associated virus.

The pSSV9-TnT-eGFP plasmid was modified by replacing GFP with Flag-tagged constitutively active Pak2(T402E) cDNA or Flag-tagged spliced XBP1 (XBP1s) cDNA. To obtain the recombinant AAV9 virus, HEK293T cells were co-transfected with an adenoviral helper plasmid (pDGΔVP), a pAAV2-9 Rep-Cap plasmid (p5E18-VD2/9) and an adeno-associated virus genome plasmids pSSV9-TnT-Pak2, pSSV9-TnT-eGFP or pSSV9-TnT-XBP1s. AAV9 viruses were purified and characterized as previously described[21]. Mice were anesthetized with 2% isoflurane and 1×10^{11} genomic particles were administered by tail vein injection.

Adenoviral production and infection

SureSilencing shRNA plasmids were purchase from Qiagen, the negative control rat shRNA (GGAATCTCATTCGATGCATAC) and rat shPak2 (GGAGTTAAAGAATCCCAACAT) plasmids were generated and packed into adenovirus (Ad-shCon and Ad-shPak2) using the BLOCK-iT adenoviral RNAi expression system (Invitrogen). For adenoviral mediated Pak2 overexpression constitutively active (T402E) Pak2 cDNA or GFP cDNA was cloned into the pAd/CMV/V5-DEST™ gateway vector (Invitrogen) (Ad-control and Ad-Pak2). Virus amplification and purification was performed as previously described[22.] Adenovirus infection was applied at 25 multiplicity of infection (MOI).

Non-invasive blood pressure measurement

The CODA 2-Channel Non-Invasive Blood Pressure system was used to measure blood pressure of conscious mice. The mice were acclimated to restraint and to tail-cuff inflation a day before the actual experiment. The restraint platform was maintained at approximately 33-34°C. The procedure used was followed via the manufacture's instruction manual.

Masson's Trichrome Staining

5µm-thick paraffin embedded heart sections were fixed in Bouin's solution for two hours at room temperature following staining in Harris' haematoxylin for 5 minutes. Sections were washed in water and stained in Red solution (90ml 1%(w/v) Biebrich Scarlet, 10ml 1%(w/v)

Acid Fuchsin, 1ml Glacial Acetic acid) and then treated for 15 minutes in 2.5%(w/v) phosphomolybdic acid before being stained with aniline blue (Sigma) for 3 minutes. Slides were rinsed briefly before being treated in 1% acetic acid for 1 minute. Slides were subsequently dehydrated and cleared. Coverslips were mounted using Eukitt quick-hardening mounting medium (Sigma-Aldrich). The images were obtained by Zeiss Axioplan2 microscope and then analyzed by Image J software. Percentage of tissue area stained blue, was calculated and expressed as percentage of interstitial fibrosis.

Prediction of Nrf2 binding sites

Bioinformatics analysis was carried out to investigate potential Nrf2 binding sites. The Eukaryotic Promoter Database (epfl.ch) was used to retrieve the promoter sequences (ranging from - 1500kb to 100kb) of the relevant genes in humans, rats and mice. The promoter sequences for each species were analyzed using TFBIND (http://tfbind.hgc.jp/) and LASAGNA search 2.0, to search for Nrf2 binding sites. Hits with a cut-off p-value <0.005 or <0.001 were selected. TFBS prediction in regions with over 70% identity across the three species are represented in the promoter.

Fractional protein extraction

Fractional proteins were extracted with gradient centrifugation from H9C2 cells. Briefly, samples were lysed in cytoplasmic buffer (Tris 20 mM, NaCl 10 mM, $MgCl_2$ 2 mM, EDTA 2 mM, β-glycerophosphate 25 mM, Na_3VO_4 1 mM, PMSF 1 mM, Aprotinin 1.54 μM, and Leupeptin 21.6 μM; pH 7.4). The lysates were spun at 2,000g for 10 minutes in order to remove cytoplasmic proteins. The pellets were re-suspended in nuclear buffer (Tris 50 mM, NaCl 500 mM, EDTA 2 mM, 10% Glycerol, 1% TritonX-100, β-glycerophosphate 25 mM, Na_3VO_4 1 mM, PMSF 1 mM, Aprotinin 1.54 μM, and Leupeptin 21.6 μM; pH 7.4), followed by centrifugation at 10,000g for 10 minutes, leaving the nuclear proteins suspended in the supernatant. The cytoplasmic portion was spun at 100,000g for 2 hours. The supernatant was collected as cytosolic protein portion. Protein concentration was determined by Bradford assay (Bio-Rad). The antibody against H3 (Cell Signalling, 9715) was used as nuclear marker.

Affymetrix Gene Expression Array

Total RNA was extracted from heart tissue using Trizol reagent (Invitrogen). RNA quality was checked using an Agilent 2100 Bioanalyser (Agilent Technologies). Hybridization cocktail was hybridized to Mouse Genome 430 2.0 arrays (Affymetrix) according to manufacturer's instructions. Arrays were read using Agilent GeneArray scanner 3000 7G using Affymetrix GCOS (V1.4) software. Quality control and outlier analysis was performed with dChip (V2005) using the default settings. Background correction, normalization and gene expression analysis were performed using RMA in Bioconductor(1). Differential expression analysis between the two groups (Flox and CKO) was performed as one-way test with Limma using the functions lmFit and eBayes(2). Gene lists of differentially expressed genes were controlled for false discovery rate errors using the method of QVALUE(3).An expression diagram of selected genes was generated by normalizing to Pak2-Flox group treated with tunicamycin (in log scale the mean of Pak2-Flox group was subtracted from each expression value).

1. Bolstad BM, Irizarry RA, Astrand M, Speed TP. A comparison of normalization methods for high density oligonucleotide array data based on variance and bias. Bioinformatics. 2003; 119:185-193.

2. Smyth, GK. Linear models and empirical Bayes for assessing differential expression in microarray experiments. Stat Appl Genet Mol Biol. 2004; 3: Article 1.

3. Storey JD, Tibshirani R. Statistical significance for genomewide studies. Proc Natl Acad Sci USA. 2003; 100:9440-9445.

Public Data Access

The human RNA expression data from Left Ventricle Biopsy discussed in this publication have been deposited in NCBI's Gene Expression Omnibus (Edgar et al., 2002) and are accessible through GEO Series accession number GSE1145 (https://www.ncbi.nlm.nih.gov/geo/query/acc.cgi?acc=GSE1145). This data has been made public March, 2004.

Edgar R, Domrachev M, Lash AE. Gene Expression Omnibus: NCBI gene expression and hybridization array data repository. Nucleic Acids Res. 2002 Jan 1;30(1):207-10. doi: 10.1093/nar/30.1.207. PMID: 11752295; PMCID: PMC99122.

Data Availability

The data underlying this article will be shared on reasonable request to the corresponding author.

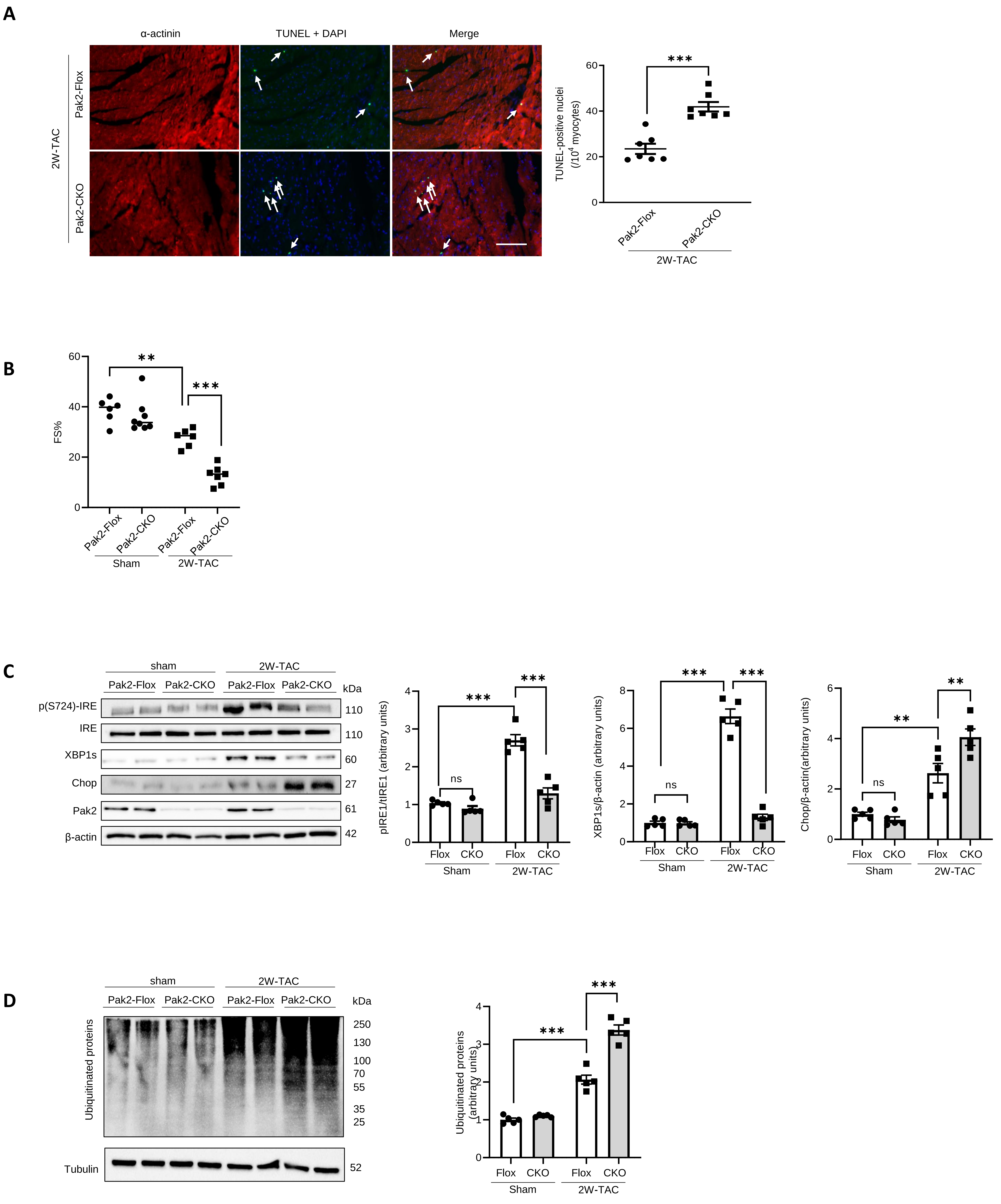

Suplementary Figure I: Pak2 cardiac deletion enhances ER stress induced cardiac dysfunction and defective UPR. (A) TdT-mediated dUTP nick end labeling (TUNEL) assay of the hearts after TAC (scale bar=20 μm), arrows indicate TUNEL positive nuclei (n=6–8). **(B)** Echocardiographic analysis of Pak2 cardiac-specific knockout (Pak2-CKO) mice after transverse aortic constriction (TAC) (n=6–8). Detailed Echocardiographic data available in Supplementary Table II. **(C)** Immunoblots and quantification of total ubiquitinated proteins in TAC-stressed hearts (n=5). **(D)** Immunoblots and quantification of total ubiquitinated proteins in TAC-stressed hearts (n=5). 2-way ANOVA with Bonferroni correction for post hoc comparisons were used for analyses. Data presented as mean±SEM.

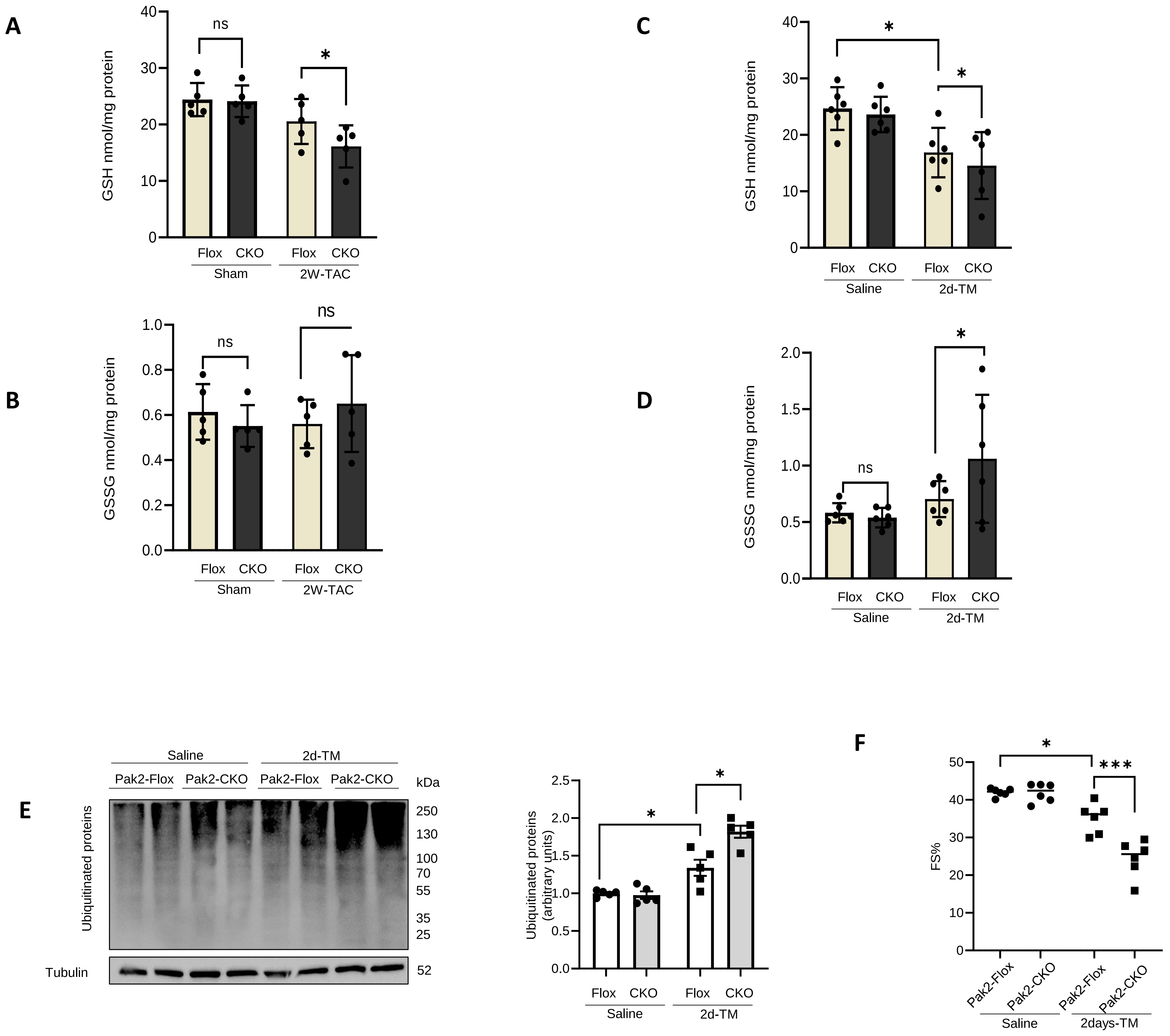

Suplementary Figure II: Pak2 cardiac deletion enhances ubiquitinated protein accumulation and oxidative stress in the heart. Oxidative stress was assessed by measuring the content of reduced **(A)** and oxidised **(B)** glutathione in TAC-stressed hearts (n=5). Oxidative stress was assessed by measuring the content of reduced **(C)** and oxidised **(D)** glutathione in tunicamycin-stressed hearts (n=6). **(E)** Immunoblots and quantification of total ubiquitinated proteins in tunicamycin-stressed hearts (n=5). **(F)** Echocardiographic analysis of Pak2-CKO mice after 2 days of Tunicamycin (TM, 2 mg/kg) injection (n=6). 2-way ANOVA with Bonferroni correction for post hoc comparisons were used for analyses. Data presented as mean±SEM.

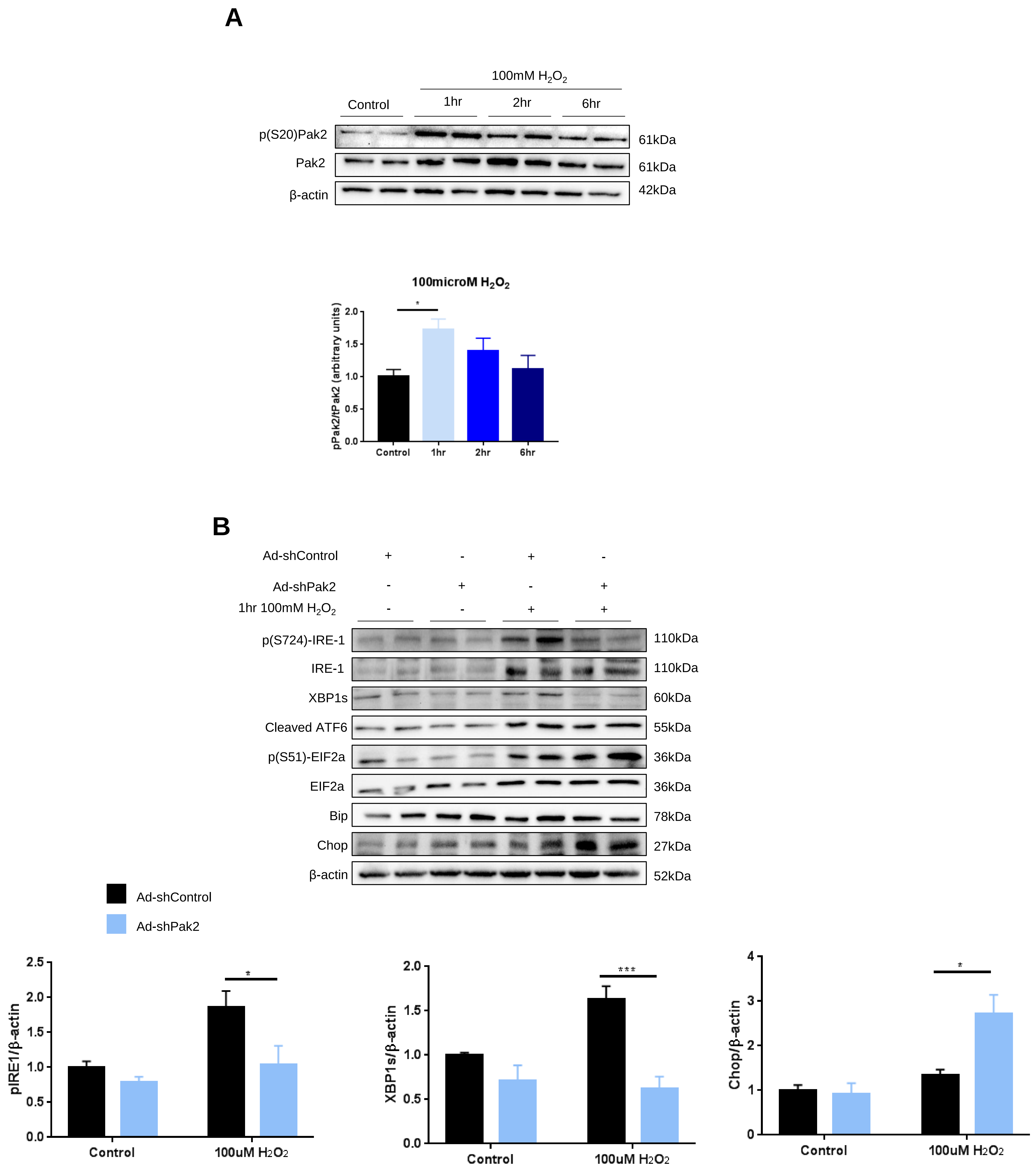

Supplementary Figure III: Pak2 is a redox responsive kinase in cardiomyocytes. (A) Immunoblots and quantification of phosphorylated Pak2 and total Pak2 from ARCMs subjected hydrogen peroxide incubation for 1, 2 and 6 hours (n=4). **(B)** Immunoblots and quantification of UPR components in Pak2 depleted (Ad-shPak2) ARCMs under oxidative stress (n=4). 1-way or 2-way ANOVA with Bonferroni correction for post hoc comparisons were used for analyses. Data presented as mean±SEM.

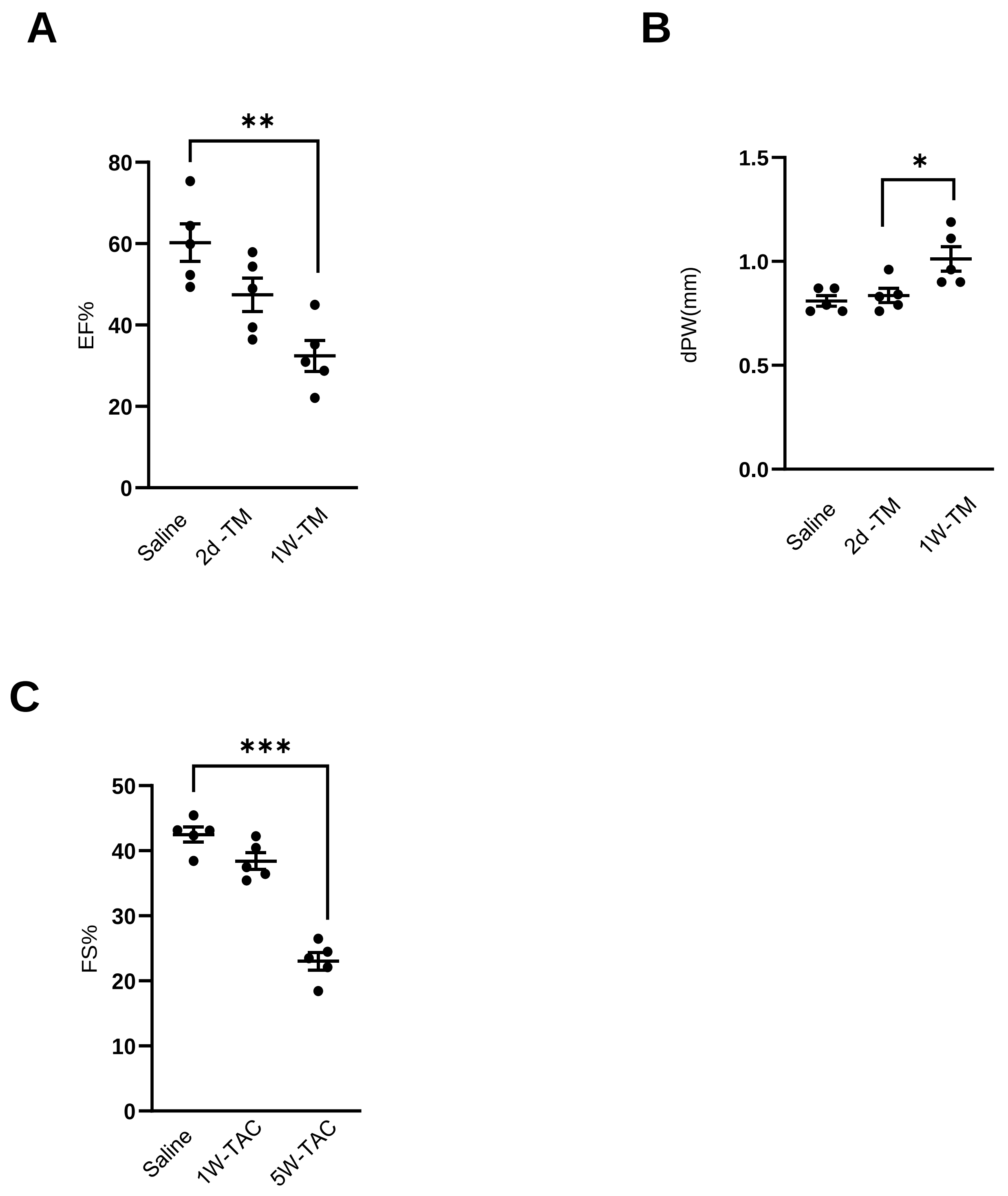

Supplementary Figure IV: ER stress promotes cardiac dysfunction in mice. Echocardiographic analysis **(A)** and **(B)** of C57BL/6N mice after tunicamycin injection for 2 days and 1 week (n=5). **(C)** Echocardiographic analysis of C57BL/6N mice after TAC for 1 and 5 weeks (n=5). Detailed Echocardiographic data available in Supplementary Table III 1-way ANOVA with Bonferroni correction for post hoc comparisons were used for analyses. Data presented as mean±SEM.

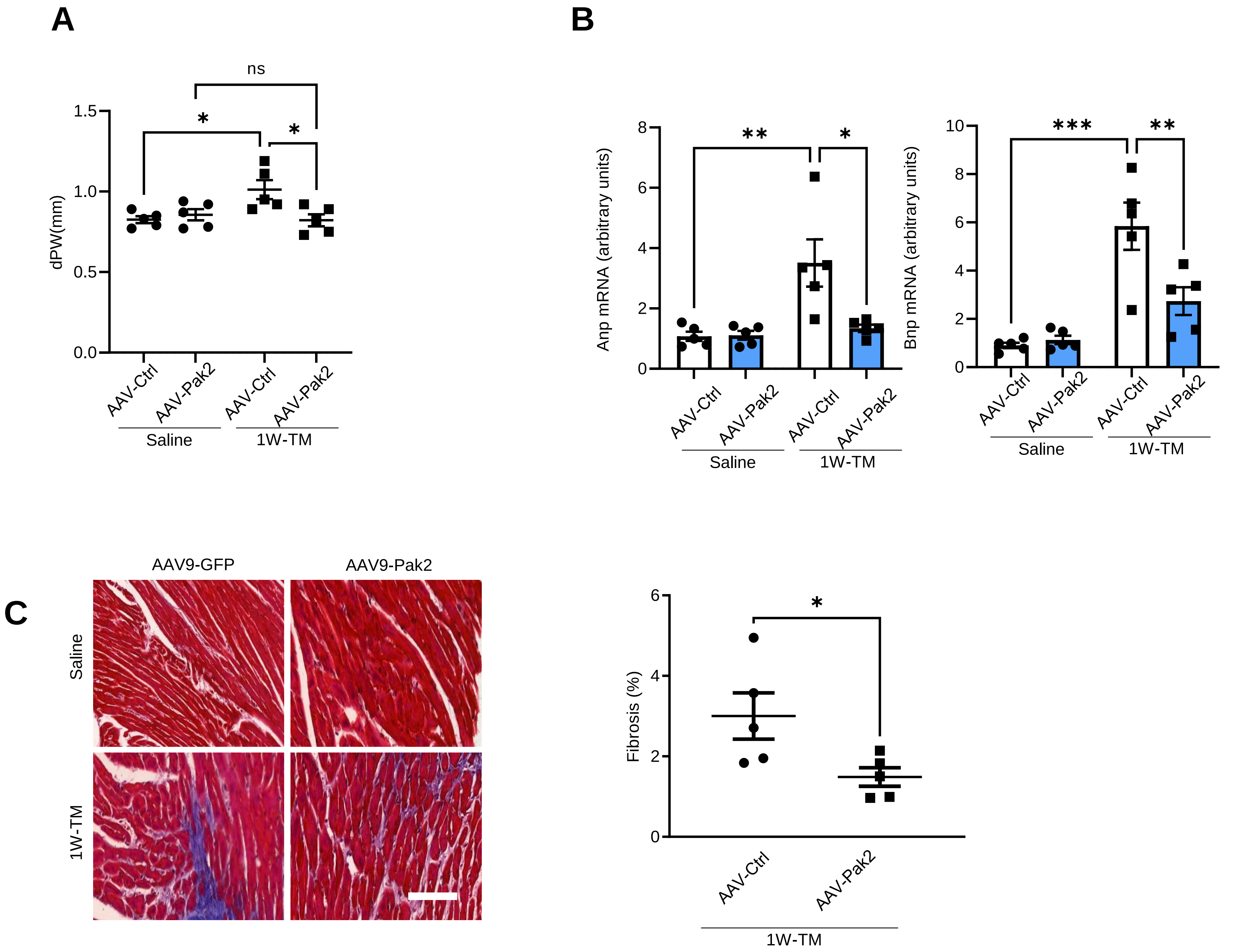

Supplementary Figure V: Activated Pak2 prevents cardiac hypertrophy and fibrosis in hearts under sustained ER stress. (A) dPW determined by echocardiographic measurement and **(B)** qPCR analyses of the hypertrophic markers *ANP* and *BNP* showed decreased cardiac hypertrophy in AAV-Pak2 compared to AAV-Crtl mice following 1 week Tunicamycin injection (n=5). Detailed Echocardiographic data available in Supplementary Table III. **(C)** Masson's trichrome staining analysis showed lower fibrosis in AAV-Pak2 injected hearts compared to AAV-Crtl (n=5, scale bar=20μm). 2-way ANOVA with Bonferroni correction for post hoc comparisons were used for analyses. Data presented as mean±SEM.

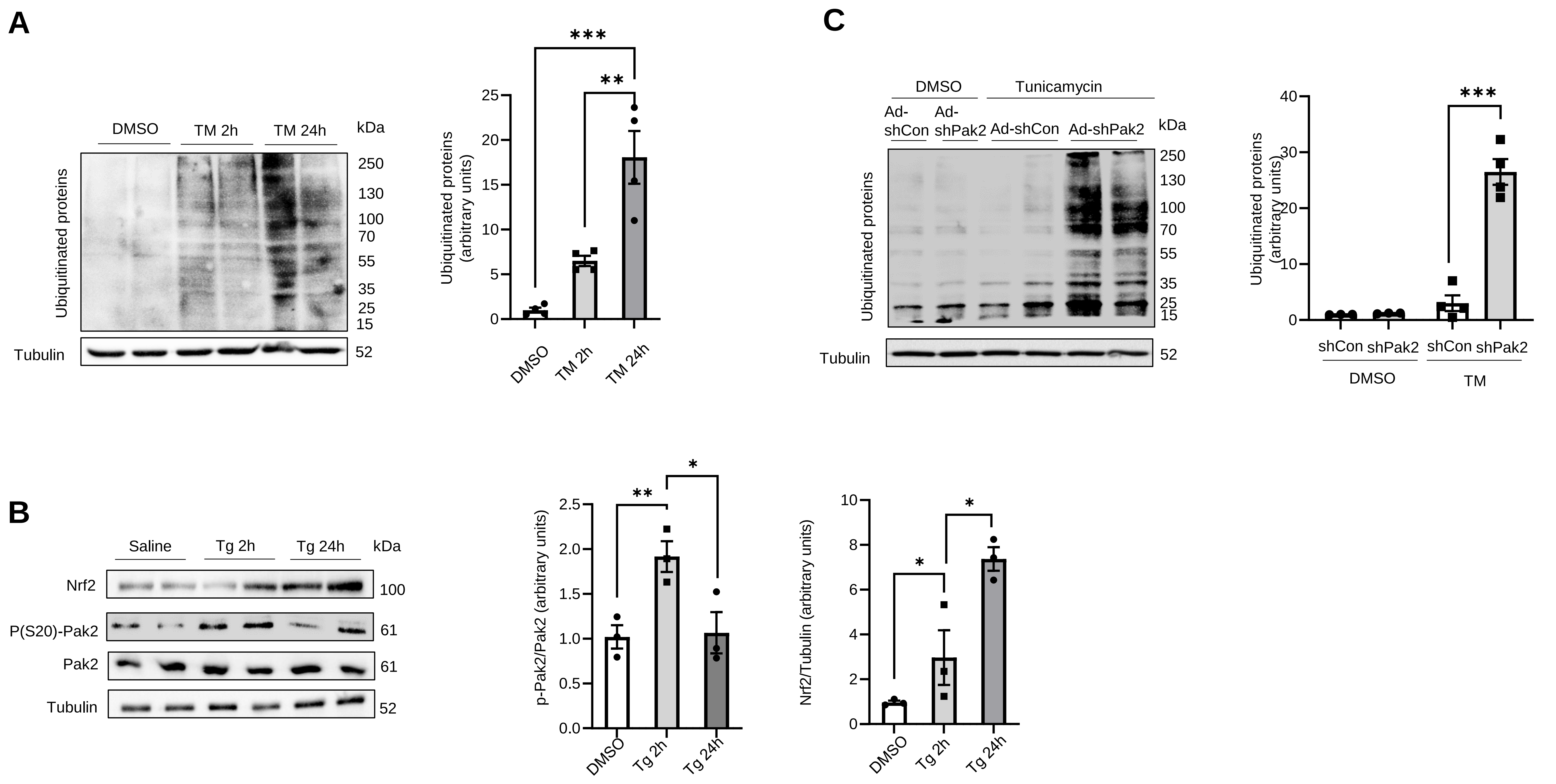

Supplementary Figure VI: Sustained ER stress and Pak2 deletion promote ubiquitinated protein accumulation in cardiomyocytes. (A) Immunoblots and quantification of total ubiquitinated proteins in Tunicamycin treated ARCMs (n=4). **(B)** Immunoblots and quantification of phosphorylated Pak2 and Nrf2 in ARCMs subjected to Thapsigargin (Tg, 0.5 µM)-induced ER stress (n=3). **(C)** Immunoblots and quantification of total ubiquitinated proteins of Pak2-knockdown (Ad-shPak2) ARCMs under tunicamycin (TM) treatment (n=3-4). 1-way or 2-way ANOVA with Bonferroni correction for post hoc comparisons were used for analyses. Data presented as mean±SEM.

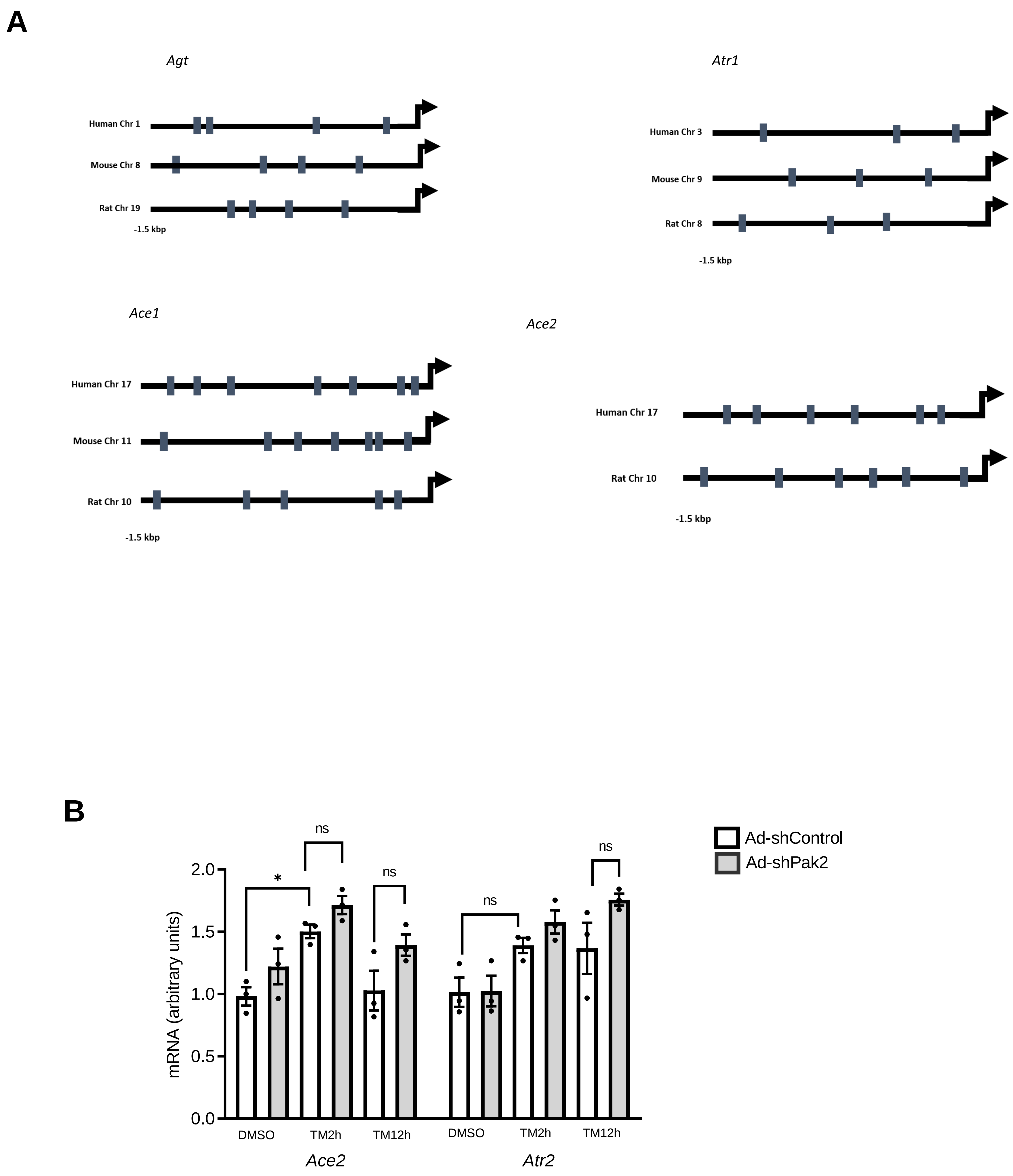

Supplementary Figure VII: Key RAAS component are transcriptional targets for Nrf2 in Cardiomyocytes. (A) Schematic figure showing Nrf2 consensus binding sites in the promoter region (-1.5Kb from transcription start site) of *Agt*, *Atr1*, *Ace1*, and *Ace2* gene conserved in human, rat, and mouse. **(B)** qPCR analysis of *Ace2* and *Atr2* transcript expression in Ad-shPak2 H9C2 cells under ER stress (n=3). 2-way ANOVA with Bonferroni correction for post hoc comparisons were used for analyses. Data presented as mean±SEM.

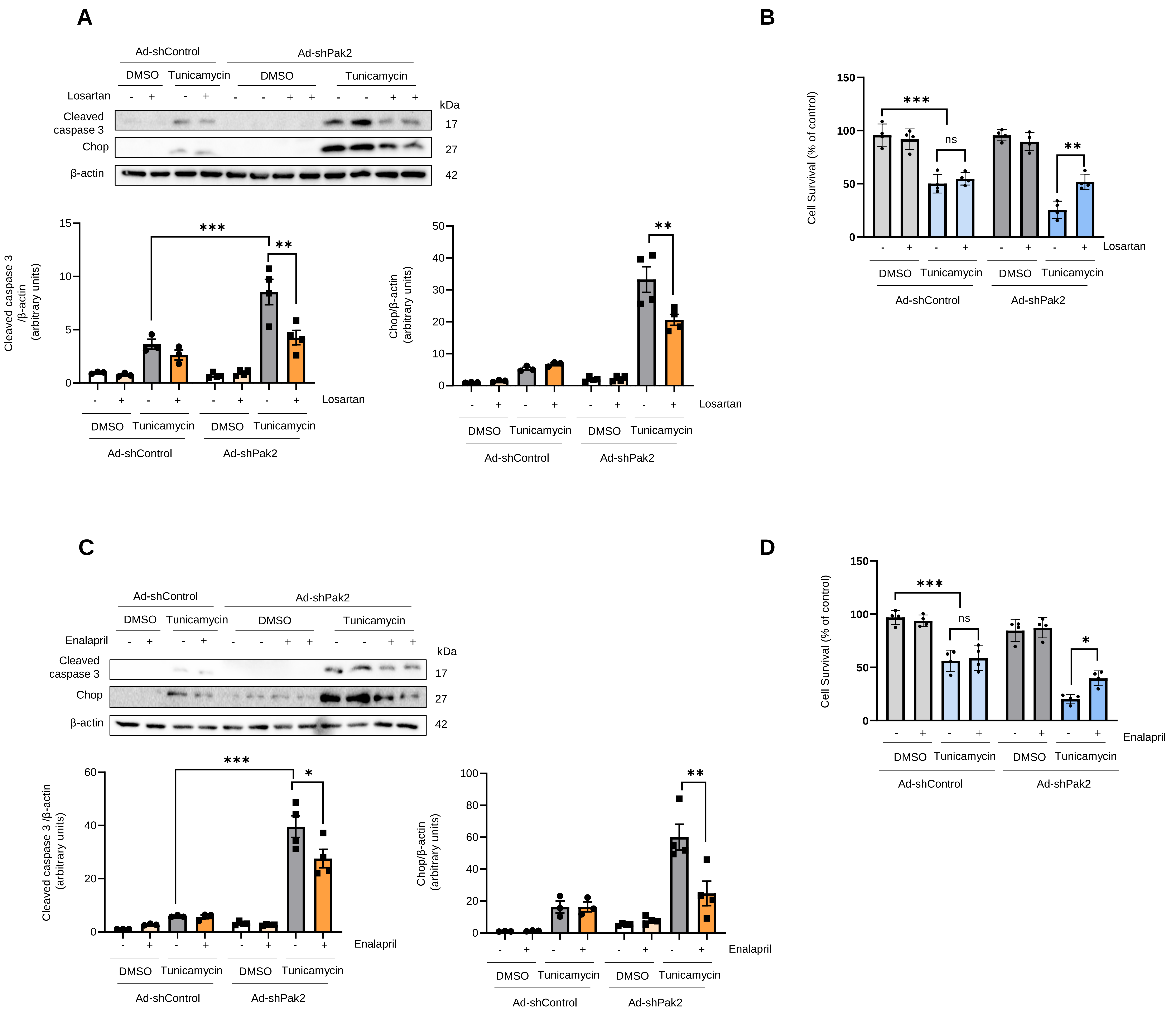

Supplementary Figure VIII: (A) Immunoblots of apoptotic markers in Pak2-knockdown (Ad-shPak2) ARCMs under TM stress and treated with the Angiotensin II receptor blocker Losartan (10µM) (n=3-4). **(B)** The effect on cell viability was determined by MTT assay (n=4). **(C)** Immunoblots of apoptotic markers in Pak2-knockdown (Ad-shPak2) ARCMs under Tunicamycin (TM) stress and treated with the ACE inhibitor Enalapril (20µM) (n=3-4). **(D)** The effect on cell viability was determined by MTT assay (n=4). 2-way ANOVA with Bonferroni correction for post hoc comparisons were used for analyses. Data presented as mean±SEM. *P<0.05, **P<0.01, **P<0.001.

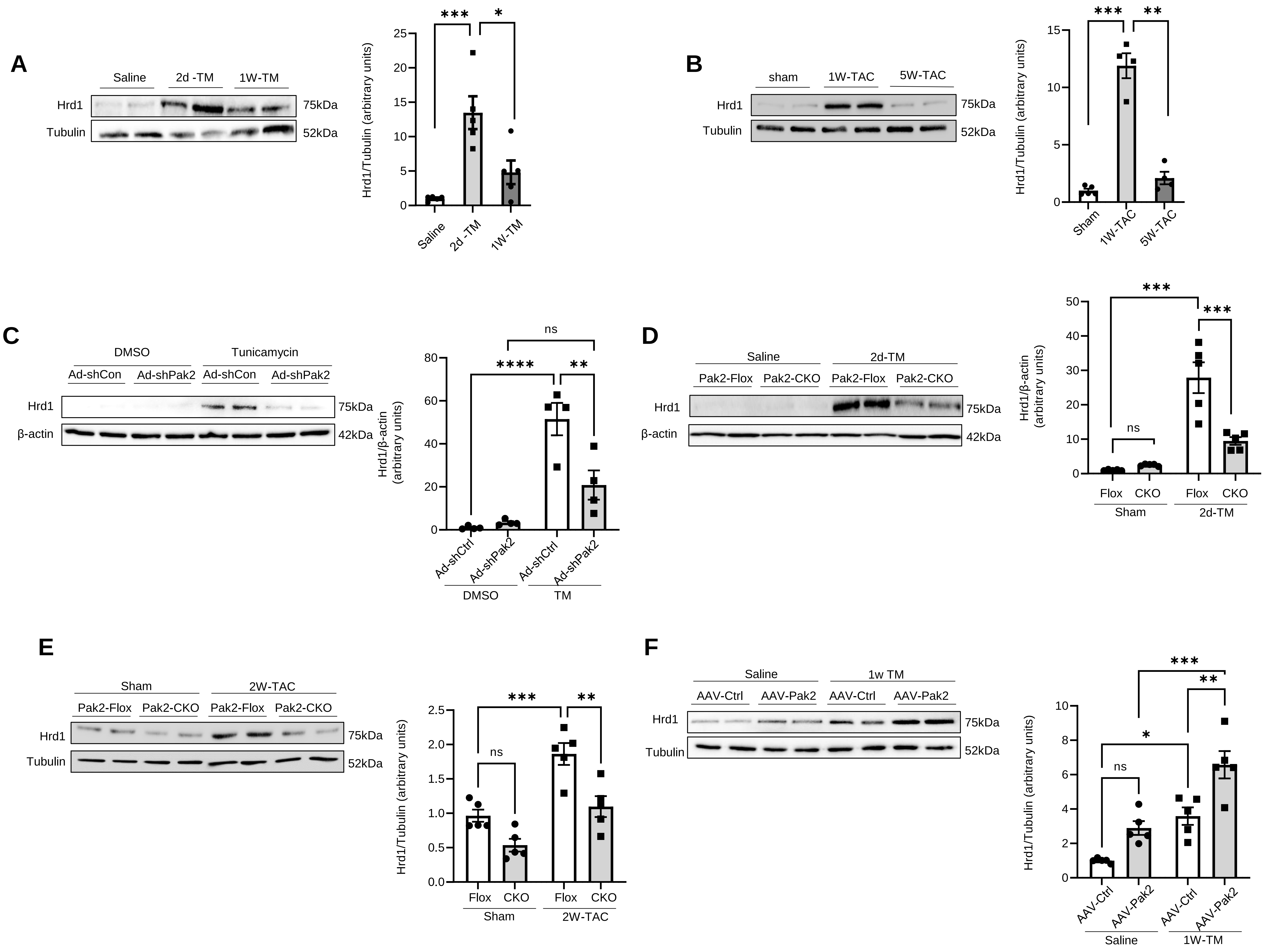

Supplementary Figure IX : Pak2 promotes Hrd1 expression in vivo and in vitro. (A) Immunoblots and quantification of Hrd1 from hearts of C57BL/6N mice subjected to 2 or 7 days TM intraperitoneal injection (n=5). **(B)** Immunoblots and quantification of Hrd1 from hearts of C57BL/6N mice subject to 1 or 5 week transverse aortic constriction (TAC) (n=4-5). **(C)** Immunoblots of Hrd1 in Pak2-knockdown (Ad-shPak2) ARCMs under tunicamycin (TM) treatment for 6h (n=4). Hrd1 levels was assessed in vivo in Pak2 depleted hearts (Pak2-CKO) under Tunicamycin **(D)** and TAC stress **(E)** and in AAV-9-Pak2-T402E-injected C57BL/6N mice **(F)** (n=5). 1-way or 2-way ANOVA with Bonferroni correction for post hoc comparisons were used for analyses. Data presented as mean±SEM.

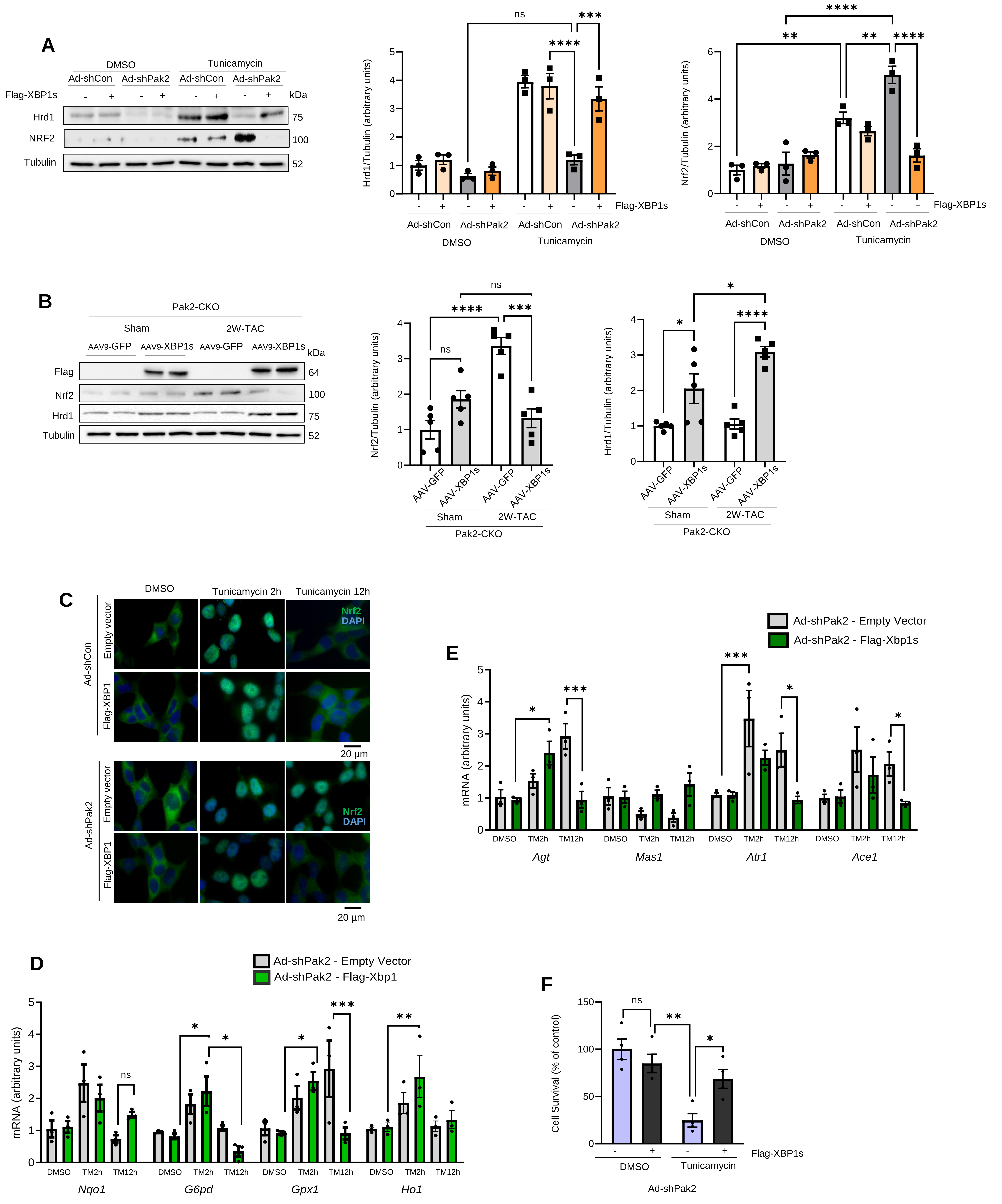

Supplementary Figure X: Pak2-dependent Nrf2 downregulation occurs via activation of the IRE1-XBP1 branch of the UPR. (A) Immunoblots and quantification of Nrf2 and Hrd1 in Pak2 depleted (Ad-shPak2) ARCMs transfected with Flag-tagged spliced XBP1 (XBP1s) cDNA and subjected to tunicamycin-induced ER stress (n=3). **(B)** Immunoblots and quantification of Nrf2 and Hrd1 in AAV9-XBP-1s injected Pak2-CKO hearts (n=5). **(C)** Nrf2 localisation was determined by immunofluorescence analysis in Flag-tagged XBP1s transfected Ad-shPak2 H9C2 cells under TM stress (n=3, scale bar:20µm). The effect of spliced XBP1 overexpression over Nrf2 conventional redox transcriptional targets **(D)** and RAAS components **(E)** in ad-shPak2 cardiomyocytes was determined by qPCR (n=3). **(F)** Cell viability was determined by MTT assay (n=4). 2-way ANOVA with Bonferroni correction for post hoc comparisons were used for analyses. Data presented as mean±SEM. *P<0.05, **P<0.01, ***P<0.001.

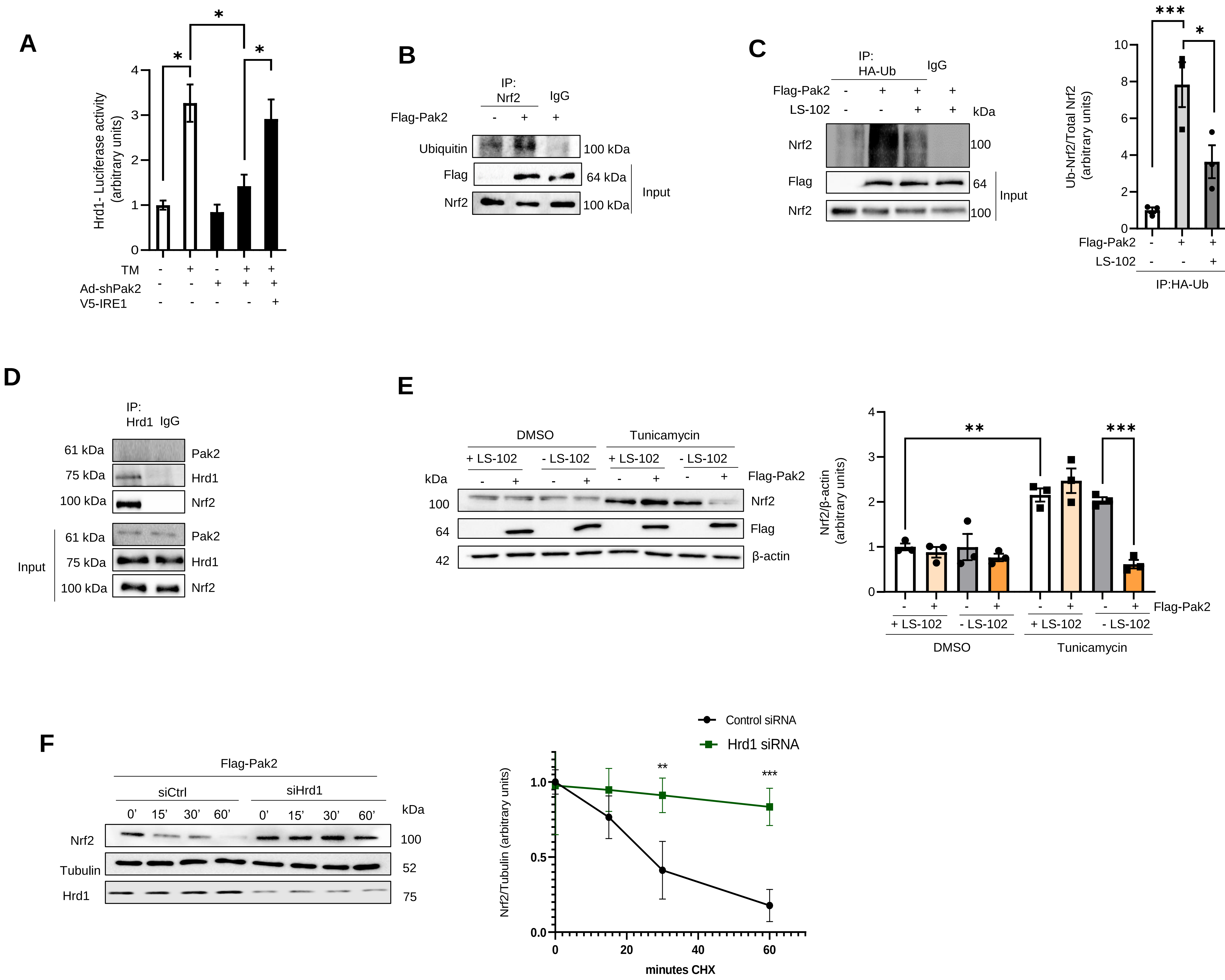

Supplementary Figure XI: Pak2-IRE-1 axis promotes Nrf2 ubiquitination. (A) The significant decrease in Hrd1 reporter luciferase activity detected in Pak2 knockdown (Ad-shPak2) H9C2 cells in response to Tunicamycin can be reverted by IRE1 expression (V5-IRE1) (n=5–6). **(B)** The effect of active Pak2 overexpression on Nrf2 ubiquitination was determined by Nrf2 immunoprecipitation followed by ubiquitin immunoblot (n=3). **(C)** The effect of active Pak2 overexpression and Hrd1 inhibition with LS-102 on Nrf2 ubiquitination was determined by HA-ubiquitin (HA-ub) immunoprecipitation and Nrf2 immunoblot (n=3). **(D)** Association of endogenous Nrf2 with Hrd1 was observed by immunoprecipitation in H9C2 cells. **(E)** Immunoblots and quantification showing Hrd1 inhibition with LS-102 prevents Pak2-mediated Nrf2 downregulation (n=3). **(F)** Nrf2 degradation in cardiomyocytes was assessed by cyclohexamide (CHX, 100 µg/mL) chases. Immunoblots showed that Hrd1 knockdown (siHrd1) prevents the observed increase Flag-Pak2-T402E dependent Nrf2 clearance in ARCMs (n=3). 1-way ANOVA with Bonferroni correction for post hoc comparisons were used for analyses. Data presented as mean±SEM.

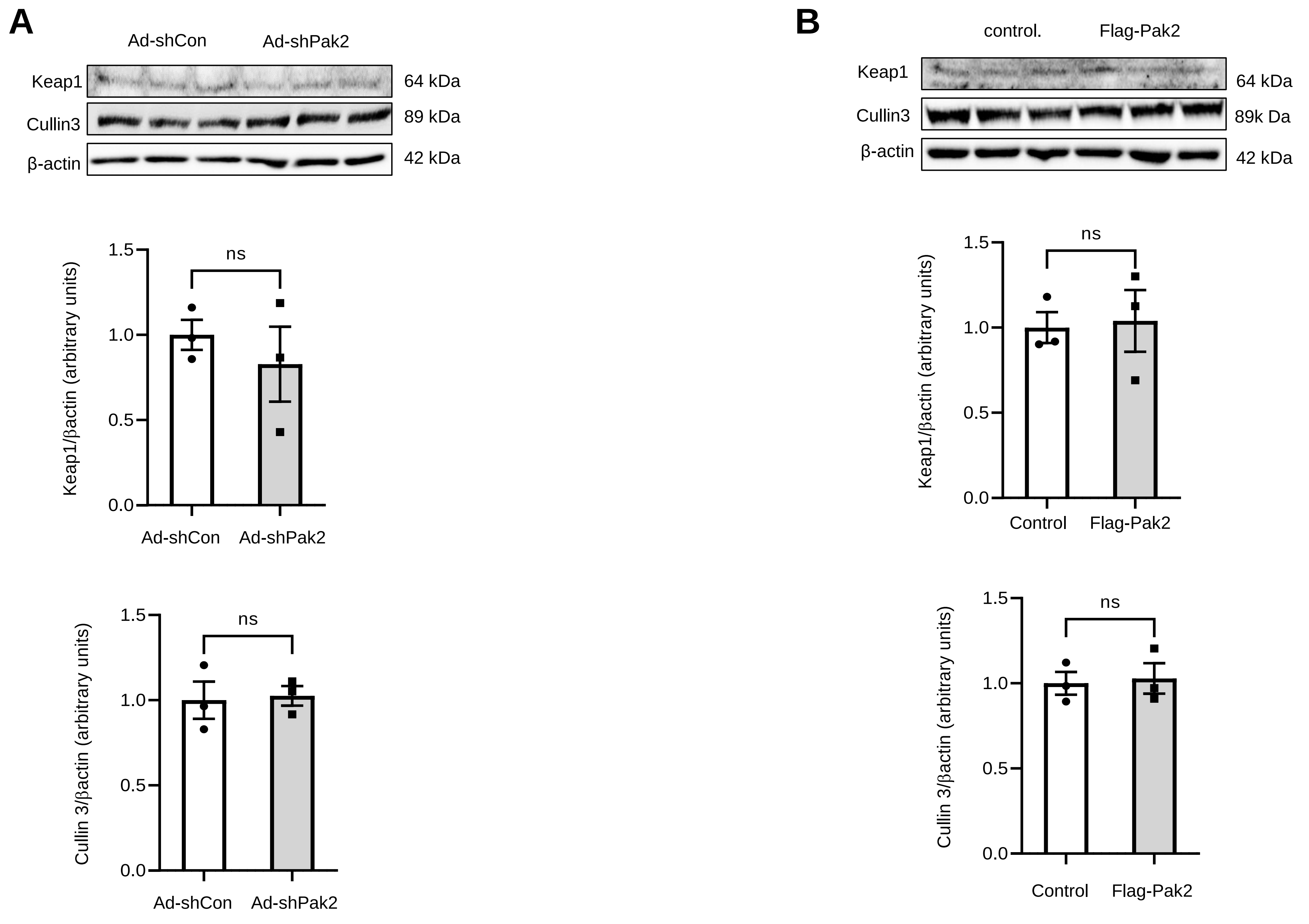

Supplementary Figure XII: Pak2 expression does not affect Keap1 nor Cullin3 levels in cardiomyocytes. (A), Immunoblots and quantification of Keap1 and Culin3 in control (Ad-shCon) and Pak2 knockdown (Ad-shPak2) H9C2 cells (n=3). **(B)** Immunoblots and quantification of Keap1 and Culin3 in control and active Pak2 overexpressing (Flag-Pak2) H9C2 cells (n=3). Student t test were used for analyses. Data presented as mean±SEM.

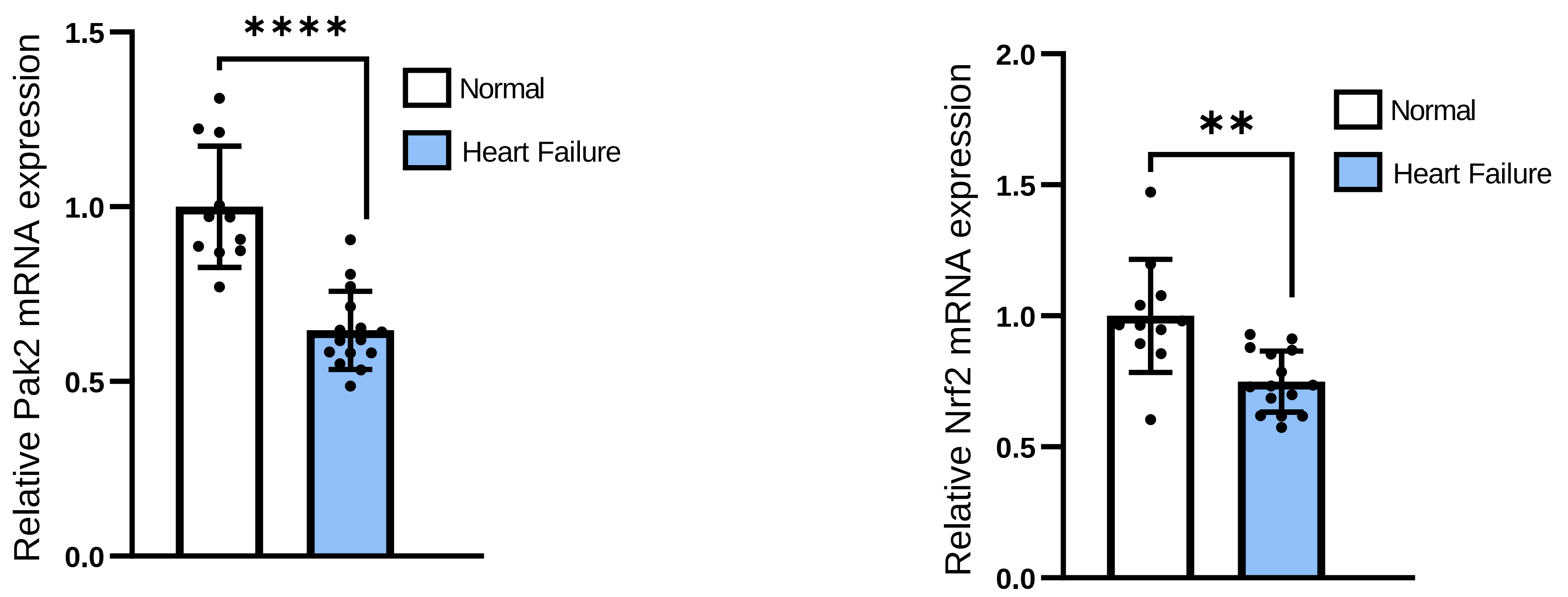

Supplementary Figure XIII: Loss of Pak2 is observed in heart tissue from patients with dilated heart failure. Pak2 and Nrf2 transcript levels from gene array assays of myocardial samples from patients undergoing cardiac transplantation bearing idiopathic dilated cardiomyopathy and from "normal" organ donors (n=11-15). Data are publicly accessible through GEO Series accession number GSE1145. Student t test were used for analyses. Data presented as mean±SEM.

Supplementary Table I

Antibodies used for immunoblotting

	Target	Species	Dilution	Catalogue code and Source
Primary Antibodies used	Anti-Pak2	Rabbit	1:1000	2608S, Cell Signalling Technology
	Anti-Nrf2	Rabbit	1:1000	Ab137550, Abcam
	Anti-Hrd1	Rabbit	1:1000	Nb100-2526, Novus
	Anti-PERK	Rabbit	1:1000	3192S Cell Signaling Technology
	Anti-pPERK	Rabbit	1:500	3179S, Cell Signaling Technology
	Anti-H3	Rabbit	1:1000	9715S, Cell Signaling Technology
	Anti-Flag	Mouse	1:3000	F7425, Sigma-Aldrich
	Anti-pPak2	Rabbit	1:1000	2607S, Cell Signalling Technology
	Anti-β-actin	n/a	1:5000	Ab20272, Abcam
	Anti-α-Tubulin	Rabbit	1:3000	T6199, Sigma
	Anti-cleaved caspase 3	Rabbit	1:500	9661S, Cell Signalling Technology
	Anti-Bip/GRP78	Rabbit	1:1000	Ab21685, abcam
	Anti-Chop	Mouse	1:1000	2895S, Cell Signalling Technology
	Anti-ATF6	Rabbit	1:1000	Ab37149, Abcam
	Anti-IRE1	Rabbit	1:1000	Ab37073, Abcam
	Anti-phospho-IRE1	Rabbit	1:500	Ab48187, Abcam
	Anti-XBP1s	Rabbit	1:1000	Ab37152, Abcam
	Anti-HIF1α	Rabbit	1:1000	Sc-13515, Santa Cruz
	Anti-NF-κB	Rabbit	1:1000	8242S, Cell Signaling Technology
	Anti-HA	Rabbit	1:2000	2367S, Cell Signaling Technology
	Anti-Ubiquitin	Rabbit	1:1000	3933S, Cell Signaling Technology
	Anti-Keap1	Rabbit	1:1000	8047S, Cell Signaling Technology
	Anti-Cul3	Rabbit	1:1000	2759S, Cell Signaling Technology
	Anti-Atr1	Rabbit	1:1000	25343-1-AP, Proteintech
	Anti-Mas1	Rabbit	1:1000	Sc390453, Santa Cruz Biotech
	Anti-eIF2α	Rabbit	1:1000	9722S, Cell Signaling Technology
	Anti-peIF2α	Rabbit	1:1000	9721S Cell Signaling Technology
	Anti-Angiotensinogen	Rabbit	1:500	79299S Cell Signaling Technology
Secondary Antibodies used	Anti-Rabbit	n/a	1:1000	7074S, Cell Signalling Technology
	Anti-Mouse	n/a	1:1000	7076S, Cell Signalling Technology

Supplementary Table II

Echocardiographic parameters of Pak2-Flox and Pak2-CKO mice following TAC

	Sham		2W-TAC	
	Pak2-Flox	Pak2-CKO	Pak2-Flox	Pak2-CKO
IVS;d (mm)	0.88±0.03	0.87±0.04	1.09±0.06	1.15±0.03
LVID;d (mm)	3.62±0.09	3.69±0.12	3.94±0.09	4.23±0.10
LVPW;d (mm)	0.79±0.03	0.82±0.02	1.15±0.04	1.30±0.04*
IVS;s (mm)	1.01±0.03	1.08±0.03	1.22±0.06	1.27±0.05
LVID;s (mm)	2.28±0. 12	2.37+0.14	3.05+0.12	3.70+0.15*
LVPW;s (mm)	1.02±0.05	1.05±0.04	1.26±0.05	1.34±0.05
Heart Rate (bpm)	434.16±13.38	442.33+14.15	432.60±5.77	421.85±10.59
% EF	58.14±4.69	62.07±3.55	50.41±1.82	36.80±2.63*
% FS	36.99±2.32	36.22±2.33	22.69±1.63	12.75±1.43#
Body weight (g)	28.5±1.6	29.3±2.1	28.7±1.3	29.3±1.5
Heart Weight (g)	0.18±0.06	0.17±0.05	0.23±0.05	0.26±0.04
HW/BW (mg/g)	6.31±0.89	5.80±0.48	8.01±0.37	8.87±0.72*

IVS;d, Interventricular Septal Thickness at Diastole; LVID;d, Left ventricular internal diameter end diastole; LVPW;d, Left ventricle posterior wall end diastole; IVS;s, Interventricular Septal Thickness at systole; LVID;s, Left ventricular internal diameter end systole; LVPW;d, Left ventricle posterior wall end systole; EF, ejection fraction; FS, fraction shortening; HW/BW, Heart Weight to Body weight ratio. n=5-7 per group. Data are presented as means ± SEM, *P≤0.05, #P≤0.01 versus Pak2-Flox-TAC mice.

Supplementary Table III

Echocardiographic parameters of mice injected with AAV-GFP or AAV-Pak2 following TM injection

	Saline		1W-Tunicamycin	
	AAV9-GFP	AAV9-Pak2-T402E	AAV9-GFP	AAV9-Pak2-T402E
IVS;d (mm)	0.78±0.05	0.81±0.04	0.98±0.05	0.85±0.03
LVID;d (mm)	3.26±0.15	3.27±0.16	3.86±0.11	3.33±0.11
LVPW;d (mm)	0.83±0.02	0.86±0.03	1.01±0.06	0.82±0.04*
IVS;s (mm)	1.07±0.04	0.96±0.04	1.28±0.03	1.16±0.05
LVID;s (mm)	2.05±0.10	2.11±0.14	3.01±0.14	2.27±0.07
LVPW;s (mm)	1.12±0.05	1.18±0.04	1.21±0.06	1.13±0.05
Heart Rate (bpm)	425.8±5.3	420.8±3.6	417.2±4.4	418.4±4.8
% EF	62.25±3.46	65.32±3.38	45.12±4.32	63.32±2.97*
% FS	36.93±1.92	35.59±1.73	22.11±2.56	31.72±1.60*
Body weight (g)	27.5±1.5	28.6±2.4	27.7±1.8	29.1±1.4
Heart Weight (g)	0.18±0.04	0.18±0.03	0.22±0.03	0.18±0.06
HW/BW (mg/g)	6.54±0.55	6.29±0.36	7.94±0.38	6.18±0.67

IVS;d, Interventricular Septal Thickness at Diastole; LVID;d, Left ventricular internal diameter end diastole; LVPW;d, Left ventricle posterior wall end diastole; IVS;s, Interventricular Septal Thickness at systole; LVID;s, Left ventricular internal diameter end systole; LVPW;d, Left ventricle posterior wall end systole; EF, ejection fraction; FS, fraction shortening; HW/BW, Heart Weight to Body weight ratio. n=5-7 per group. Data are presented as means ± SEM, *P≤0.05, #P≤0.01 versus AAV9-GFP-TM mice.

Supplementary Table IV

Echocardiographic parameters of mice injected with AAV-GFP or AAV-Pak2 following TAC

	Sham		4W-TAC	
	AAV-GFP	AAV-Pak2	AAV-GFP	AAV-Pak2
IVS;d (mm)	0.83±0.04	0.87±0.06	1.12±0.09	0.92±0.05
LVID;d (mm)	3.56±0.11	3.38+0.14	3.84±0.09	3.67±0.12
LVPW;d (mm)	0.68±0.03	0.63±0.02	0.97±0.06	0.69±0.03#
IVS;s (mm)	0.99±0.08	1.06±0.05	1.27±0.05	1.13±0.07
LVID;s (mm)	2.13±0.14	2.00±0.21	3.09±0.15	2.44±0.21*
LVPW;s (mm)	0.97±0.04	0.93±0.03	1.13±0.05	1.05±0.04
Heart Rate (bpm)	440.60±4.19	421.80±10.63	432.17±4.31	426.14±5.67
% EF	68.26±3.91	73.39±5.81	40.00±4.67	59.68±5.65*
% FS	40.42±2.58	41.55±3.89	19.79±2.64	34.27±4.24*
Body weight (g)	30.2±1.8	31.1±1.6	30.3±2.3	29.7±2.2
Heart Weight (g)	0.17±0.03	0.18±0.04	0.24±0.02	0.19±0.01
HW/BW (mg/g)	5.62±0.39	5.78±0.81	8.03±0.54	6.39±0.73

IVS;d, Interventricular Septal Thickness at Diastole; LVID;d, Left ventricular internal diameter end diastole; LVPW;d, Left ventricle posterior wall end diastole; IVS;s, Interventricular Septal Thickness at systole; LVID;s, Left ventricular internal diameter end systole; LVPW;d, Left ventricle posterior wall end systole; EF, ejection fraction; FS, fraction shortening; HW/BW, Heart Weight to Body weight ratio. n=5-7 per group. Data are presented as means ± SEM, *P≤0.05, #P≤0.01 versus AAV-GFP-TAC mice.

Source Western Blots by Figure

1 F

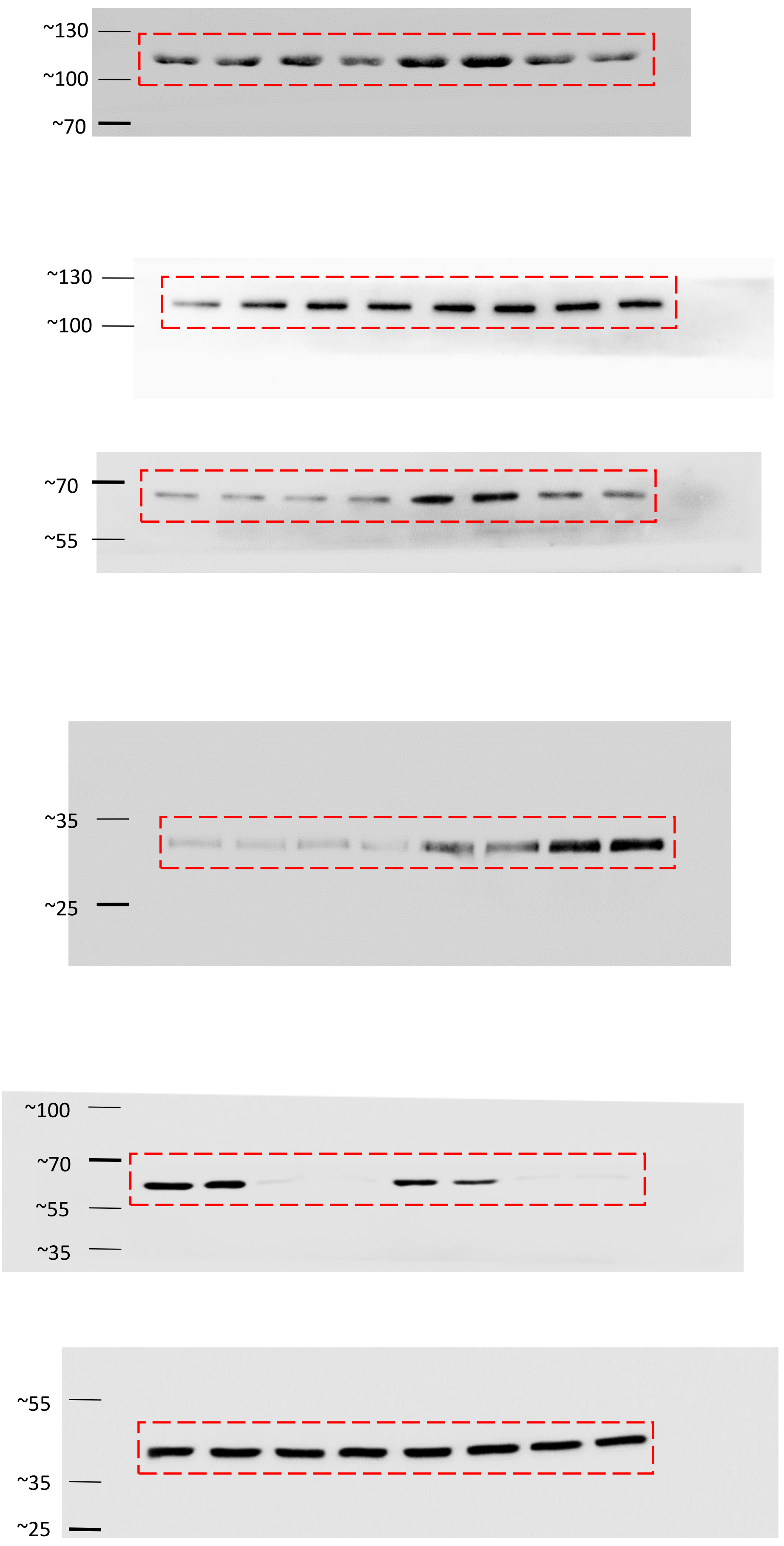

2 B

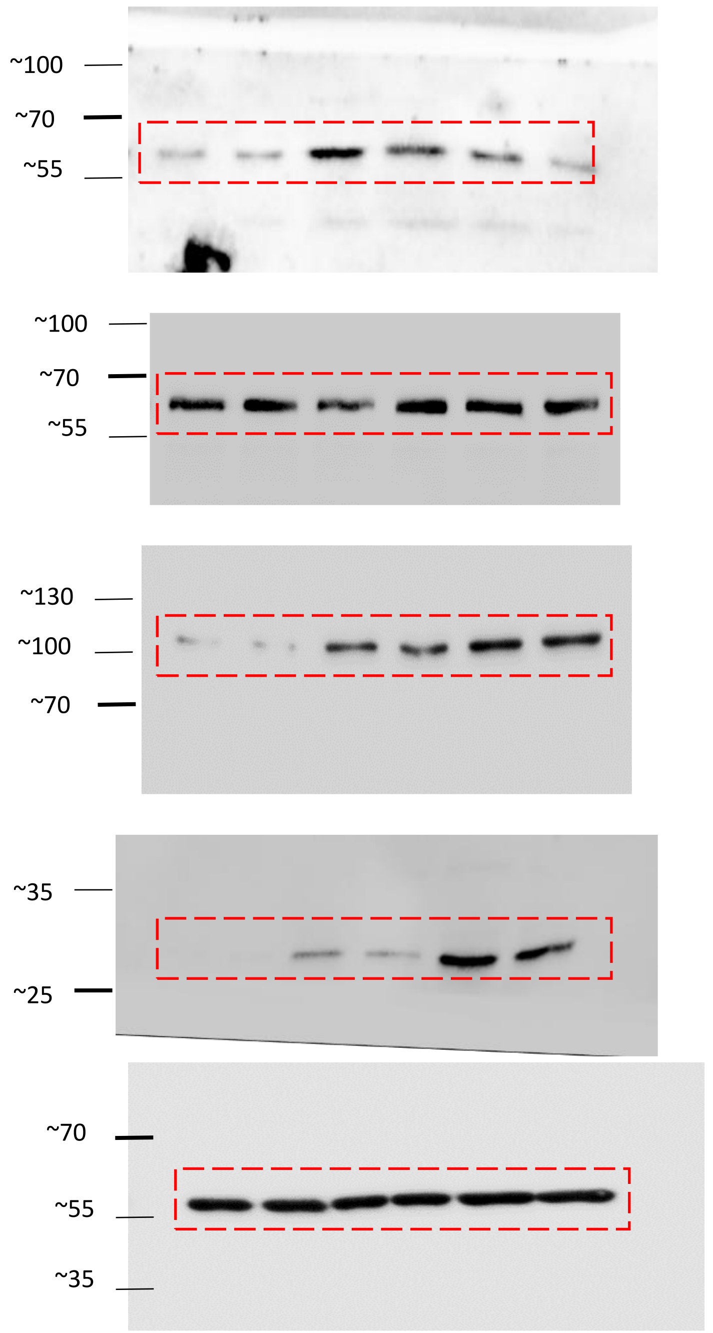

2 H

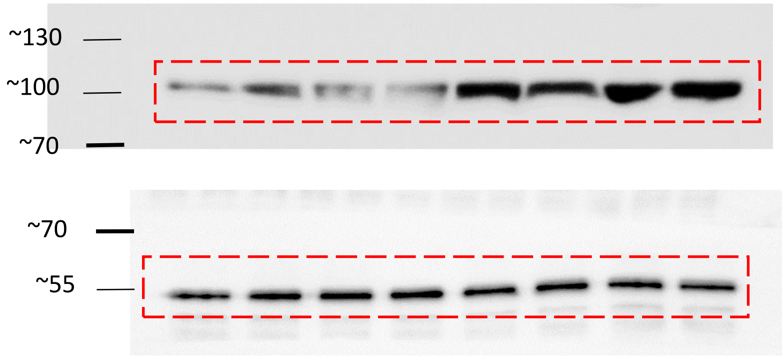

2 F

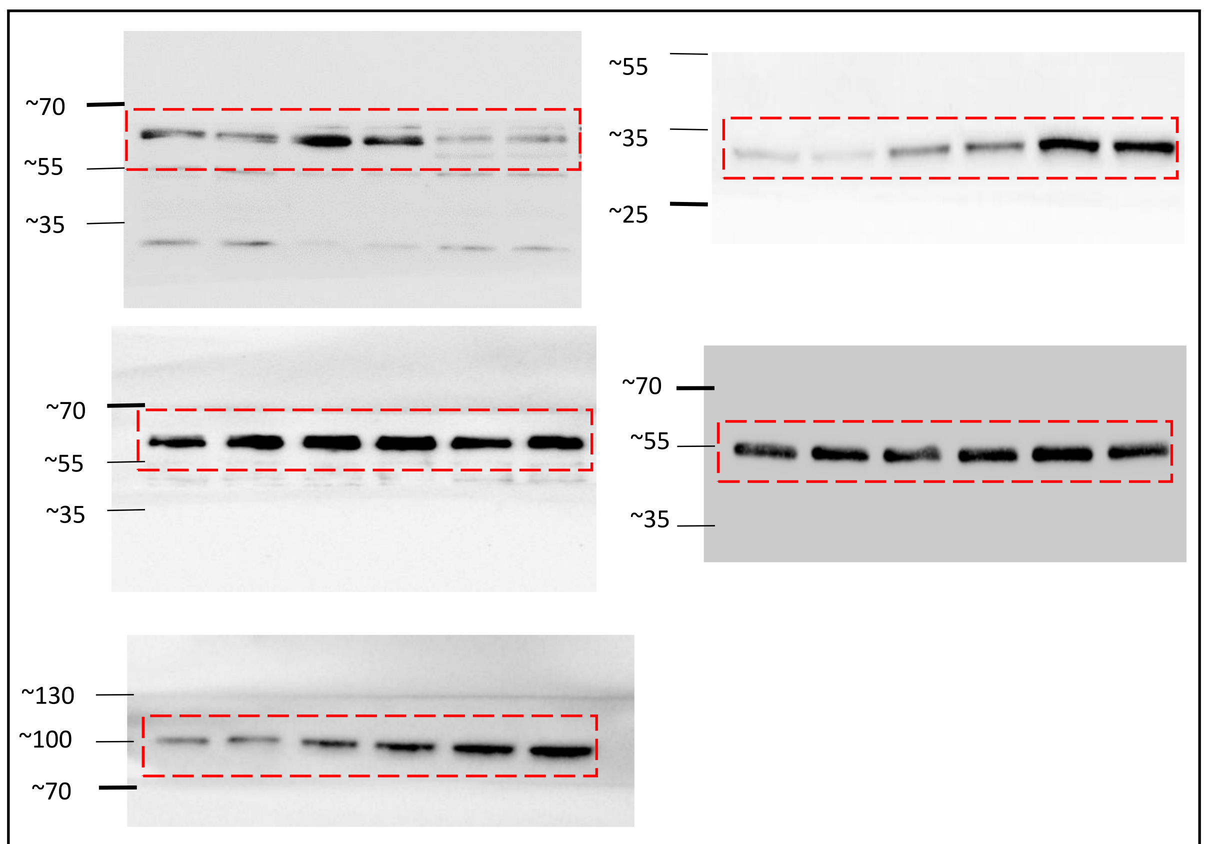

3 F

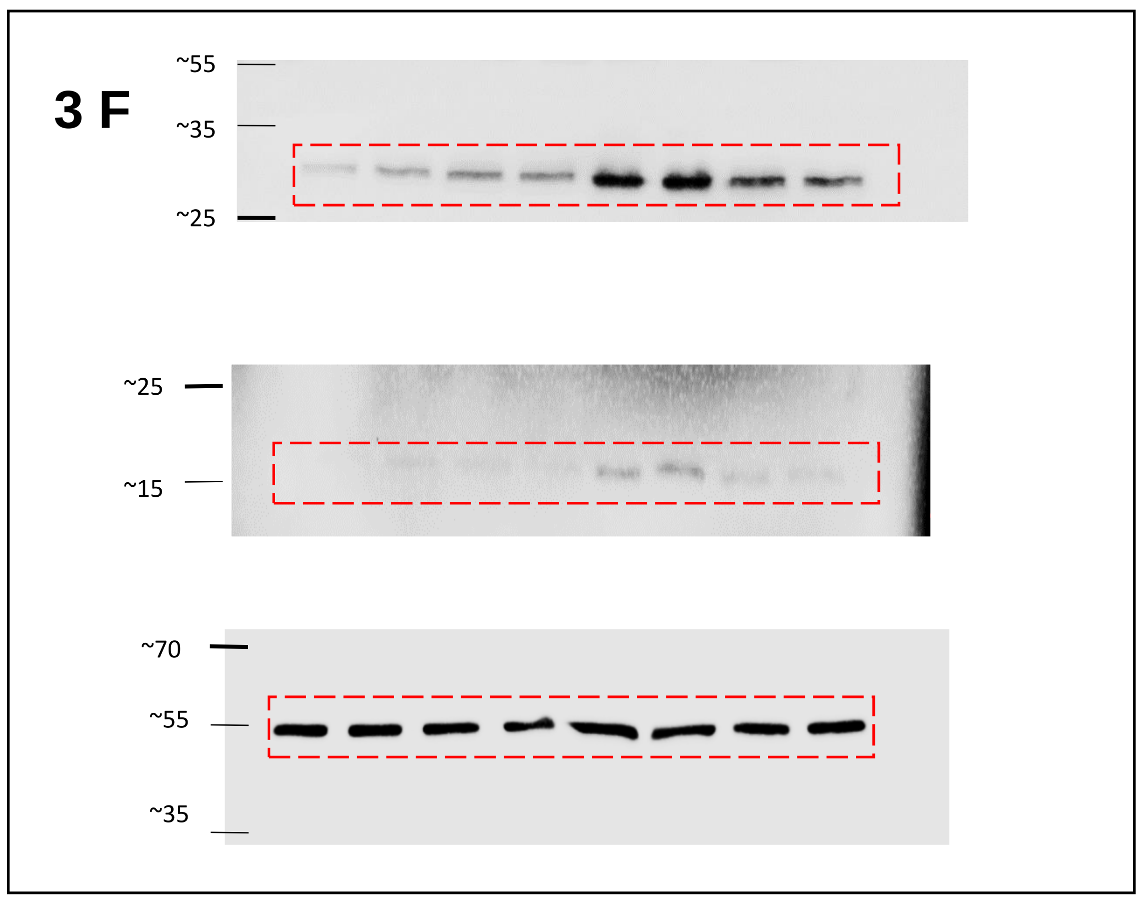
~55
~35
~25
~25
~15
~70
~55
~35

3 H

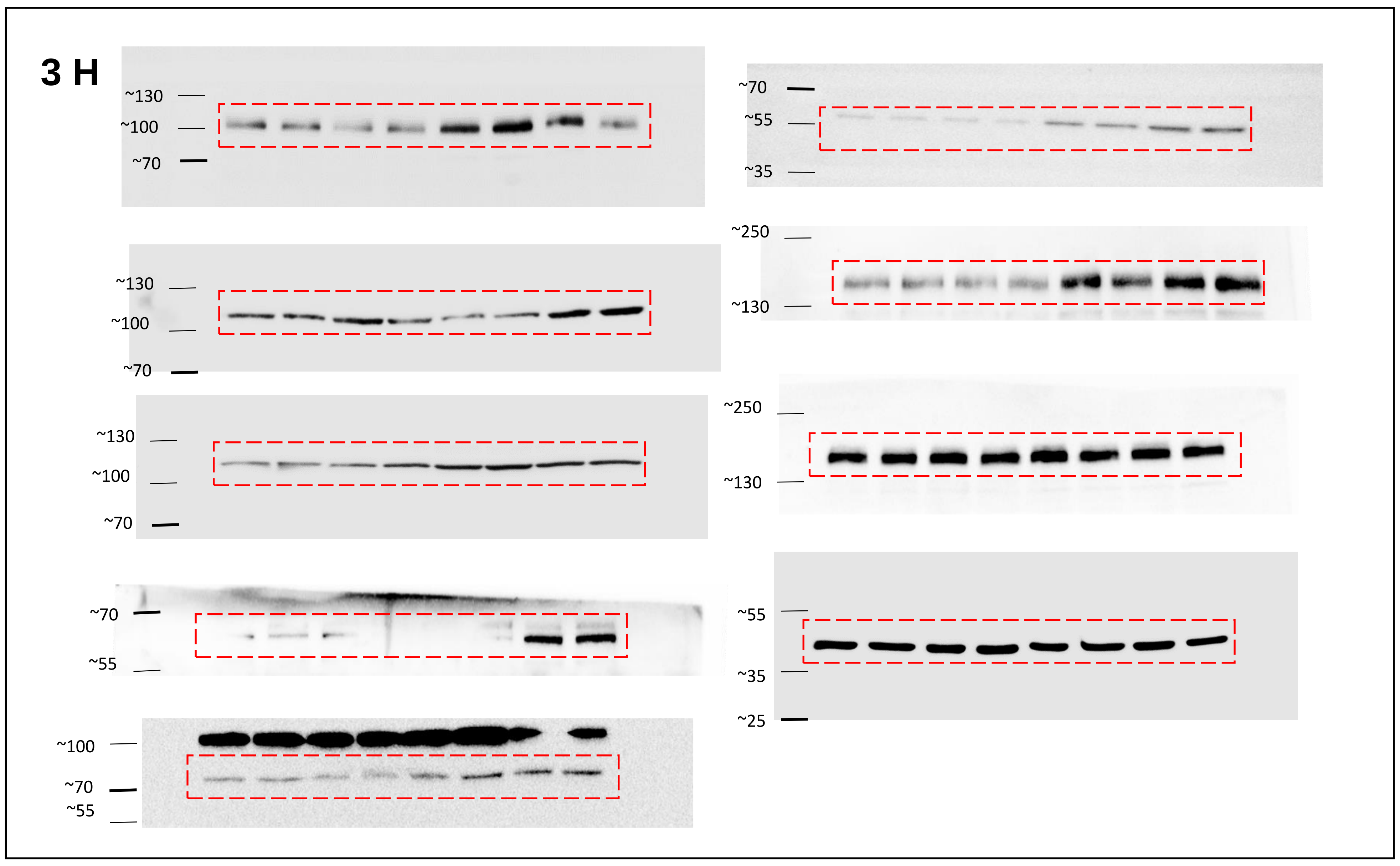
~130
~100
~70
~70
~55
~35
~130
~100
~70
~250
~130
~130
~100
~70
~250
~130
~70
~55
~55
~35
~25
~100
~70
~55

4 A

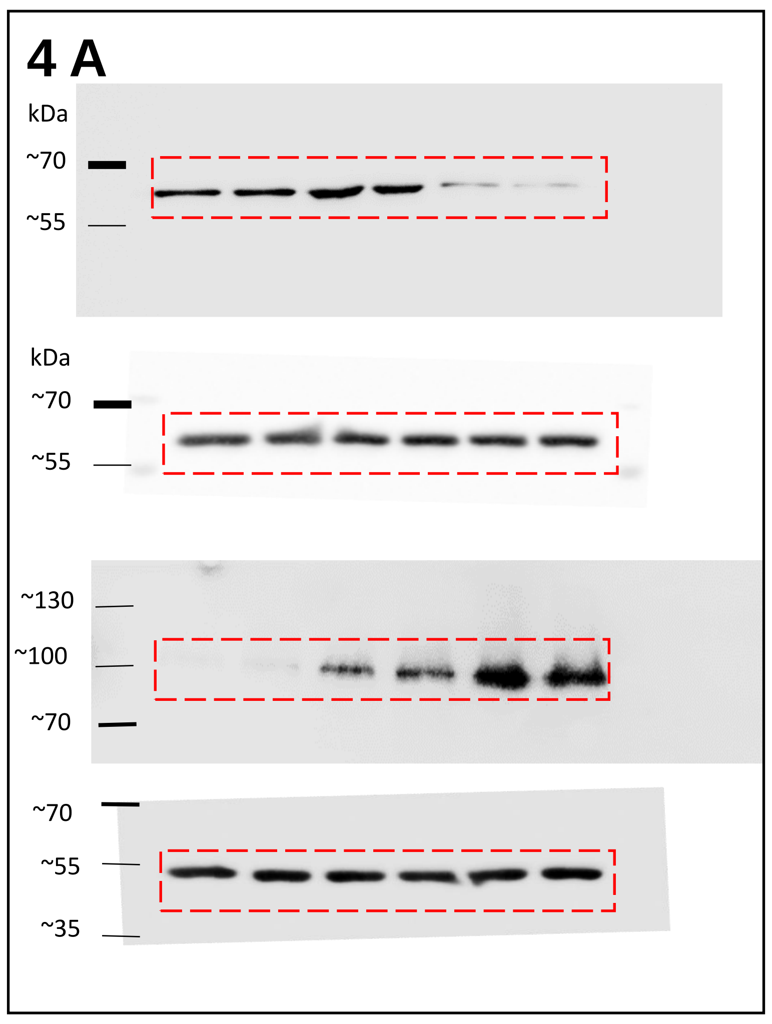
kDa
~70
~55
kDa
~70
~55
~130
~100
~70
~70
~55
~35

4 B

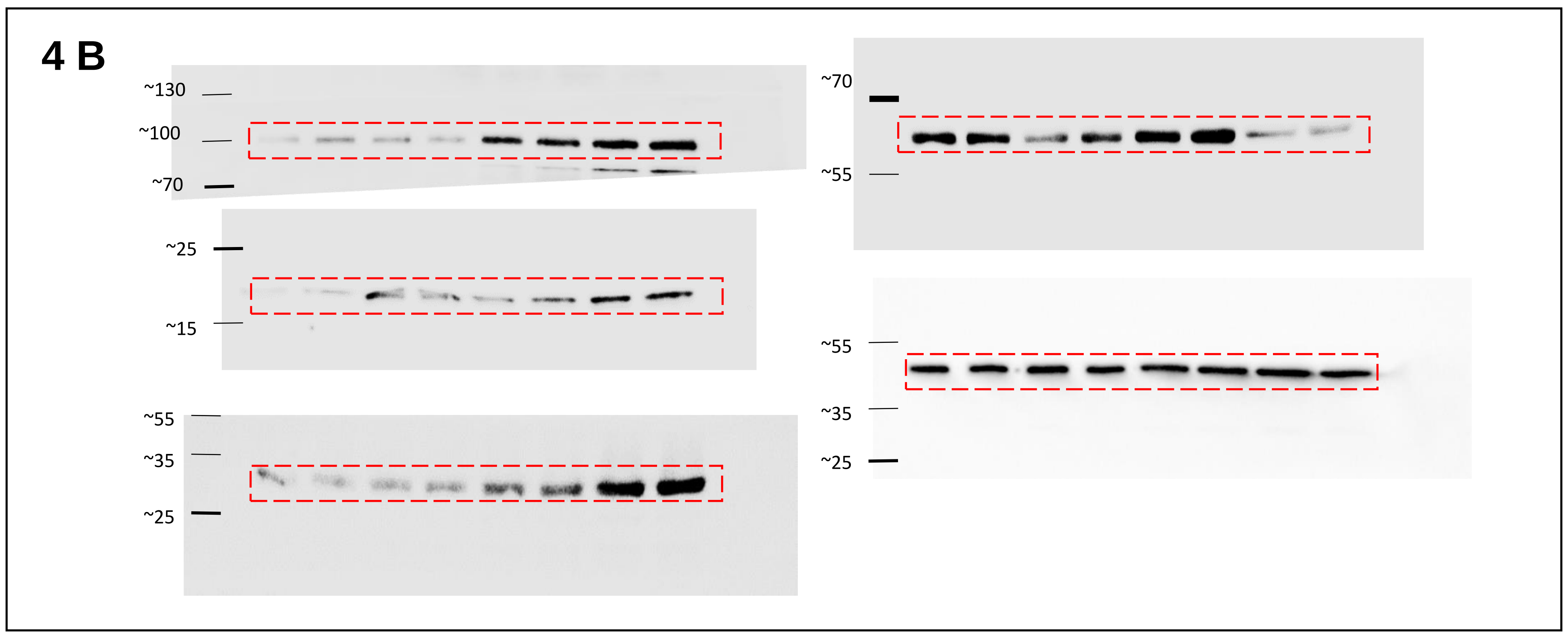
~130
~100
~70
~70
~55
~25
~15
~55
~35
~25
~55
~35
~25

4 C

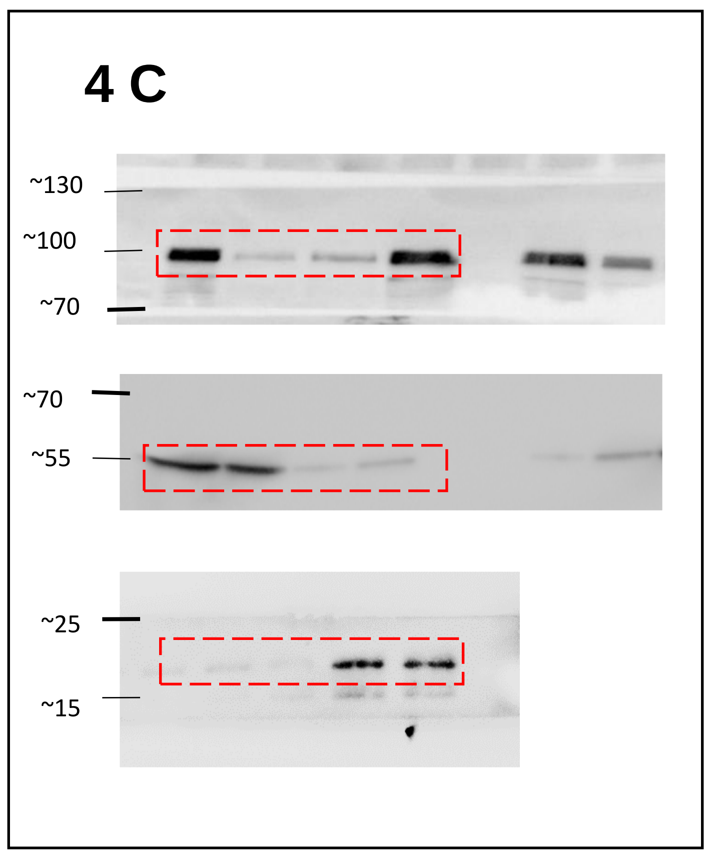

4 D

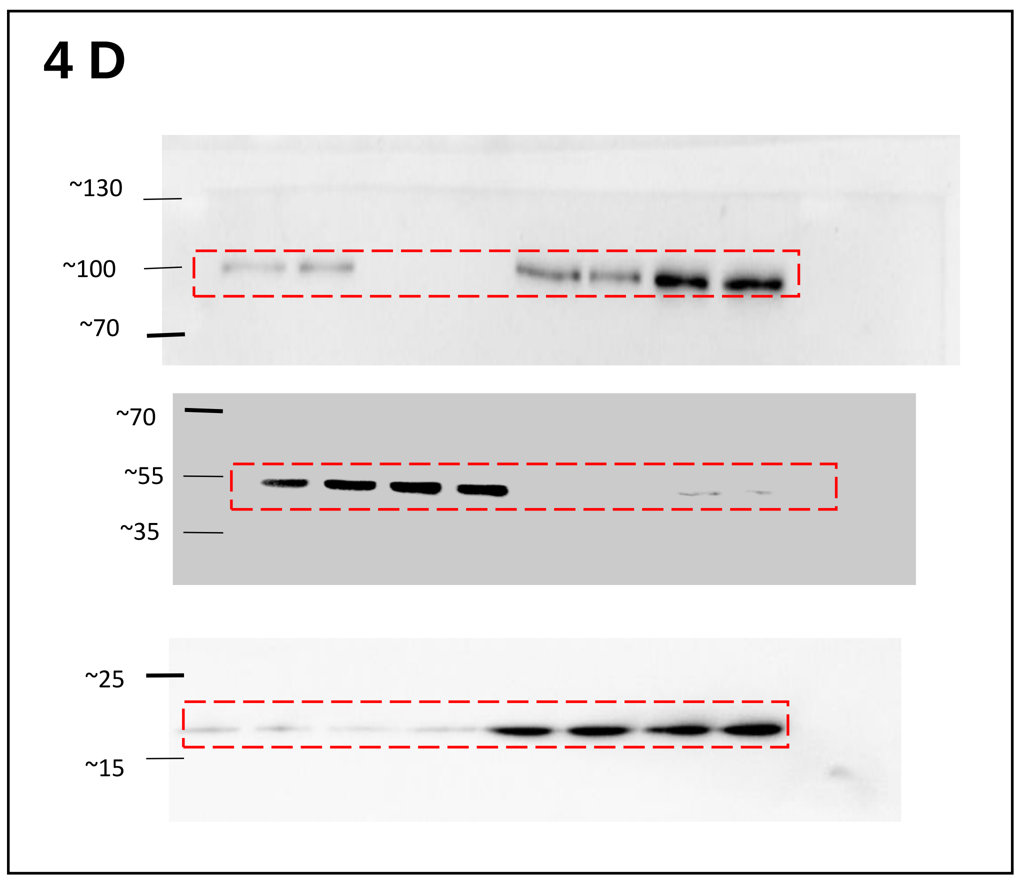

5 B

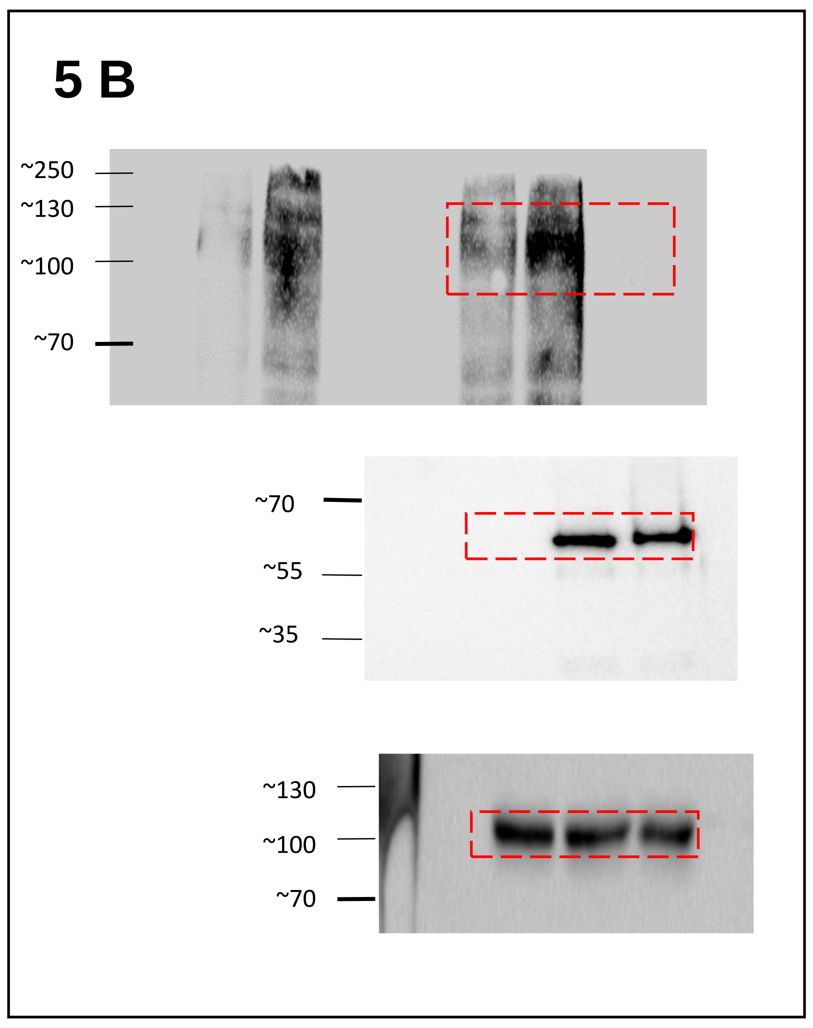

5 C

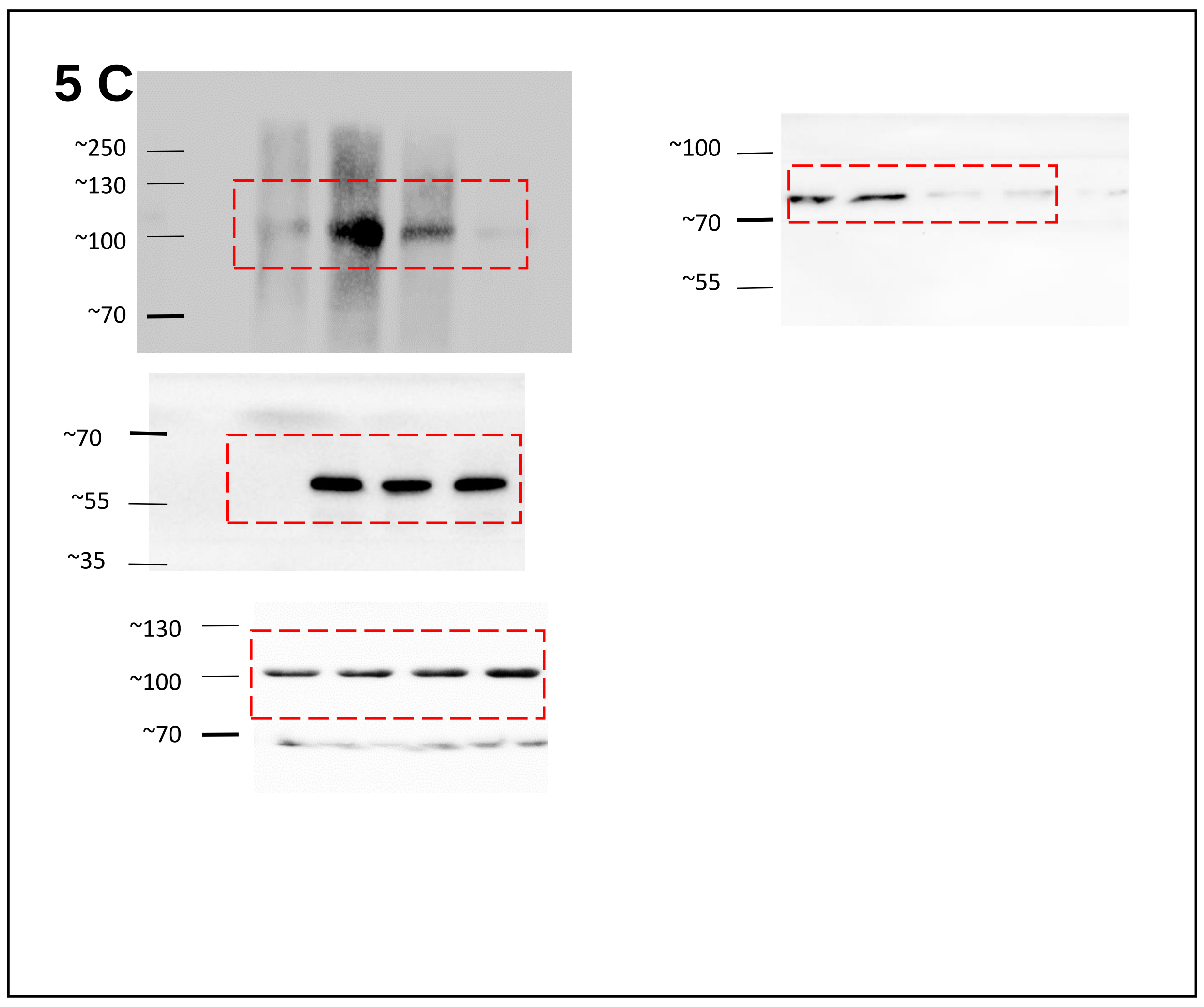

5 D

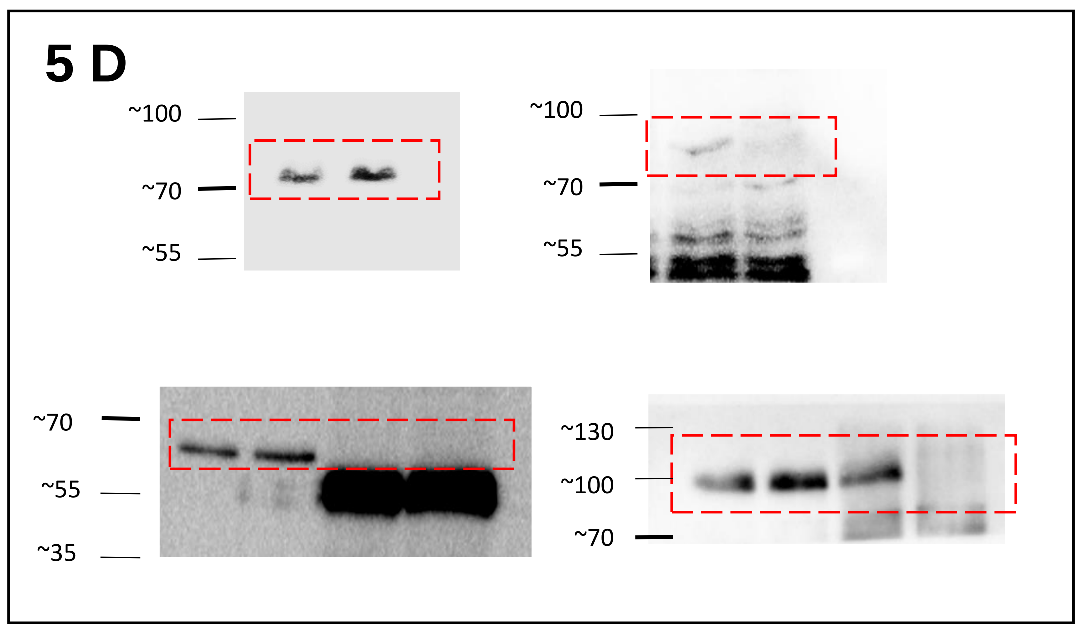

5 E

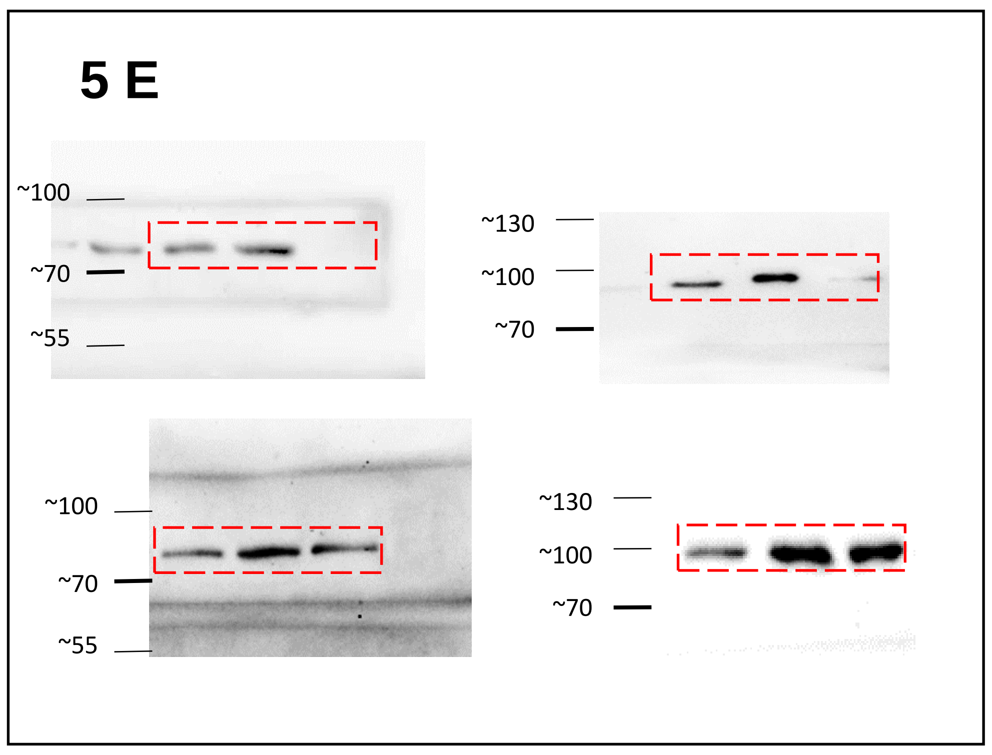

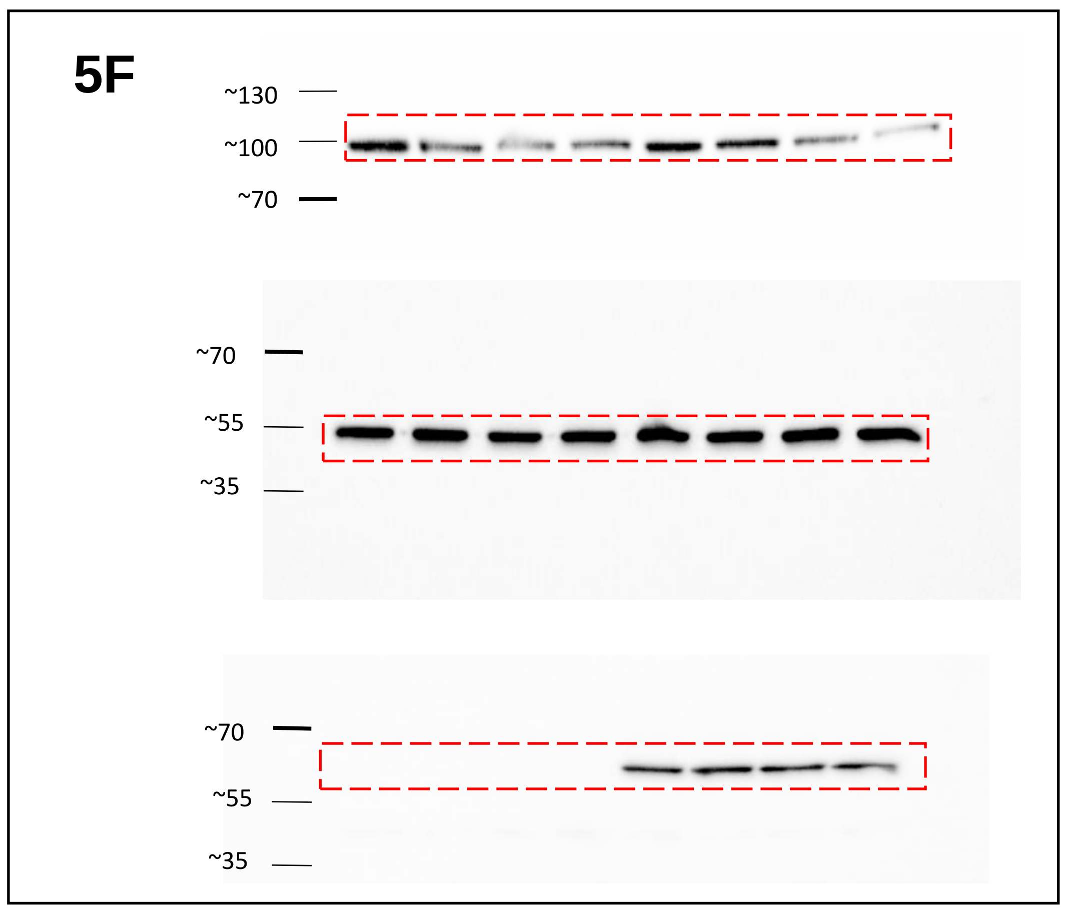
5F
~130
~100
~70
~70
~55
~35
~70
~55
~35

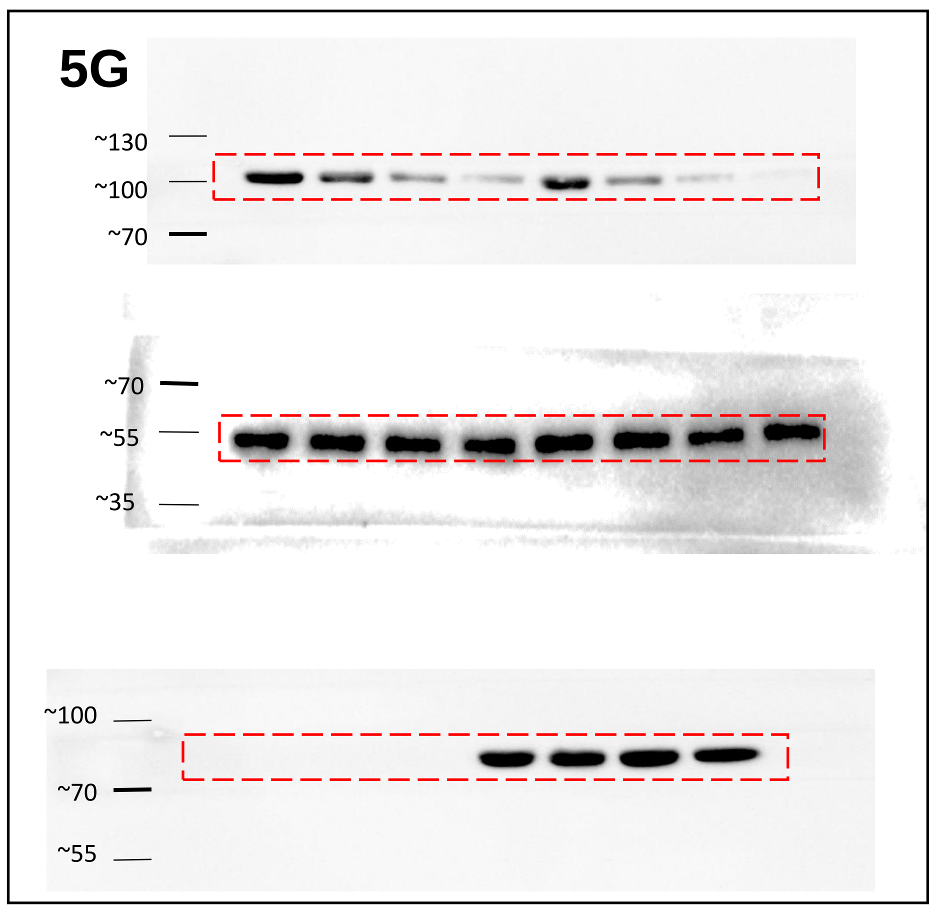
5G
~130
~100
~70
~70
~55
~35
~100
~70
~55

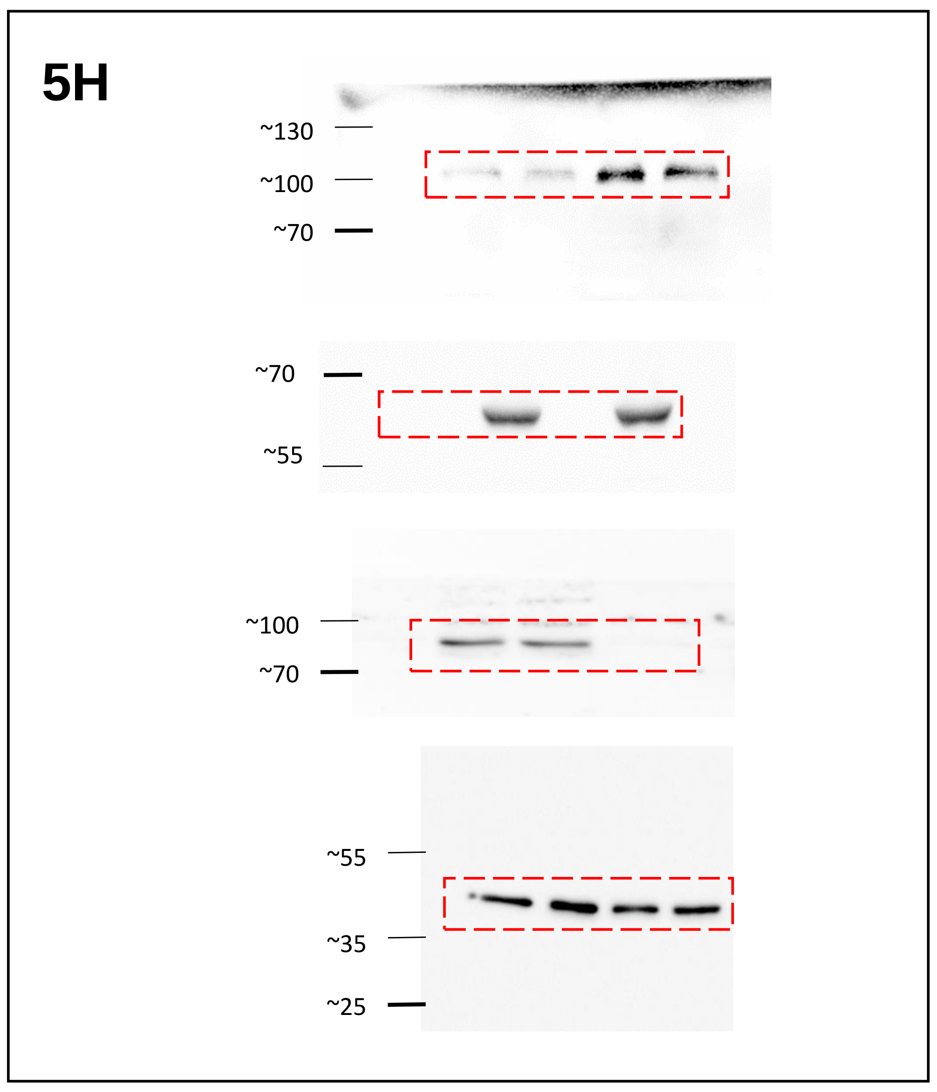
5H
~130
~100
~70
~70
~55
~100
~70
~55
~35
~25

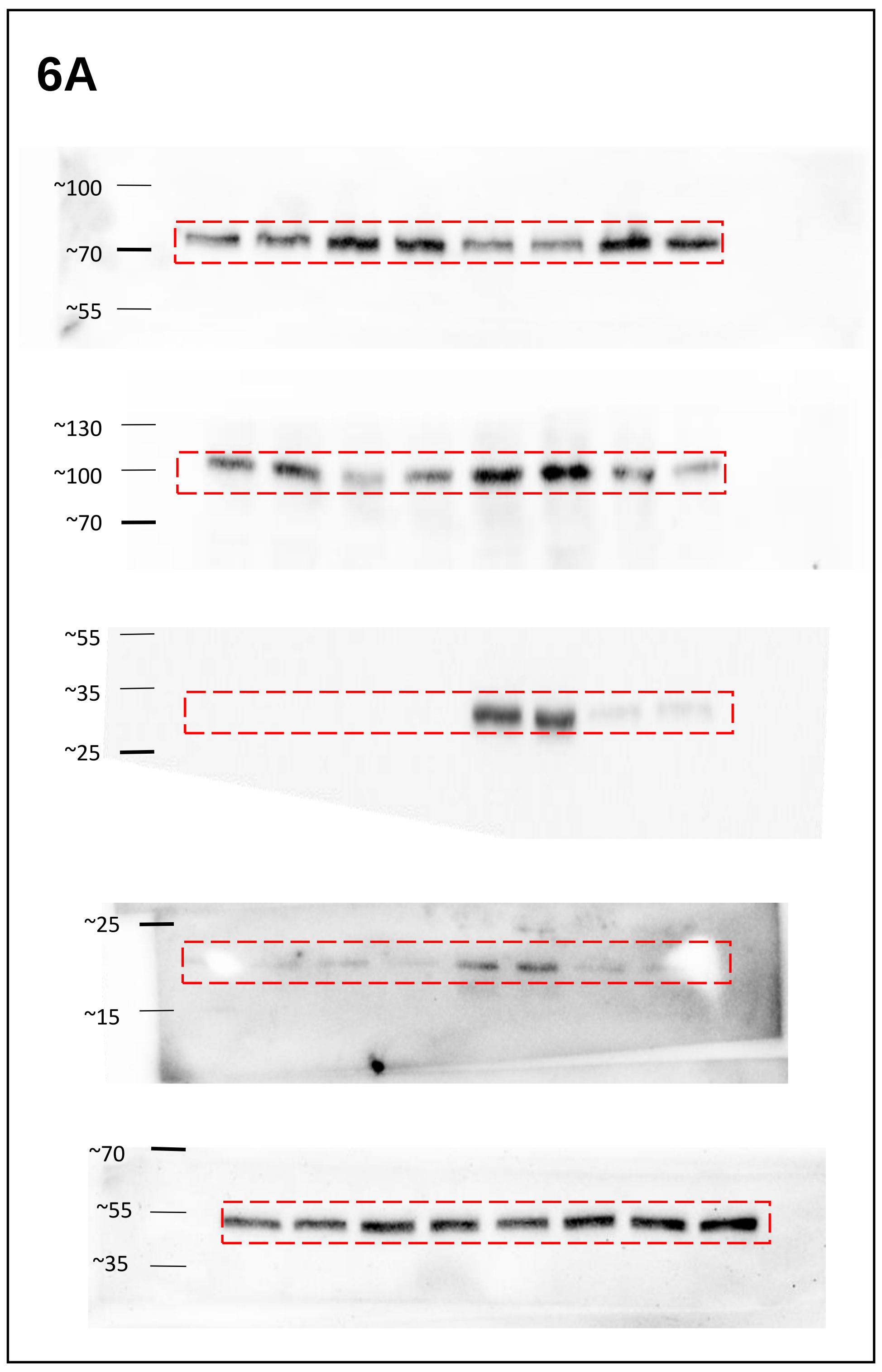
6A
~100
~70
~55
~130
~100
~70
~55
~35
~25
~25
~15
~70
~55
~35

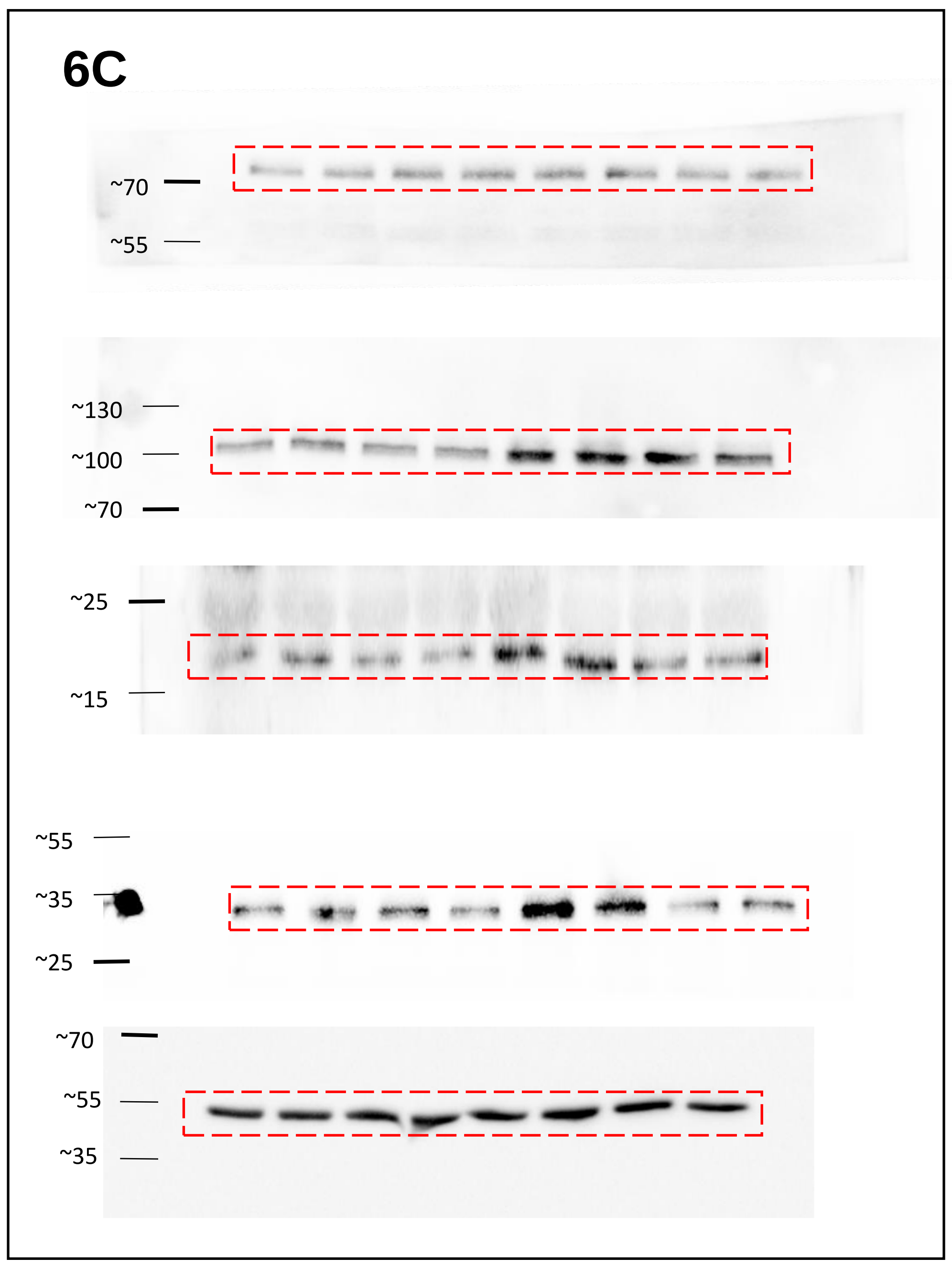
6C
~70
~55
~130
~100
~70
~25
~15
~55
~35
~25
~70
~55
~35

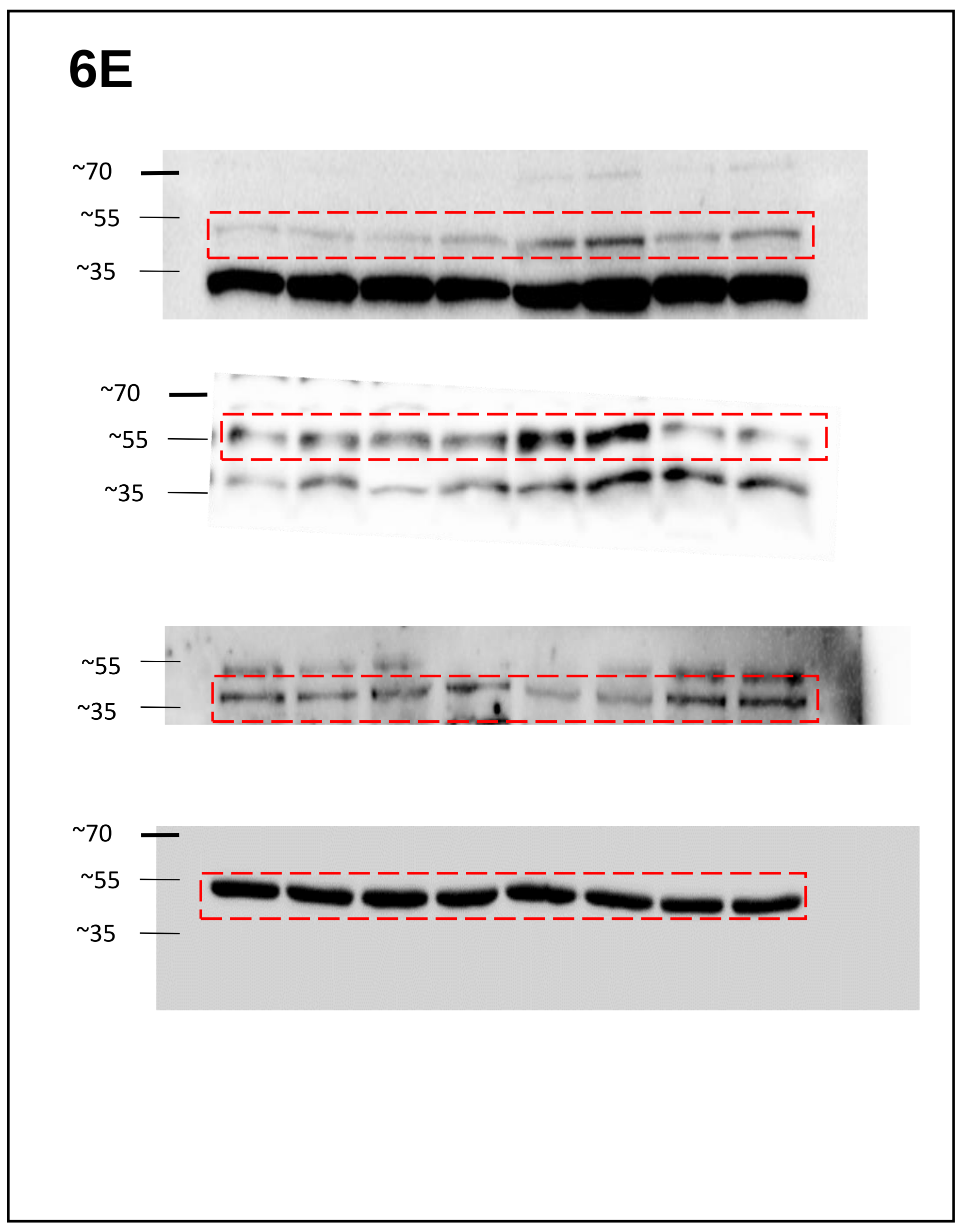
6E
~70
~55
~35
~70
~55
~35
~55
~35
~70
~55
~35

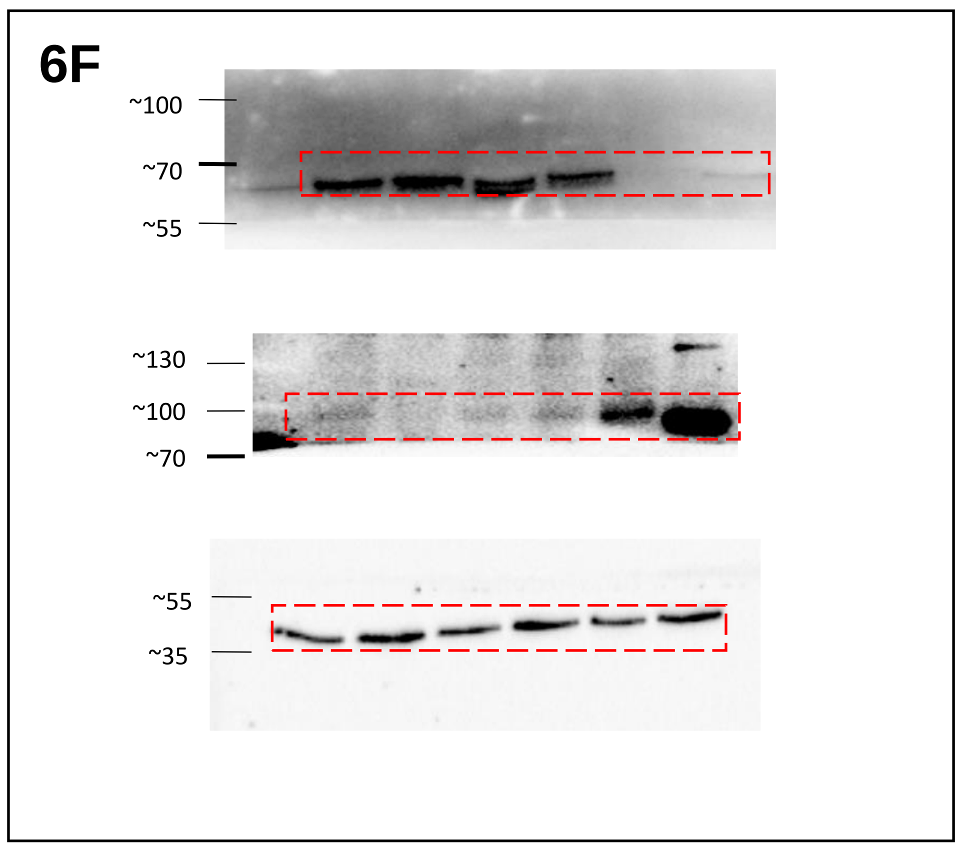
6F
~100
~70
~55
~130
~100
~70
~55
~35
